# Influence of Domain Size and Support Composition on the Reducibility of SiO_2_ and TiO_2_ Supported Tungsten Oxide Clusters

**DOI:** 10.1021/acs.jpcc.4c03652

**Published:** 2024-08-14

**Authors:** Konstantin Mamedov, Anukriti Shrestha, Colby A. Whitcomb, Christopher Paolucci, Robert J. Davis

**Affiliations:** Department of Chemical Engineering, University of Virginia, Charlottesville, Virginia 22903, United States

## Abstract

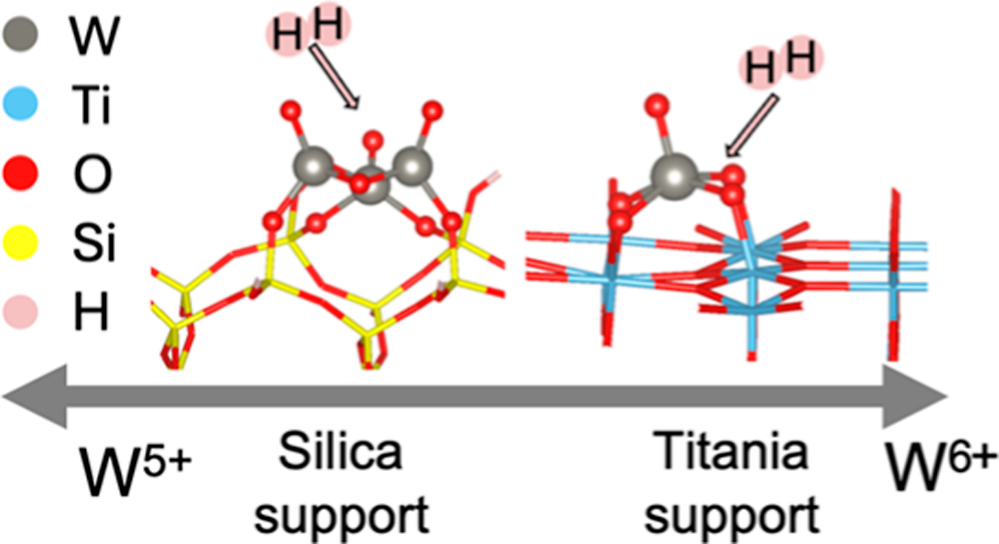

Supported tungsten oxides are widely used in a variety of catalytic reactions. Depending on the support, the cluster size, oxidation state, reducibility and speciation of the tungsten oxides can widely differ. When promoted with a platinum group metal, the resulting spillover of hydrogen may facilitate the reduction of supported tungsten oxide species, depending on the support. High resolution scanning transmission electron microscopy imaging showed nanometer scale WO_*x*_ clusters were synthesized on SiO_2_ whereas highly dispersed species were formed on TiO_2_. Results from H_2_-temperature-programmed reduction showed the presence of Pd lowered the initial reduction temperature of SiO_2_-supported WO_*x*_ species but interestingly did not affect that of TiO_2_-supported WO_*x*_. X-ray photoelectron and absorption spectroscopies showed the W atoms in SiO_2_-supported WO_*x*_ species reduce from a +6 oxidation state to primarily +5 after thermal treatment in 5% H_2_, while the fraction of W in the +5 oxidation state was relatively unaffected by reduction treatment of TiO_2_-supported WO_*x*_. The unusual behavior of TiO_2_-supported WO_*x*_ was explained by quantum chemical calculations that reveal the lack of change in the oxidation state of W is attributed to charge delocalization on the surface atoms of the titania support, which does not occur on silica. Moreover, modeling results at <600 K in the presence of H_2_ suggest the formation of Brønsted acid sites, and the absence of Lewis acid sites, on larger aggregates of WO_*x*_ on silica and all cluster sizes on titania. These results provide experimental and theoretical insights into the nature of supported tungsten oxide clusters under conditions relevant to various catalytic reactions.

## 1 Introduction

Supported tungsten oxide catalysts have gained significant attention in the field of heterogeneous catalysis due to their widespread application in various catalytic processes such as dehydrogenation of alcohols, selective catalytic reduction of NO_*x*_, oxidative coupling of methane, isomerization of alkenes and alkanes, and dehydration of alcohols.^1−3^ Supported tungsten oxide catalysts can be promoted with platinum group metals (PGM), which are known to aid in the dissociation of H_2_ into atomic hydrogen.^4^ Spillover of atomic hydrogen can promote the reduction of the supported metal oxide (i.e., WO_*x*_), and is suggested to play a crucial role in enabling the reduction of carboxylic acids to alcohols and aldehydes.^5,6^ During these reactions, H_2_ can participate as a reactant^7^ and (or) be involved in creating the active site(s) on the catalyst.^5,8,9^ The identity and density of these active sites are affected by the nature of the support,^10^ and our goal here is to discern how the WO_*x*_ species transform on chemically diverse supports (i.e., TiO_2_ and SiO_2_) when atomic hydrogen is available from spillover.

Hydrogen spillover from PGMs to WO_*x*_ has been shown to generate actives sites for catalytic reactions involving H_2_ across a variety of supports. Hydrogen spillover from Pt to WO_*x*_ on a Pt–W–TiO_2_ catalyst was shown by Raman spectroscopy to consume the W=O functional group and generate Brønsted acid sites. Reduction of W^6+^ was confirmed with X-ray photoelectron spectroscopy (XPS), which showed an increase in the W^5+^/W^6+^ ratio.^5^ Similarly, SiO_2_-supported Pd–W show reduction of W^6+^ to W^5+^ from X-ray absorption spectroscopy (XAS) under reaction conditions for the reduction of propionic acid to propanol in H_2_.^6^ Inverse Pt–W catalysts, where WO_*x*_ is deposited onto silica-supported Pt, show evidence of W reduction and generation of Brønsted acid sites at 673 K, in contrast to a W–SiO_2_ catalyst that showed a maximum consumption at 1100 K in the profile of temperature-programed reduction (TPR).^8^ Titania-supported Pd–W catalysts are also active for the reduction of propionic acid to propanol in H_2_,^6^ which suggests that reduction of W may also occur on these materials, similar to the reduction of Ti^4+^ to Ti^3+^ observed by XPS for Pd supported on TiO_2_ during exposure to H_2_.^11^

While there is agreement in literature regarding the ability of PGM-promoted tungsten oxides to catalyze a variety of reactions, the nature of the active site(s) is still debated, especially on different supports. Numerous experimental techniques have been used to investigate supported WO_*x*_ catalysts and computational investigations have indicated that formation of Brønsted acid sites on WO_*x*_ catalysts is influenced by reaction conditions.^8,12,13^ The composition of the support can also alter the nature of the active site. For instance, a ZrO_2_ support can increase the Brønsted acidity of larger WO_*x*_ clusters.^14,15^ Similarly, computational investigations have reported that monomeric WO_*x*_ is the preferred stable configuration on a titania support^16,17^ whereas trimers are preferred on a Pt support.^12^ Hence, the support composition and domain size of supported WO_*x*_ catalysts is inextricably linked to its catalytic activity.

Here, we aim to understand the molecular configuration/structure, charge states, as well formation of acid sites on supported tungsten oxide clusters as a function of experimentally relevant reaction conditions such as temperature and H_2_ pressure through experimental and computational approaches. We explore the tungsten oxide speciation and reducibility on two different supports: SiO_2_, a nonreducible support, and TiO_2_, a reducible support. By using Pd to facilitate the generation of atomic hydrogen (in an H_2_ environment) and thus hydrogen chemical potential via the spillover effect on supported tungsten oxide species, we relate our experiments to computational results. We show tungsten oxide cluster sizes vary depending on the support, with TiO_2_-supported WO_*x*_ clusters being much smaller than their SiO_2_ analogs. Furthermore, the W atoms in WO_*x*_ species supported on SiO_2_ are able to reduce from a +6 to a primarily +5 oxidation state, while the fraction of W atoms reduced on the TiO_2_ support was minor, even in the presence of Pd. Density Functional Theory (DFT) calculations for different cluster sizes on the two supports revealed that the charge delocalization on the titania support prevents significant reduction of the supported WO_*x*_ cluster. Moreover, quantum chemical calculations suggest that, at conditions relevant for catalysis, the presence of H_2_ forms Brønsted acid sites on the WO_*x*_ clusters.

## 2 Results and Discussion

### 2.1 Tungsten Oxide Cluster Size on SiO_2_ and TiO_2_ Supports

**Figure 1:**
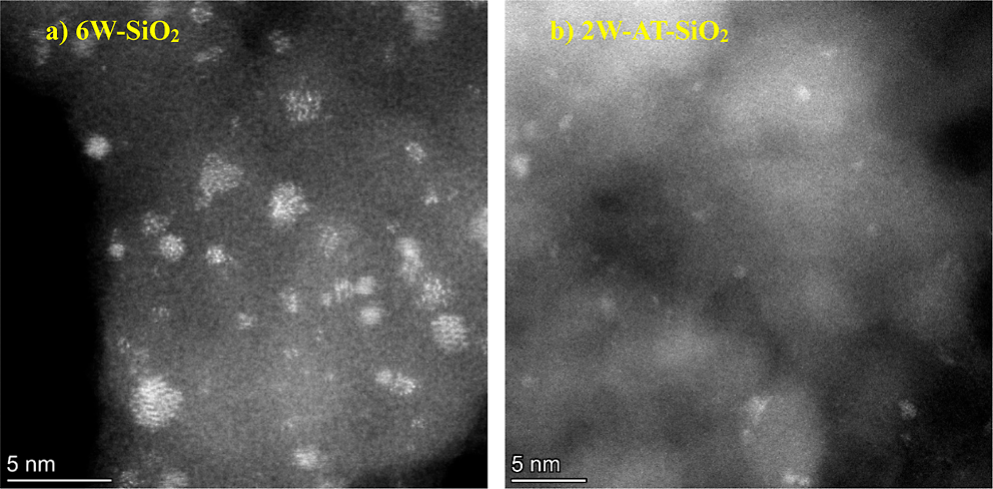
High resolution HAADF-STEM images of (a) 6W–SiO_2_ and (b) 2W-AT-SiO_2_.

**Figure 2:**
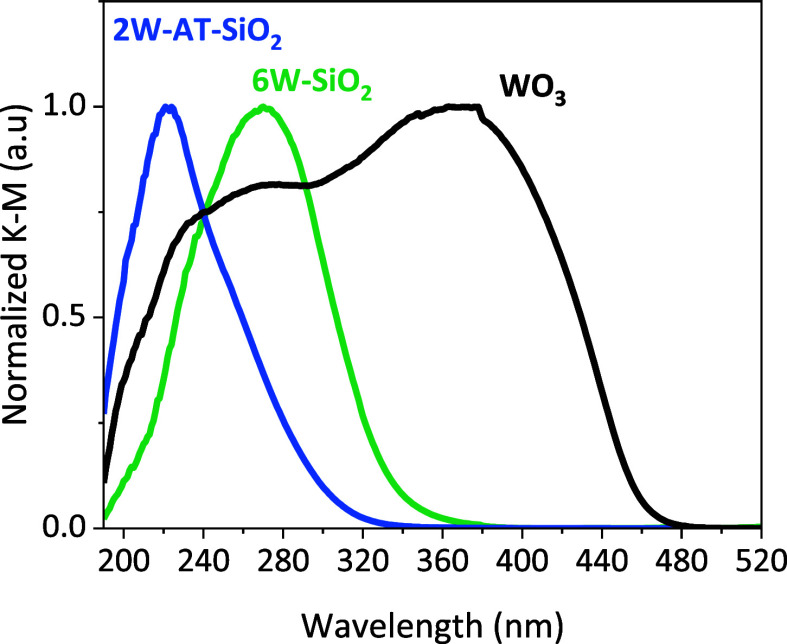
Normalized DR UV–vis spectra of silica-supported W materials and reference WO_3_.

To provide a visual representation of the tungsten oxide cluster sizes on SiO_2_ and TiO_2_ supports we characterized samples with high resolution high-angle annular dark-field scanning transmission electron microscopy (HAADF-STEM) following W deposition and pretreatment in flowing dry air at 923 K. Figures 1 and 2 show HAADF-STEM images of SiO_2_- and TiO_2_-supported W samples, respectively (samples are labeled as (Pd)-xW-SiO_2_ where x is the nominal weight percent of W). Figure 1a shows WO_*x*_ nanoparticles and small clusters on the 6W–SiO_2_ sample, which are primarily in the range of 1 to 3 nm in diameter. The acid-treated silica sample 2W-AT-SiO_2_ shown in Figure 1b reveals both a lower number density as well as smaller average WO_*x*_ size relative to 6W–SiO_2_. The additional STEM image of a lower W loading on SiO_2_ (3W–SiO_2_) area provided in the Supporting Information (Figure S1) also shows cluster sizes in the 1–3 nm range. Furthermore, no crystalline phases were detected by X-ray diffraction (XRD) of silica-supported samples, suggesting that very large WO_*x*_ aggregates are not present in these materials. Electron microscopy with elemental mapping of the Pd-promoted 6W–SiO_2_ sample, provided in Figure S1c, show that incorporation of Pd nanoparticles of about 5 nm did not reorganize the dispersed WO_*x*_ clusters.

To supplement the characterization of tungsten oxide cluster sizes derived from the HAADF-STEM images for the SiO_2_ supported samples, we used diffuse reflectance (DR) UV–vis spectroscopy (for TiO_2_-supported W, background adsorption of the TiO_2_ support precludes similar analysis, Figure S2). Figure 2 shows UV–vis spectra for the reference bulk WO_3_ standard and SiO_2_-supported W samples, with Ligand-to-Metal-Charge-Transfer (LMCT) band absorption maxima and direct optical bandgaps for corresponding samples tabulated in Table S1, with an example Tauc plot^18^ shown in Figure S3. The SiO_2_-supported tungsten oxide samples show a distribution of LMCT bands and direct bandgaps. The 2W-AT-SiO_2_ sample has an LMCT band at 221 nm, with a corresponding bandgap of 4.8 eV, consistent with fairly isolated monomeric WO_*x*_ species^19,20^ such as those in Na_2_WO_4_ which has a bandgap of 5.1 eV (Figure S4 and Table S1). However, the broad tail of the band suggests the presence of additional larger oxide clusters, consistent with the small clusters observed by HAADF-STEM in Figure 1b. The 6W–SiO_2_ sample shows a higher wavelength absorption band at 270 nm with a direct bandgap of 4.0 eV, with the lower loaded 3W–SiO_2_ also showing a similar band at 261 nm and bandgap of 4.1 eV in Figure S4, which suggests the presence of WO_*x*_ nanoparticles with some potentially distorted yet isolated sites on the SiO_2_ support,^21^ aligning with the HAADF-STEM images in Figures 1 and S1. For comparison, the reference WO_3_ bulk standard shows multiple features in the spectra with the main band at 378 nm corresponding to a direct bandgap of 2.8 eV. The DR UV–vis results of SiO_2_-supported W samples arise from the well-known quantum size effect of semiconductor oxides. High bandgaps, such as those of 2W-AT-SiO_2_, are representative of smaller oxide cluster sizes, while lower bandgaps, such as those of 6W–SiO_2_ and WO_3_, are representative of larger clusters and bulk oxide species, respectively.

**Figure 3:**
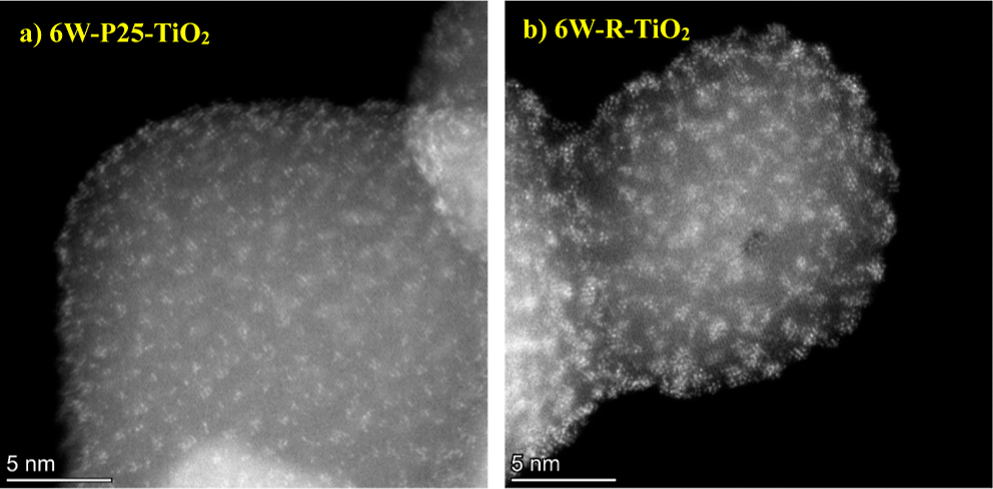
High resolution HAADF-STEM images of (a) 6W–P25–TiO_2_ and (b) 6W-R-TiO_2_.

Unlike the WO_*x*_ clusters supported on SiO_2_ in Figure 1, TiO_2_-supported WO_*x*_ species are subnanometer in size regardless of the titania crystal phase (Figure 3a shows the P25–TiO_2_ support, which is primarily anatase, while Figure 3b shows the rutile, R, TiO_2_ support). The WO_*x*_ species are highly dispersed on both TiO_2_ supports, with predominately low W nuclearity (e.g., W monomers, dimers, trimers, etc.) WO_*x*_ clusters present. Additional images with higher resolution and lower W loading supported on P25–TiO_2_ are provided in Figures S5a and S5b, respectively. Electron microscopy with elemental mapping of the Pd-promoted 6W–TiO_2_ sample, provided in Figure S5c, show that incorporation of Pd nanoparticles did not reorganize the highly dispersed WO_*x*_ clusters. Results from microscopy indicate WO_*x*_ forms larger W domains on the untreated SiO_2_ support than on either TiO_2_ support, consistent with results from ab initio thermodynamic modeling at synthesis conditions described below (Sections 2.3.1 and 2.3.2).

### 2.2 Reducibility of Tungsten Oxide on SiO_2_ and TiO_2_ Supports

#### 2.2.1 Dihydrogen-TPR

**Figure 4:**
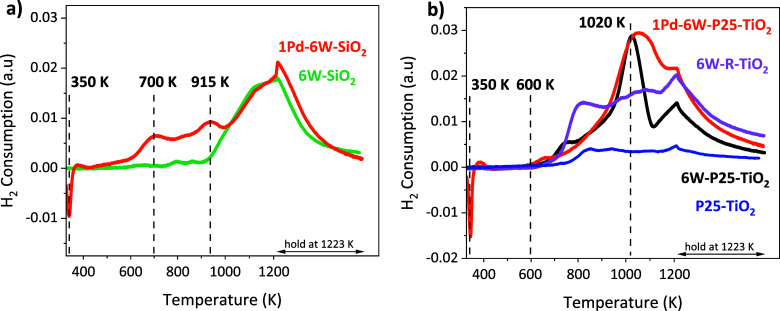
TPR profiles of (a) 1Pd–6W–SiO_2_ and 6W–SiO_2_ samples and (b) 1Pd–6W–P25–TiO_2_, 6W–P25–TiO_2_, and 6W-R-TiO_2_ samples with a ramp rate of 10 K min^–1^ to 1223 K and hold for 20 min under a flow of 5% H_2_/Ar at 30 cm^3^ min^–1^.

We used dihydrogen TPR to quantify the reducibility of SiO_2_- and TiO_2_-supported WO_*x*_ species and the effect of hydrogen spillover from Pd. Figure 4a shows the TPR profiles of 6W–SiO_2_ and 1Pd–6W–SiO_2_. Reduction of the WO_*x*_ species on 6W–SiO_2_ begins at 915 K and continues to 1223 K. The 1Pd–6W–SiO_2_ sample and the 1Pd–6W–P25–TiO_2_ sample (Figure 4b), show an inverse peak at 350 K associated with the decomposition of the β-phase Pd hydride.^22^ Initial reduction of WO_*x*_ species on SiO_2_ begins around 450 K, with two peaks at 700 and 915 K attributed to the spillover of atomic hydrogen from Pd (Figure 4a). Our results for WO_*x*_ on silica are consistent with PGMs decreasing the initial reduction temperature of reducible metal oxides, which has been shown for a variety of comparable systems (vide supra), such as Pd-promoted MoO_3_ catalysts.^23^ Further reduction of tungsten oxide species continues to 1223 K, similar to the 6W–SiO_2_ sample without Pd.

**Figure 5:**
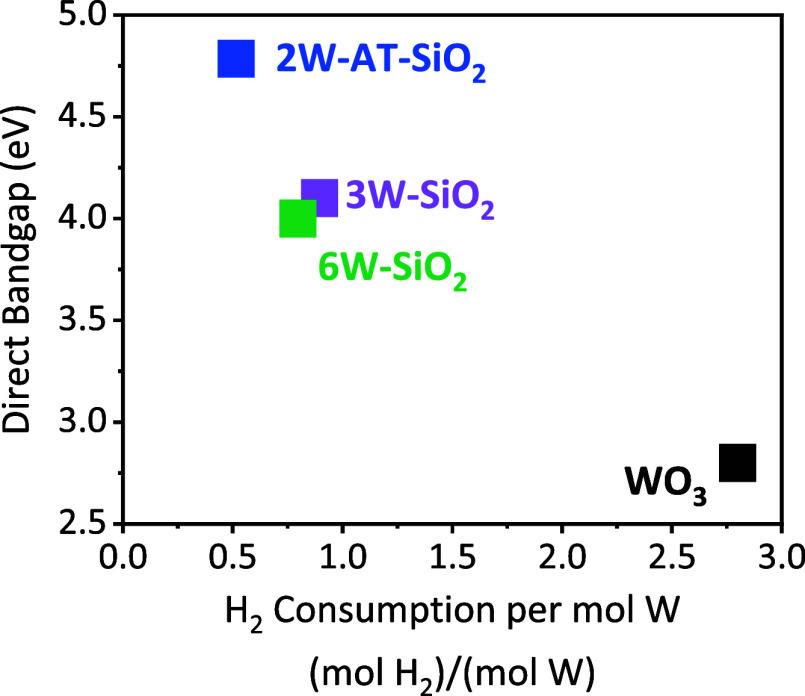
Correlation of H_2_ consumption per mol W, calculated from H_2_-TPR results, versus the direct bandgap of WO_*x*_ species, calculated from DR UV–vis.

To probe the relationship between cluster size and reducibility of the SiO_2_-supported WO_*x*_ species, we compared the experimentally measured direct bandgap associated with the tungsten oxide clusters, which is inversely related to cluster size, to the hydrogen consumption during TPR up to 1223 K (Table S2, Figures 5, S7, and S8). The 2W-AT-SiO_2_ sample showed the highest bandgap, which had the smallest oxide cluster size and the lowest H_2_ consumption per W, consistent with its high stability. Lower band gap materials (larger cluster sizes) consumed more H_2_ during TPR, and the bulk WO_2_ (Figure S9) and WO_3_ samples consumed the most H_2_, equivalent to nearly complete reduction to metal. Conversely, the WO_*x*_ species on the SiO_2_ supported samples *do not* reduce to W metal under the conditions of the TPR, indicating that SiO_2_-supported tungsten oxides reduce to intermediate oxidation states between +6 and 0. This result shows that larger tungsten oxide clusters consume more H_2_, and are thus more likely to reduce, in agreement with modeling results discussed in Section 2.3.1. The overlap of the TiO_2_ absorption band with the WO_*x*_ band during DR UV–vis (as previously discussed in Section 2.1) combined with the background reduction of the TiO_2_ support during H_2_-TPR prevented a similar analysis for TiO_2_-supported WO_*x*_ species.

In contrast to SiO_2_, the addition of Pd *does not* appear to promote reduction of TiO_2_-supported WO_*x*_ until temperatures >1000 K. Figure 4b shows the TPR profiles of 6W-R-TiO_2_, 6W–P25–TiO_2_, 1Pd–6W–P25–TiO_2_, and the bare P25–TiO_2_ support. All three tungsten containing samples show an initial reduction at 600 K, which is at lower T than that of the SiO_2_-supported samples. However, this is the same temperature where the P25 and R–TiO_2_ supports also begin to consume H_2_ (Figures 4b and S6). The hydrogen consumption associated with the reducible titania support prevents determination of the exact hydrogen uptake by the WO_*x*_ species, however it is evident that the addition of WO_*x*_ species on both TiO_2_ supports allows for a higher consumption of H_2_ during TPR than the bare support.

#### 2.2.2 XPS

**Figure 6:**
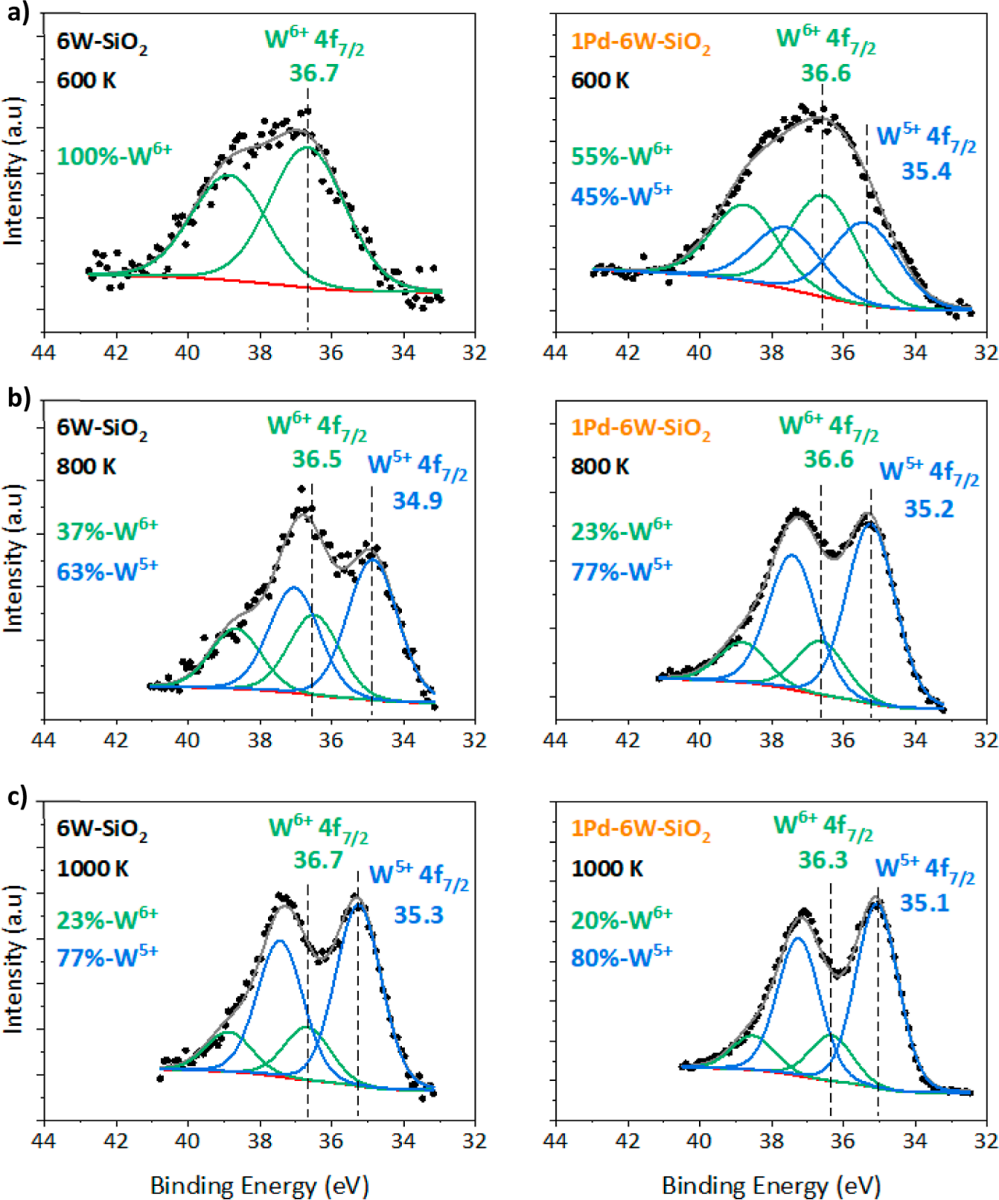
Photoemission spectra and peak fits of the W 4f region for 6W–SiO_2_ and 1Pd–6W–SiO_2_ samples following a treatment in 5% H_2_/N_2_ at 30 cm^3^ min^–1^ at (a) 600 K, (b) 800 K and (c) 1000 K. Percentages of W^6+^ and W^5+^ were calculated based on the area of their respective W 4f_7/2_ peaks. Spectra were charge referenced to the Si 2p peak at 103.5 eV.

To determine the resulting oxidation states of supported tungsten oxide species following various thermal treatments in H_2_ we used XPS. Tungsten oxides exist in various stoichiometries, with WO_3_, in which W is in a +6 oxidation state, being the most common. Removal of some oxygen from WO_3_ results in WO_3-*x*,_ which is a nonstoichiometric oxide with a W-oxidation state between +6 and +4, commonly denoted as +5.^24−26^ Further removal of oxygen forms WO_2_, with a +4 oxidation state, followed by W^0^ metal.^24,26^Figure 6a–c show photoemission spectra for the W 4f region of 6W–SiO_2_ and 1Pd–6W–SiO_2_ samples following a reducing treatment in 5% H_2_/N_2_ at 600, 800, and 1000 K, respectively, with peak fitting parameters tabulated in Section S.1.1. The W 4f_7/2_ peak positions corresponding to specific oxidation states, together with areas of each species derived from curve fitting, were used to assign sample oxidation states following these treatments. After treatment at 600 K, the spectrum of 6W–SiO_2_ shows a W 4f_7/2_ peak at 36.7 eV, which is consistent with a W oxidation state of +6 in WO_3_,^26^ suggesting all the tungsten oxide species are initially in a +6 oxidation state. After the same reducing treatment, 1Pd–6W–SiO_2_ shows two W 4f_7/2_ peaks at 36.6 and 35.4 eV,^26^ corresponding to 55% of W^6+^ and 45% of W^5+^. Additionally, Figure S10 shows the Pd was completely reduced to Pd^0^ by 600 K in H_2_. Further treatment at 800 K on the 6W–SiO_2_ sample shows reduction of the W species with 37% W^6+^ and 63% W^5+^, while 1Pd–6W–SiO_2_ shows a distribution of 23% W^6+^ and 77% W^5+^ as shown in Figure 6b. Upon an H_2_ treatment at 1000 K, both samples still show W4 f_7/2_ peaks that are attributed to W^6+^ and W^5+^ species with majority of species in the W^5+^ state for both samples, 77% for 6W–SiO_2_ and 80% for 1Pd–6W–SiO_2_, as shown in Figure 6c. The difference between the W 4f_7/2_ peak positions of W^6+^ and W^5+^ species is 1.4 and 1.2 eV for 6W–SiO_2_ and 1Pd–6W–SiO_2_, respectively. As the W4 f_7/2_ position of W^4+^ is expected to be 2.8–3.0 eV lower than that of W^6+^,^26,27^ we found no evidence for W^4+^ in our samples even after treatment in H_2_ up to 1000 K. Instead, the final oxidation state of W on these samples after reduction treatment is primarily +5, with some species still in the +6 oxidation state.

Comparable systems, such as unsupported Pd–MoO_3_ catalysts, show that Mo in unpromoted molybdenum oxide resides in a primarily +6 oxidation state, even under reducing conditions up to 673 K, while the addition of Pd allows for reduction to +5 and +4 Mo species.^23^ While our SiO_2_-supported tungsten species exist in a +6 and +5 oxidation state, similar to what has been previously reported for unsupported Pd-WO_*x*_ catalysts,^28^ the addition of Pd helps facilitate the reduction of the tungsten oxide to a higher fraction of +5 species, especially at temperatures at or below 800 K.

**Figure 7:**
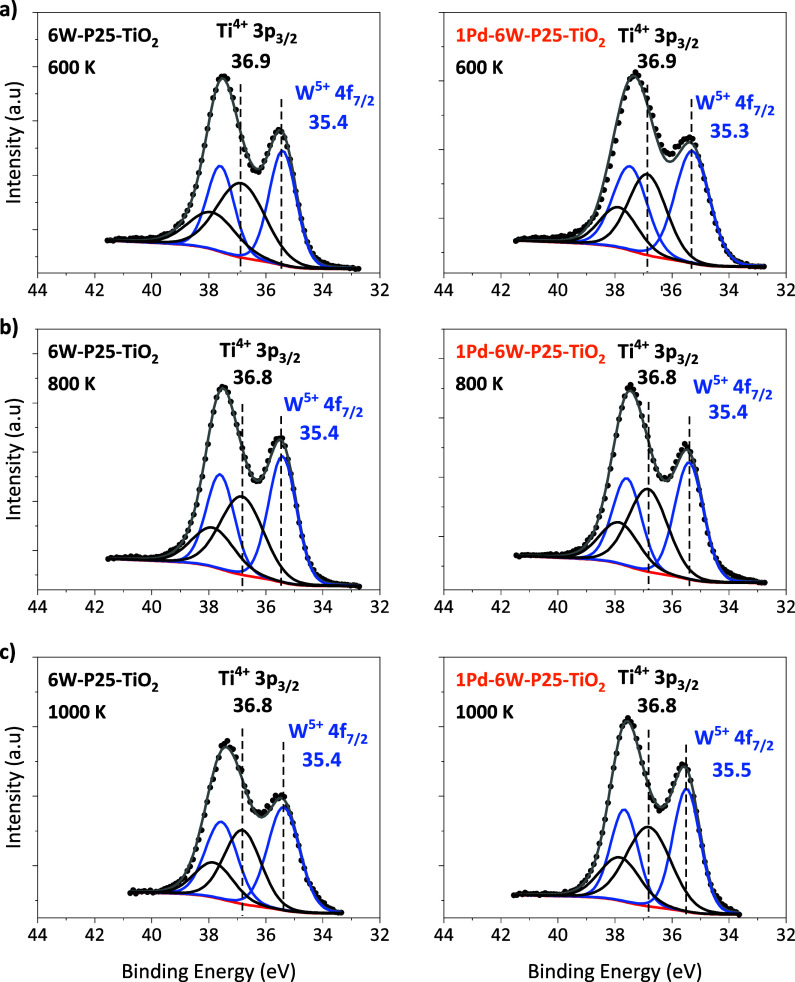
Photoemission spectra and peak fits of the W 4f (and Ti 3p) region for 6W–P25–TiO_2_ and 1Pd–6W–P25–TiO_2_ samples following a treatment in 5% H_2_/N_2_ at 30 cm^3^ min^–1^ at (a) 600 K, (b) 800 K, and (c) 1000 K. Spectra were charge referenced to the Ti 2p_3/2_ peak at 458.7 eV.

To elucidate the oxidation state changes of WO_*x*_ species supported on P25–TiO_2_ the same procedure was followed. The spectra were charge referenced to the Ti 2p_3/2_ peak at 458.7 eV as that peak showed no change in shape, i.e., broadening, Figure S11, which suggests most of the Ti cations in the support analyzed by XPS remained as Ti^4+^ following thermal treatments in H_2_. Thus, the Ti 3p peaks of the titania support were assumed to be constant during fitting of WO_*x*_ peaks (based on fitting of the bare P25–TiO_2_ support after the same reducing treatment as depicted in Figure S12). Consistent with SiO_2_-supported samples, Pd was completely reduced to Pd^0^ after heating to 600 K in H_2_ (Figure S14). Figure 7a-c show the W 4f (and Ti 3p) region of 6W–P25–TiO_2_ and 1Pd–6W–TiO_2_ samples following reducing treatments in 5% H_2_/N_2_ at 600, 800, and 1000 K, respectively. Some tungsten in a +5 oxidation state was observed following reducing treatments at those temperatures on both samples, as well as the as-synthesized (nonreduced) sample (Figure S13) but overlap of the Ti 3p_3/2_ peak from the titania support prevented quantification of W in the +6 oxidation state relative to +5. Moreover, the broad nature of W 4d peaks also prevent quantification on our samples. Prior reports on Pt–W–TiO_2_ catalysts have indicated that W exists primarily as +6, and that following H_2_ reduction can further reduce to +5, while maintaining a larger fraction of the +6 oxidation state, albeit at higher H_2_ partial pressures (>0.1 MPa).^5^ Importantly, the fraction of tungsten +5 determined by XPS of our titania-supported samples does not change significantly during reducing treatments up to 1000 K regardless of the presence of Pd, which is in stark contrast to the SiO_2_-supported species.

#### 2.2.3 In Situ XAS

**Figure 8:**
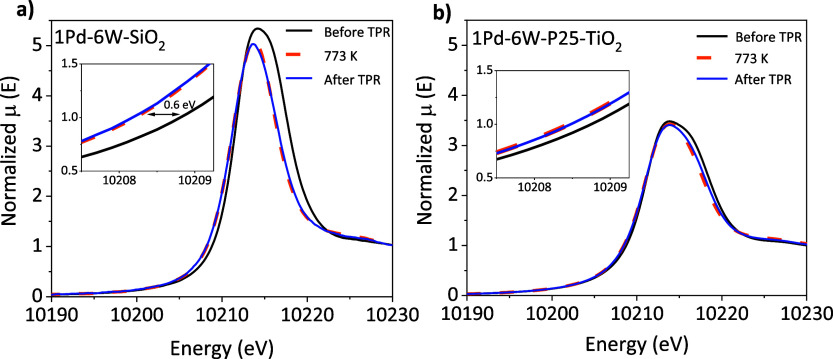
In situ XANES spectra of the W L_III_ edge before and after a TPR at 773 K under a flow of 5% H_2_/N_2_ at 20 cm^3^ min^–1^ of (a) 1Pd–6W–SiO_2_ and (b) 1Pd–6W–P25–TiO_2_ with insets shown for clarity. Note: 1Pd–6W–SiO_2_ data were collected in transmission mode and 1Pd–6W–P25–TiO_2_ data were collected in fluorescence mode. See Section 4.2 for more detail.

To further explore the apparent difference in WO_*x*_ reducibility on the two supports, samples were monitored by XAS throughout a H_2_-TPR experiment. Temperature limitations of the cell design limited the maximum temperature during TPR to 773 K, which is lower than that in the XPS experiments. Figure 8 shows a comparison of the X-ray absorption near-edge structure (XANES) at the L_III_ edge of W before TPR, at 773 K, and after TPR. Figure 8a shows the edge energy for the 1Pd–6W–SiO_2_ sample shifts from 10208.8 to 10208.2 eV, consistent with a change in oxidation state from W^6+^ to a lower oxidation state that is not reduced all the way to W^4+^ observed for WO_2_ (Figure S15). Upon cooling down the sample to room temperature in dihydrogen, the edge remained shifted, which indicates the sample remained reduced. Figure 8b shows the same TPR experiment on 1Pd–6W–P25–TiO_2_, which had a small edge shift from 10208.7 to 10208.5 eV, and 10208.6 eV at TPR conditions and upon cooling, respectively. The small change in the edge position (0.2 eV) during TPR indicates the tungsten oxidation state remained the same throughout the experiment, in agreement with the XPS results.

Figures S16 and S17 show the analogous two samples without Pd, 6W–SiO_2_ and 6W–P25–TiO_2_, did not incur a significant shift in the edge position during TPR (∼0.2 eV). Indeed, for 6W–SiO_2_, there is very little reduction of WO_*x*_ below 773 K as illustrated by the TPR profiles in Figure 4a. The edge position of the Pd-free samples corresponded to a W-oxidation state of around +6 for tungsten oxide on SiO_2_ and TiO_2_, respectively.

The results from the in situ XANES of the SiO_2_-supported samples agree with the XPS results, in which Pd aids in reducing the tungsten oxide species from majority +6 to mostly +5 species. However, while the XAS results suggest an oxidation state of near +6 for all the TiO_2_-supported samples regardless of reduction temperature and presence of Pd, the XPS shows the presence of some W in a +5 state (Figure 7). This is likely due to the difficulty of XPS peak fitting, stemming from the overlap of the Ti 3p regions of the P25–TiO_2_ support on both 1Pd–6W–P25–TiO_2_ and 6W–P25–TiO_2_. Regardless, the observed trends from in situ XANES and XPS show significant changes in W-oxidation state during TPR of 1Pd–6W–SiO_2_ but negligible changes in W-oxidation state during TPR of 1Pd–6W–P25–TiO_2_. Thus, even though both SiO_2_ and TiO_2_ supported WO_*x*_ materials consume more H_2_ during TPR than their bare supports (Figure S6), the fact that W reduces when supported on SiO_2_ and remains in the same oxidation state on TiO_2_ is intriguing. Therefore, we used molecular modeling to rationalize these disparate outcomes for the two supports.

### 2.3 Computational Modeling of Supported WO_*x*_ Clusters

To investigate the differences in W reducibility observed in the experiments, we used DFT calculations (full details in Section 4.3) to model the molecular and electronic structures of variable stoichiometry tungsten oxide clusters on the two supports. Results for WO_*x*_ clusters supported on TiO_2_ are reported in Section 2.3.2, and we begin here with SiO_2_.

#### 2.3.1 SiO_2_-Supported WO_*x*_

Previous computational reports indicate that the WO_*x*_ species on silica depend on both the type of surface model and choice of grafting sites in those models.^29,30^ We used the β-crystabolite-SiO_2_ (001) surface as a surrogate model for amorphous silica since it preserves its electronic properties while avoiding the configurational sampling issues inherent to amorphous supports (additional discussion and validation provided in Section S.2.1). Notably, the (001) surface displays a density and refractive index similar to amorphous silica and can be hydroxylated to generate a silanol density comparable to amorphous silica.^31−33^Figure 1 shows that on silica surfaces tungsten oxide formed 1–3 nm clusters, with some subnanometer clusters present on AT-SiO_2_. However, molecular modeling of nanometer-sized tungsten oxide clusters on silica introduces extreme configurational complexity challenges, thus we limit our scope to WO_*x*_ monomers, dimers, and trimers that can be investigated systematically. We include discussion for the reduction of bulk WO_3_ (Section S.2.2) to address the behavior of larger silica-supported particles. While the models used here are not truly representative of the synthesized silica-supported WO_*x*_ clusters, they provide a useful qualitative approximation for ascertaining trends in the reducibility of WO_*x*_ as a function of cluster size, in correspondence with Figure 5. Grafting these clusters on the SiO_2_ surface (terminated with silanol groups) requires the removal of at least one surface H atom, enabling W to bind with one or more undercoordinated surface O atoms. We generated configurations removing between 1 and 4H atoms from the top of the SiO_2_ slab and attaching W to the surface O atom(s). Details of the structure generation and the different configurations considered are described in Section S.2.3.

**Figure 9:**
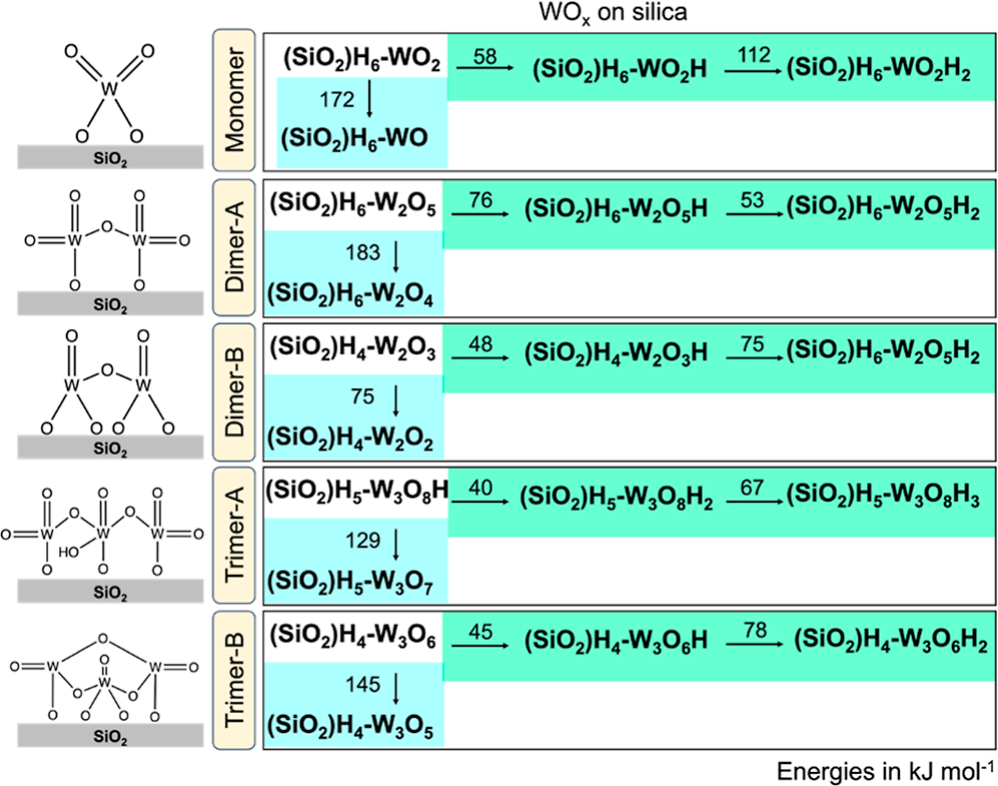
Reaction energies for forming different silica-supported WO_*x*_ clusters. Green shaded structures were generated from H-addition, and blue shaded structures from O-removal. Schematic representations for the starting structures are shown on the left. Molecular structure files are provided as Supporting Information.

Comparing the free energy of these structures under conditions relevant to catalyst synthesis across different temperatures (Figure S29a, 0.01 kPa H_2_O, 20 kPa O_2_, 300 to 1200 K, since catalyst synthesis involves thermal treatment at 923 K), WO_*x*_ trimers are more stable than WO_*x*_ monomers and dimers from 300 to 1000 K. While this result is consistent with the higher population of larger WO_*x*_ aggregates relative to highly dispersed WO_*x*_ observed in Figure 1, the larger WO_*x*_ aggregates are more likely to be similar to bulk WO_3_ (discussed below). To explore how different WO_*x*_ clusters evolve under exposure to H_2_, we started with the lowest free energy structures under synthesis conditions at 923 K, detailed in Section S.2.3. For the dimer and trimer configurations, there are two structures within 50 kJ mol^–1^ at 923 K, so we considered both as starting structures. Figure 9 reports the starting structure (upper left of each panel) for different WO_*x*_ domain sizes using the nomenclature (SiO_2_)H_*a*_ – W_*b*_O_*c*_. For example, in the WO_*x*_ monomer species (SiO_2_)H_6_–WO_2_, (SiO_2_)H_6_ indicates 6 remaining H atoms on the silica surface (with 2H atoms removed for monomer grafting), and in this example WO_2_ signifies the addition of 1 W and 2 O atoms to establish the initial structure for the WO_*x*_ monomer.

Palladium-catalyzed dissociation of H_2_ to 2H creates a reservoir of H atoms that can transport via spillover and may react with tungsten oxide clusters. Figure 9 shows the two different reactions for reduced WO_*x*_ species of each cluster size that we considered – H attached to an O atom in the grafted tungsten oxide cluster (forming a Brønsted acid site, green shading) and dehydration with tungsten oxide forming an open coordination site (a Lewis acid site, blue shading). Both types of acid sites have been reported by various titration experiments^8,15,34^ on supported WO_*x*_ species. We did not consider H_2_O adsorption to WO_*x*_ because open coordination sites only form at extreme temperatures (vide infra) and the XPS conditions were 600–1000 K. We explored a cascade of adding H and removing O atoms, including all intermediate combinations. The reaction cascade was terminated when: consecutive H addition energies were endothermic, or the O removal energy was greater than 70 kJ mol^–1^ (much larger than the free energy offset for consumption of H_2_ and release of H_2_O). We considered all possibilities (removal of each O, and H addition to each possible O) for each reaction. Figure 9 reports the lowest energy pathway for these reactions and shows that both H-addition and O-removal are endothermic for all structures. These results suggest that H-addition to the WO_*x*_ clusters is prohibitive because it is both enthalpically and entropically unfavorable, with the exception of trimer A, which includes bound H as a low free energy starting structure. Comparatively, the reaction energies in Figure S21 demonstrate that reducing bulk WO_3_ to WO_2_ and W metal, or adding one H atom to bulk WO_3_, is energetically more favorable, suggesting that larger nanometer-sized clusters would be easier to reduce.

**Figure 10:**
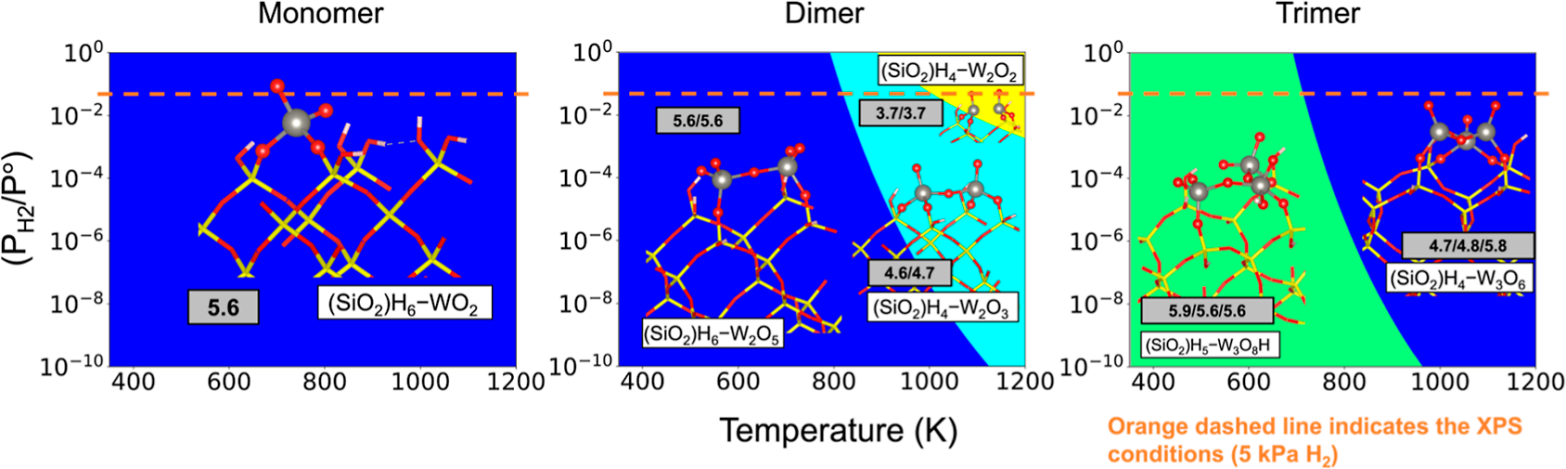
Ab initio thermodynamic phase diagrams for silica supported WO_*x*_ monomer, dimer, and trimer at *P*_H2O_ = 0.01 kPa, where *P*° is the reference pressure of 101.3 kPa. Gray boxes report the oxidation state of W from Bader charge analysis. Diagrams were generated using the HSE06 functional.

Assuming the dissociation of dihydrogen on Pd and subsequent spillover to WO_*x*_ is in quasi-equilibrium with the gas phase (i.e., the “best case” scenario without kinetic limitations), Figure 10 reports T-P_H2_ phase diagrams generated using the free energies computed for the library of structures generated in Figure 9. Figure 10 shows that only one tungsten oxide monomer species, (SiO_2_)H_6_–WO_2_, is lowest in free energy across a wide range of conditions among all monomer structures considered. The (SiO_2_)H_6_–WO_2_ structure is 4-fold coordinated to oxygen in a tetrahedral configuration, similar to tetrahedral and distorted tetrahedral configurations reported for other oxide supported tungsten monomers.^16,19,35−37^ Oxygen removal from the WO_*x*_ monomer results in a trigonal planar configuration, which is not a stable coordination environment for W.^38^ As the WO_*x*_ domain size increases, a broader array of tetrahedral coordination options becomes available for the tungsten oxide clusters, and it remains more thermodynamically favorable to form larger WO_*x*_ clusters rather than isolated monomers under exposure to H_2_ (Figure S29b). The phase diagram for bulk WO_3_ (Figure S22) reveals that upon exposure to H_2_ at different temperatures, WO_3_ either binds hydrogen (reducing the nearby W atom), or is reduced to WO_2_ and W metal.

Figure 10 shows normalized Bader charge analyses for structures in the phase diagrams (gray boxes), revealing a reduction in W-oxidation state with increasing temperature for WO_*x*_ dimers and trimers caused by entropically driven O-removal via dehydration (Figure 9, light blue). Consistent with the Bader charge analyses, integration of the HSE06 computed W projected density of states (DOS) (Section S.2.5) shows a significant increase (≳ 1 e^–^) in the number of occupied states (total e^–^) for the WO_*x*_ dimer and trimer species that form at high *T* and P_H2_ relative to the species that form at low *T*. The reduction of the WO_*x*_ species in the larger tungsten oxide domain sizes is consistent with the XPS results shown in Figure 6, which showed an increase in the amount of W^5+^ species with increasing temperature. Concordant with these results, Figure 5 showed that larger WO_*x*_ aggregates consume more H_2_ per W, and in the limit of bulk WO_3_ reduce to W metal. Previous reports have indicated that WO_*x*_ is less likely to form oligomers on the amorphous silica surface and more likely to form either isolated monomers or larger nanoparticles.^20,21^ Although our results discuss dimers and trimers supported on β-crystabolite-SiO_2_ (001), the larger nanoparticle WO_*x*_ domains are likely to more closely resemble bulk WO_3_, which is easier to reduce than the silica-supported oligomers (Figure S22). The STEM images show WO_*x*_ clusters much larger than trimers (nanoparticle size), and based on the trends from our model clusters and the bulk oxide reduction, we expect that as cluster size increases, SiO_2_-supported WO_*x*_ becomes easier to reduce.

#### 2.3.2 TiO_2_-Supported WO_*x*_

To compare differences in WO_*x*_ reducibility between a nonreducible support (SiO_2_) and a reducible support (TiO_2_) we used a similar workflow to Section 2.3.1 for the TiO_2_-supported WO_*x*_ clusters. Titania exists in three phases: anatase, rutile and brookite.^39^ We used anatase (space group: *I*4_1_/*amd*) and rutile (space group: *P*4_2_/*mnm*) because the P-25 TiO_2_ support used to synthesize the samples is a mixture of both rutile and anatase TiO_2_ (Section S.1.3). We used the most stable surface of each polymorph, which is the (110) surface of rutile^40^ and the (101) surface of anatase.^41^ Similar to the silica-supported materials, we generated initial WO_*x*_ clusters (monomers, dimers, and trimers) where all W atoms have formal oxidation states of +5 or +6,^17^ and used basin-hopping optimization (Section 4.4) to find the lowest energy configurations. These structures were then used for the H addition and O removal reactions, analogous to the workflow for SiO_2_. Figure S33a shows that under synthesis conditions (923 K, 0.01 kPa H_2_O, 20 kPa O_2_), the lowest free energy species for the anatase-supported WO_*x*_ clusters are monomers, whereas dimers are preferred on rutile. The preference to form monomers and dimers on titania, rather than larger aggregates (i.e., trimers), is consistent with the highly dispersed WO_*x*_ clusters from the STEM images (Figure 3), and contrasts with SiO_2_ where larger WO_*x*_ aggregates are thermodynamically favorable at the same conditions.

**Figure 11:**
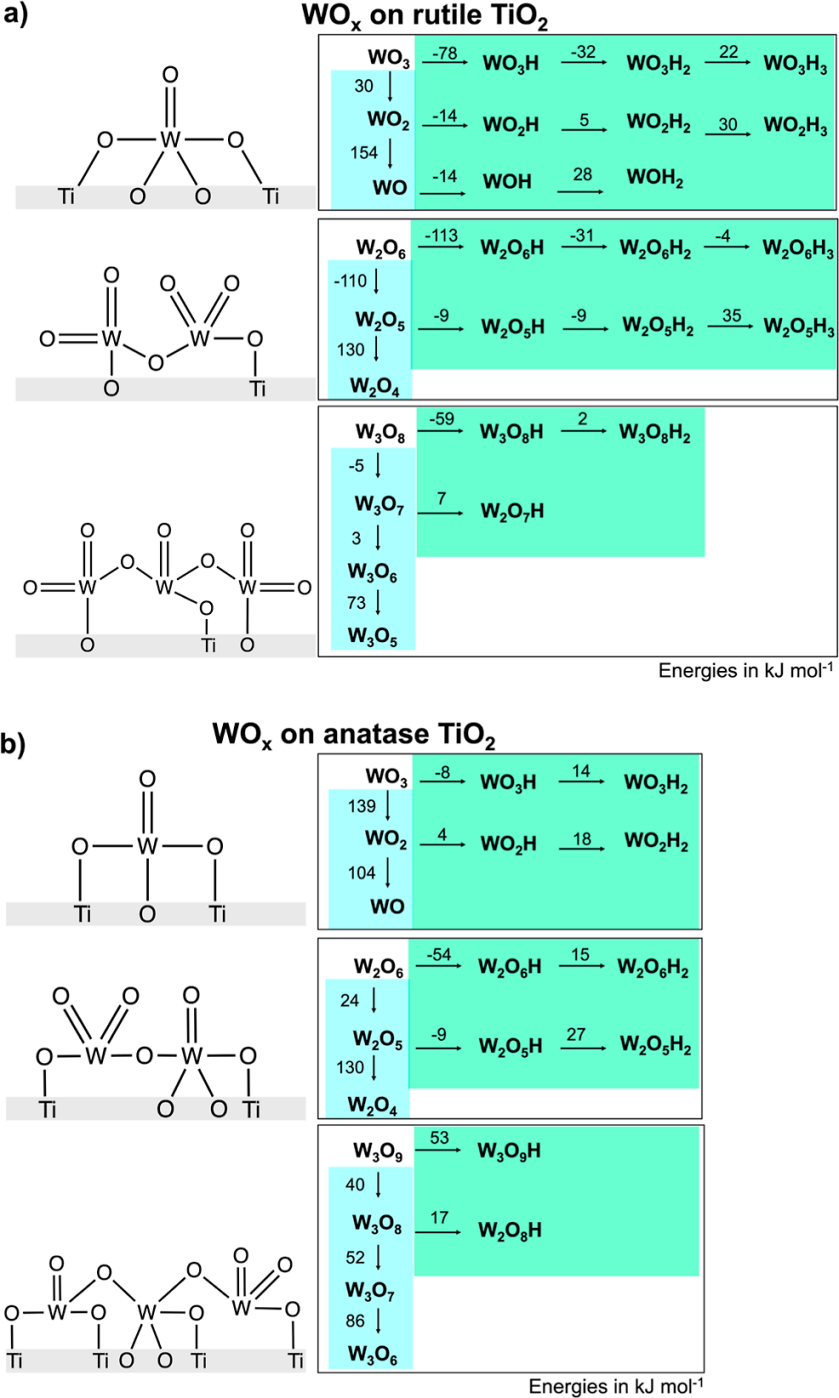
Reaction energies for different WO_*x*_ clusters supported on (a) rutile and (b) anatase TiO_2_.

Figure 11 reports the most exothermic reaction energies (considering all possible configurations) for the cascade of adding H and removing O atoms. Analogous to the procedure used with SiO_2_, the reaction cascade was terminated when: consecutive H addition energies were endothermic, or O removal energies were greater than +70 kJ mol^–1^. The reaction energy for H addition to most of the WO_*x*_ clusters supported on both anatase and rutile titania is exothermic, in contrast to H addition for the SiO_2_ support (Figure 8), suggesting that addition of H to the WO_*x*_ cluster is more favorable when the support is reducible. Several studies show that within the range of conditions considered in our thermodynamic analyses, there may be oxygen vacancies on the titania surface.^42−48^ We explored models that include TiO_2_ O-vacancies near to the WO_*x*_ clusters (Section S.2.7) and found this had minimal impact on the relative free energy of different WO_*x*_ species.

**Figure 12:**
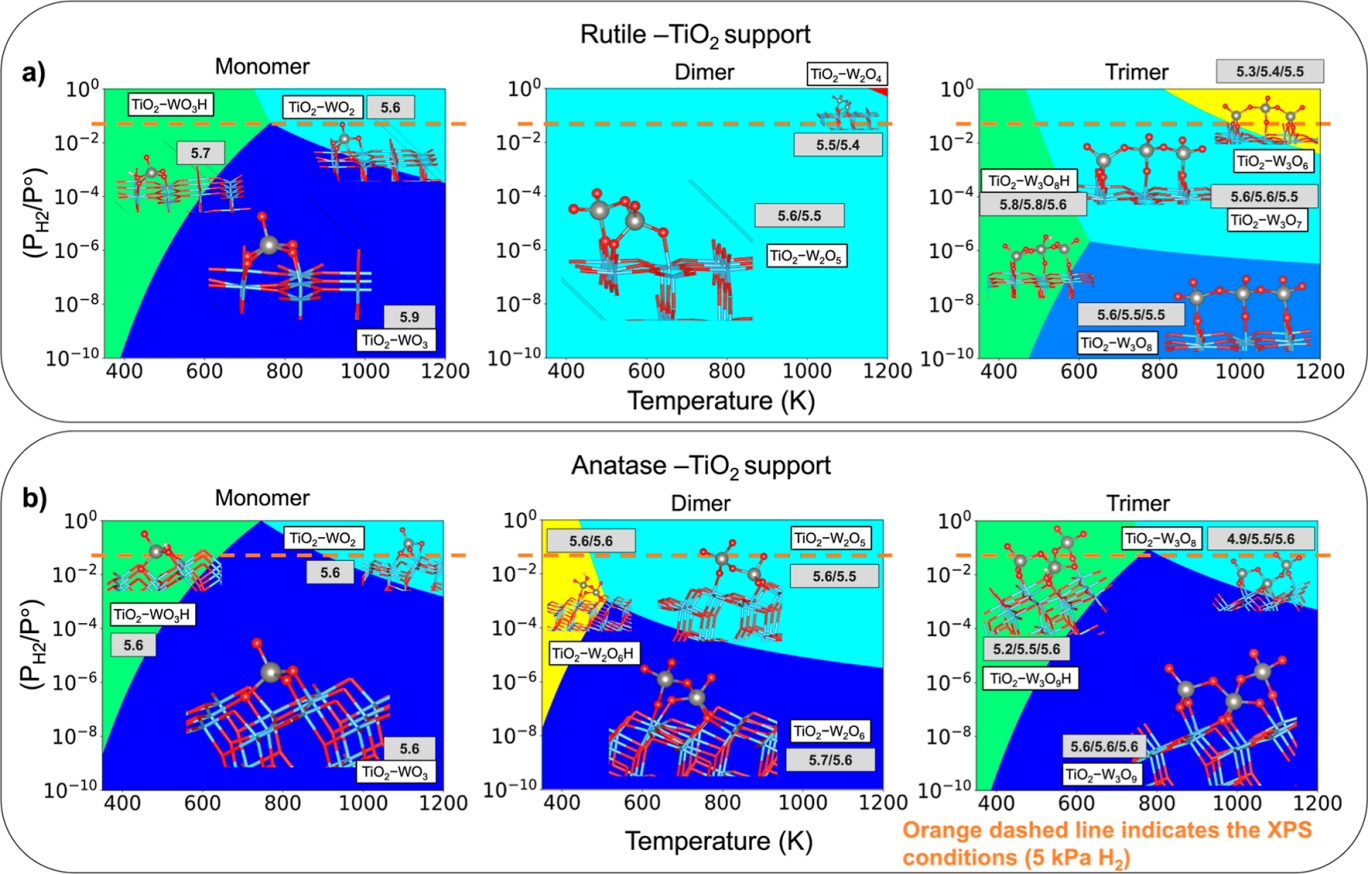
(a) Ab initio thermodynamic phase diagram for rutile TiO_2_-supported WO_*x*_ monomer, dimer, and trimer. (b) Ab initio thermodynamic phase diagram for anatase TiO_2_-supported WO_*x*_ monomer, dimer, and trimer. Gray boxes report the oxidation state of W from Bader charge analysis. Diagrams were generated using the HSE06 functional.

Figure 12 reports the T-P_H2_ phase diagrams constructed using the library of structures from Figure 11. The speciation of WO_*x*_ clusters changes with the conditions and depends on cluster size. At higher temperatures and pressures, oxygen removal becomes favorable for all TiO_2_-supported WO_*x*_ cluster sizes, however, the clusters reconfigure to maintain 4 (tetrahedral) or 5 (square pyramidal) bonds to O during basin-hopping optimization, resulting in no open coordination sites on the W. Most WO_*x*_ species on the phase diagram have a tetrahedral configuration, consistent with the most stable configurations on silica and highly dispersed WO_*x*_ on alumina, titania and ceria supports.^16,17,35,36,49,50^ However, the WO_*x*_ monomer supported on rutile has a square pyramidal geometry, which has been reported before for W^6+^.^19,51^

Brønsted acid sites form on all TiO_2_-supported WO_*x*_ clusters at low temperatures (<600 K), regardless of size. Conversely, Brønsted acid sites only become favorable for WO_*x*_ trimers on SiO_2_. Combining structures with different cluster sizes on a given support into one free energy model, we find that at catalytically relevant conditions, such as those for carboxylic acid reduction (Figures S29b and S33b, 423 K, 0.01 kPa H_2_O and 5 kPa H_2_),^6^ the WO_*x*_ supported on both anatase and rutile titania preferentially form monomers with one Brønsted acid site, whereas the silica-supported catalysts favor the formation of trimers with one Brønsted acid site. Formation of Brønsted acid sites at similar conditions was reported for a WO_3_ monolayer on TiO_2_^52^ and Pt-supported WO_*x*_ trimers.^12^ However, the Pt-supported WO_*x*_ trimers show the formation of oxygen vacancies resulting in open coordination sites at *T* ≳ 433 K. In contrast, our results show that open coordination sites on the W (nominally Lewis acid sites) are not favorable under any conditions with *T* < 1100 K. Taken together, Brønsted acid sites appear to form on supported WO_*x*_ materials at catalytically relevant conditions, regardless of the support composition. Thus, Brønsted acid sites would be expected to contribute as active sites, as previously suggested by others.^5,8^

In 5% H_2_ (the XPS and XAS reducing treatment), the oxidation state of W across different cluster sizes and stoichiometries does not change significantly on either titania support. The projected W DOS analyses from HSE06 calculations (Sections S.2.8 and S.2.9) demonstrate little to no variation in the integrated DOS for W among different species, suggesting no significant change in W-oxidation states, consistent with the Bader charge analysis. This result is also consistent with the XPS (and in situ XANES up to 773 K) of the titania-supported samples, which showed little change of W-oxidation state with increasing temperature. The trimer supported on anatase is the only exception, showing some reduction of W with both H addition and O removal (Figures 12 and S37b). This result suggests that larger WO_*x*_ domain sizes on TiO_2_ may regain the ability to reduce by localizing charge on W instead of the TiO_2_ support, consistent with a recent report for charge localization on hydrogenated WO_3_ monolayers supported on anatase.^52^

The relatively nonreducible behavior of small WO_*x*_ clusters on TiO_2_ observed in both our experiments and calculations conflicts with observations for WO_*x*_ on other supports and other oxide clusters supported on TiO_2_. Molybdenum oxide species on TiO_2_ are reported to reduce from Mo^6+^ to Mo^5+^.^53−55^ The W in platinum-supported WO_*x*_ trimers also reduces from +6 to a mixture of +6 and +5 species in H_2_ with an increase in temperature.^12^ Likewise, our experimental and computational results for SiO_2_ showed that the W reduces from ca. + 6 to +5. Taken together, these findings indicate that the mechanism for charge transfer between small WO_*x*_ clusters and TiO_2_ diverges from these other examples, motivating us to next explore a comparison between TiO_2_ and SiO_2_ in more detail.

### 2.4 Charge Density Comparison for SiO_2_ and TiO_2_-Supported WO_*x*_

The results from the XPS and in situ XAS experiments on the SiO_2_ supported Pd–W samples indicate the W reduces from a +6 oxidation state to primarily +5 during TPR. Interestingly, while the TPR profiles of TiO_2_ supported samples (Figure 4) suggested there was much larger hydrogen uptake from both 1Pd–6W–P25–TiO_2_ and 6W–P25–TiO_2_ relative to the TiO_2_ supports alone, the tungsten oxide species did not show a significant change in oxidation state in both XPS and XAS.

**Figure 13:**
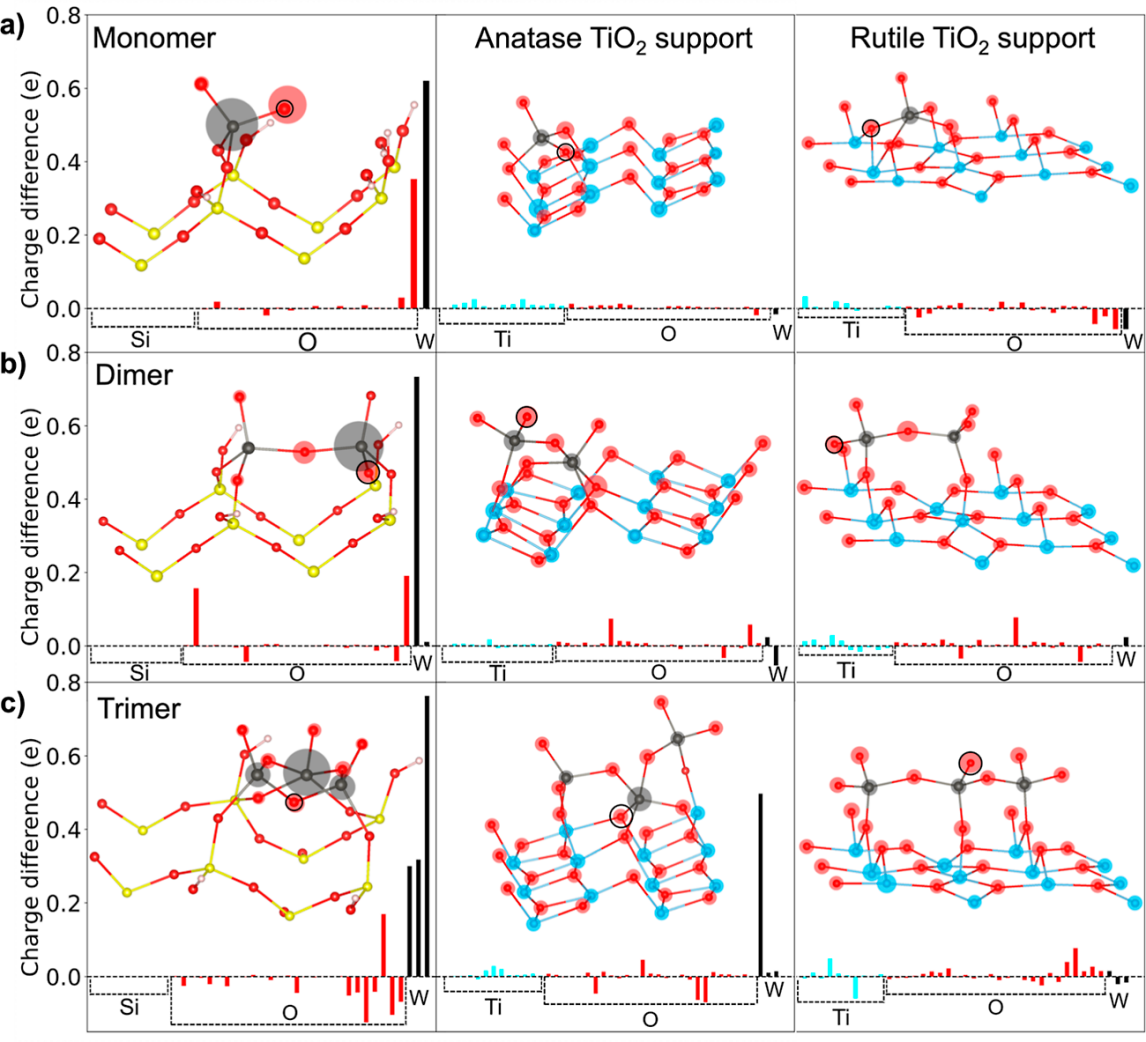
Differences in charge density of surface atoms on silica, anatase, and rutile titania supports for H addition to the (a) monomer (b) dimer and (c) trimer. Each bar represents the charge difference for one atom. The inset molecular figure, truncated after the first support layer, shows filled circles around the atoms that indicate the absolute charge difference after H addition. The radius of the circle is proportional to the change in absolute charge for the individual structures. The highest and lowest absolute charge difference for each structure is assigned a radius for the filled circle and the circles for the remaining atoms are scaled relatively. The molecular figure indicates how the charge is localized and is not a scale representation of the bar graph. The black circle shows where the H atom was added.

To elucidate the difference in the W-oxidation state between the two supports, we computed the charge densities of the surface atoms for the different supports following O-removal or H-addition. Figure 13 shows the charge differences on the WO_*x*_ clusters and surface atoms with the addition of 1H (to the most energetically preferred position) on the SiO_2_ and TiO_2_ supports. For the SiO_2_-supported WO_*x*_ clusters, the redistribution of charge with the addition of 1H atom (effectively 1 e^–^ added to the system) is largely localized to the W atom(s), in agreement with the change in oxidation state for the W experimental and computational results. For the WO_*x*_ monomer on SiO_2_, a significant charge difference also occurs on the O atom that H was added to. In contrast, the additional charge for the TiO_2_-supported cluster is largely distributed among the surface Ti and O atoms (and subsurface since the surface charge does not integrate to 1 e^–^), in agreement with a recent report that shows delocalization of charge across multiple surface TiO_2_ atoms with H addition.^56^ Similarly, for WO_*x*_ dimers and trimers supported on SiO_2_, the charge from the additional H atom is largely localized on the W and the bridging O atoms. The WO_*x*_ trimer supported on anatase (the exception noted in Section 2.3.2) has a larger charge difference on one of the W atoms; however, it is still lower than the overall charge differences for W on SiO_2_. The charge differences for O-removal from the WO_*x*_ species on all supports are reported in Figures S41 and S42 and show analogous results, with much larger charge delocalization and redistribution on TiO_2_ relative to SiO_2_.

## 3 Conclusions

The synthesis and reducibility of supported tungsten oxide clusters are influenced by several factors. Silica-supported tungsten oxide clusters prepared by incipient wetness impregnation (IWI) form primarily 1–3 nm sized clusters but could be made smaller by acid-treating the silica and utilizing a lower loading of W. Titania-supported tungsten oxide clusters are highly dispersed and subnanometer in size on P25–TiO_2_. Results from H_2_-TPR show addition of Pd on W–SiO_2_ aids in the reduction of WO_*x*_ by decreasing its initial reduction temperature, suggesting a significant influence of hydrogen spillover associated with Pd, whereas TiO_2_-supported W showed little difference in initial reduction temperature with added Pd. The hydrogen spillover associated with the Pd aided in the reduction of W in SiO_2_–supported tungsten oxide species from a +6 oxidation state to a mixture of +6 and +5 at 600 K. At higher reduction temperatures (up to 1000 K), W on silica was primarily in the +5 oxidation state regardless of Pd promotion. In contrast, the addition of Pd did not appreciably change the oxidation state of W in TiO_2_-supported WO_*x*_ species. Charge analysis of TiO_2_-supported model WO_*x*_ clusters revealed that charge delocalization across the titania support during H_2_ exposure accounts for the lack of significant W-oxidation state change during nominally reducing treatments. On both titania and silica, WO_*x*_ clusters prefer to remain in a tetrahedral configuration regardless of the reduction state. At temperatures and H_2_ partial pressures relevant to catalysis, computational results from WO_*x*_ clusters on silica and titania reveal that Brønsted acid sites are likely to be present, as undercoordinated W atoms (potential Lewis acid sites) are thermodynamically unfavorable. Taken together, our computational and experimental results demonstrate that the size and reducibility of supported WO_*x*_ clusters are greatly impacted by the electronic properties of the support.

## 4 Methods

### 4.1 Sample Synthesis

High-purity SiO_2_ (Sigma-Aldrich, Davisil 635, 60 Å, 480 m^2^ g^–1^, 150–250 μm) was used for the SiO_2_-supported samples. For acid-treated SiO_2_ (AT-SiO_2_) samples, SiO_2_ was first treated in 13 M HNO_3_ at 373 K for 20 h, washed with distilled, deionized (DDI) water to a pH of 5–6, and dried at 393 K overnight. Silica-supported W samples were synthesized by IWI, in which a desired amount of ammonium metatungstate (Aldrich, 99.99%), was mixed with DDI water to achieve a solution that was equal to the pore volume of the support, which was then added dropwise onto the support until the point of incipient wetness. Samples were dried overnight in air at room temperature, followed by a 2 h drying period in air at 393 K, and thermally treated at 923 K in 100 cm^3^ min^–1^ flowing medical air (Praxair) for 4 h. For the Pd–W–(AT)–SiO_2_ sample, the same incipient wetness procedure was followed with previously synthesized W–SiO_2_ samples, using tetraaminepalladium(II) nitrate solution (10 wt % in H_2_O, Sigma-Aldrich) as the Pd precursor with the same drying and thermal treatment conditions as well. Samples are labeled as (Pd)–*x*W–SiO_2_ where *x* is the nominal weight percent of W.

Titania-supported samples used mixed phase TiO_2_ (Sigma-Aldrich, P25 nanopowder, 21 nm), and Rutile-TiO_2_ (R–TiO_2_) (Sigma-Aldrich, nanopowder, <100 nm, 99.5%) as the supports for (Pd)–*x*W–P25–TiO_2_ and (Pd)–*x*W–R–TiO_2_ samples. Titania supports labeled P25–TiO_2_ without any Pd and/or W designate TiO_2_ materials after a thermal treatment at 923 K in 100 cm^3^ min^–1^ flowing medical air (Praxair) for 4 h. Synthesis of Pd and/or W-incorporated samples utilized fresh P25–TiO_2_ without any prior pretreatment. The same IWI procedures were followed as with the SiO_2_ supported samples. The samples were then dried and thermally treated as previously described for the SiO_2_-supported samples. Reference materials WO_3_ (Alfa Aesar, 99.998%), WO_2_ (Alfa Aesar, 99.9%), and Na_2_WO_4_·2H_2_O (Sigma, ≥99.0%) were used as received from suppliers. For the 1Pd–WO_3_ sample, a similar IWI procedure was followed as for the W–SiO_2_ and W–TiO_2_ supported with the same Pd precursor and bulk WO_3_ as the support. Analogous drying and thermal treatment procedures as mentioned previously were used as well.

### 4.2 Sample Characterization

Dihydrogen TPR was carried out with a Micromeritics AutoChem II 2920 system equipped with a TCD detector. Nonsupported samples, Pd–WO_3_, WO_3_, and WO_2_, were not exposed to any pretreatment and 0.05 g of sample was used. For SiO_2_ and TiO_2_-supported samples, 0.3 g of sample were used and a sample was first heated to 773 K under O_2_ and cooled to 323 K prior to introduction of the reducing gas mixture of 5% H_2_ in Ar at 30 cm^3^ min^–1^. Temperature of the sample was ramped at 10 K min^–1^ to 1223 K and held for 20 min.

DR UV–visible spectra were collected on a PerkinElmer Lambda 850+ UV–vis spectrometer with a Harrick Praying Mantis Diffuse Reflection Accessory. Polytetrafluoroethylene (Sigma-Aldrich, powder, >40 μm) was used as the reflectance standard, with spectra recorded from 190 to 600 nm. Direct optical band gaps were calculated from Tauc plots^18^ with an example provided in Figure S3. Due to the overlap of the TiO_2_ band with tungsten oxide (Figure S2) we report results only for silica-supported WO_*x*_ in the main text.

X-ray photoemission spectroscopy (XPS) measurements were performed using a PHI VersaProbe III spectrometer equipped with monochromatic Al K-alpha X-rays (1486.6 eV) and a hemispherical analyzer. A pass energy of 23 eV and an X-ray beam size of 100 μm were used for high-resolution region scans. An internal electron flood gun (1 eV) and low energy Ar ion gun were utilized during data collection as neutralization systems. Samples were pressed into a Cu grid and exposed to a reducing gas mixture of 5% H_2_/N_2_ flowing at 30 cm^3^ min^–1^ inside a reaction chamber, and the temperature was ramped at 40 K min^–1^ until the desired set point, followed by a hold for 20 min. Following a cool down to ambient temperature, sample transfer from the reaction chamber to the analysis chamber was performed under high vacuum.

High temperature reduction of certain samples did not provide a characteristic C 1s peak. Instead, the Si 2p peak at 103.5 eV or Ti 2p peak at 458.7 eV was used as a charge reference.^57^ The binding energy difference for the W 4f_7/2_ peak between reference WO_3_ and WO_2_ has been reported to be in the range of 2.8–3.0 eV, while the difference between WO_3_ and W metal is in the range of 4.3–4.5 eV.^26,27,58^ The difference between the W 4f_7/2_ and W 4f_5/2_ peaks was kept constant at 2.18 eV, thus only the 7/2 peak values in subsequent figures are provided for brevity. The Ti 3p peaks in the W 4f region were constrained based on the position of the TiO_2_ support Ti 3p peaks following the same reduction procedure. All peak locations and fitting parameters can be found in the Supporting Information.

XAS at the W L_III_ edge was performed using beamline 8-ID at the National Synchrotron Light Source II at Brookhaven National Lab operating at 3.0 GeV and a beam current of 400 mA.^59^ A W metal foil (EXAFS Materials) was used as a reference for the W (11544.0 eV) L_III_ edge. Transmission studies were performed using a high-throughput cell with a temperature controller and Kapton windows as previously described.^60^ Although the SiO_2_-supported samples were able to get a reasonable edge jump in transmission mode, the absorption of the TiO_2_ required that fluorescence data be collected. The fluorescence cell utilized quartz capillaries (1.5 mm diameter, 75 mm length, Friedrich & Dimmock, Inc.) with samples added to the glass tube and fluorescence photons collected on a Passivated Implanted Planar Silicon detector. Initially a flow rate of 10 cm^3^ min^–1^ of He is used for the pre-TPR run while 20 cm^3^ min^–1^ of 5% H_2_/N_2_ flowed for the reduction experiments at high temperature and upon cooling to room temperature after TPR. The XAS spectra were subsequently processed using the Demeter software package.^61^ The oxidation state of the W in each sample was estimated using the L_III_ edge. The edge position at a step height of unity was determined for a sample simultaneously with that of the reference foil collected with each sample to account for any deviations from run to run and ensure that there were no artifacts from the white line. Tungsten foil (EXAFS Materials), powdered WO_2_ (Alfa Aesar, 99.9%), and powdered WO_3_ (Alfa Aesar, 99.998%) were used as oxidation state standards at the W L_III_ edge. The powders were pressed before addition to Kapton tape for placement into the beam path for measurement. The W L_III_ edge XANES for the W foil, WO_2_, and WO_3_ are plotted in Figure S15. The edge energy evaluated at the unity value of the absorption coefficient of the standards was used to make a calibration curve, Figure S18, that was utilized to estimate the changes in the oxidation state of the W in the sample during reduction.

XRD patterns were obtained on a PANalytical Empyrean diffractometer using Cu–Kα radiation (λ of 1.54 Å) generated at 45 kV with a 40 mA incident electron source. Scans were collected in the range of 2θ = 15–80° with a 0.015° step size. Rietveld refinement was performed with the Maud program.

X-ray fluorescence (XRF) measurements were performed by Horiba Scientific (Piscataway, NJ) with an XGT-9000 XRF analytical microscope equipped with a 50 W Rh anode X-ray tube. Spectra were collected in a partial vacuum over an area of 12.5 mm^2^, an energy resolution of less than 143 eV at Mn–Kα, and an accelerated voltage of 50 keV. Component concentrations were calculated using the Fundamental Parameters Method. Results are tabulated in Table S14.

The HAADF-STEM images were taken on a Thermo Fisher Scientific Themis Z transmission electron microscope operating at 200 kV and equipped with a monochromator and probe correction as well as a SuperX EDX detector. The STEM-HAADF detector (Fischione) collection angle was set to 50–200 mrad at 115 mm camera length. Samples were slurried either in methanol or hexane and deposited on lacey or holey carbon films supported on copper grids.

### 4.3 DFT Calculations

DFT calculations were conducted using the Vienna Ab-Initio Simulation Package (VASP),^54^ version 5.4.4. We used the strongly constrained and appropriately normed (SCAN) functional to describe the exchange–correlation potential and a plane wave cutoff energy of 400 eV. Structures optimized using the SCAN functional were used to generate phase diagrams in Section S.2.11 for different size tungsten oxide clusters and their oxidation states under varying temperature and H_2_ pressure conditions. However, GGA functionals like SCAN do not necessarily have reliable accuracy for the charge distribution on oxides, and are prone to overestimates of charge delocalization.^62−65^ To address this, we used a hybrid functional, Heyd–Scuseria–Ernzerhof functional (HSE06). For structures that had the lowest free energies at the experimental conditions (600 – 1000 K in 5% H_2_ – indicated by dashed line in Section S.2.11) we performed single point energy calculations using HSE06. Phase diagrams in Sections 2.3.1 and 2.3.2 were generated using HSE06 but remain similar to those generated by SCAN, with the main difference being the computed DOS, as described in Section S.2.12. Initial structures for the bulk phases were taken from the Materials Project database.^66^ For bulk structure optimizations we used the Monkhorst–Pack *k*-point mesh reported in Materials Project.^66^ Cell vectors for bulk structures were optimized. Vibrational contributions to free energy were neglected since we expect them to be similar across the materials studied here. Additionally, our phase diagrams generally show large free energy separations between the lowest energy phases and others, which would not be affected by vibrational free energy contributions due to small differences in the total number of bonds between the structures compared.

Stoichiometric slabs were constructed from optimized bulk cells using the Python Materials Genomic (*Pymatgen*)^67^ package, *Slabgenerator* function. Each surface contains at least a 10 Å slab thickness, and to prevent interactions between surfaces in the *z* direction, a vacuum space of 12 Å was added. For the SiO_2_ calculations the bottom two layers of the structures were frozen, and for TiO_2_ (anatase and rutile) the bottom layer was frozen. The convergence criteria for all calculations (bulk and slab) were electronic energies converged to 10^–6^ eV and atomic forces to less than 0.03 eV/Å. The *k*-point mesh for each slab (in the *x* and *y* directions) was estimated using the *k*-points per reciprocal Å for the bulk structure and rounding up, and a single *k*-point was used in the *z* direction (where vacuum was added). We used DFT calculations with the same parameters described above with the HSE06 functional to compute single point energies, except for the *k*-point mesh, where we rounded down instead of up (due to the large computational expense of these calculations), relative to the *k*-points per reciprocal Å for the bulk structure.

The amorphous nature of the silica support presents challenges for modeling, requiring an ensemble of molecular models^68,69^ rather than a single structure. To avoid this complication, but preserve the electronic properties of SiO_2_, we used the β-crystabolite-SiO_2_ (001) surface as a surrogate model, which has been previously used as a reasonable computational model for amorphous silica.^37^ We obtained the bulk β-crystabolite-SiO_2_ structure from the Materials Project database and subsequently optimized its cell vectors and atomic positions. Pymatgen^67^ was used to generate the (001) symmetric slab from the optimized bulk structure, and the slab was then hydroxylated by adding H atoms to each terminal O atom (8 on each side). The atomic positions of the slab were then optimized, and the bottom two layers of the slab were fixed for the subsequent calculations.

To determine the most thermodynamically stable WO_*x*_ species on each support (MO_2_, M = Ti or Si) at different conditions, we evaluated the relative free energy of all the structures considered (Sections 2.3.1 and 2.3.2). Details of our thermodynamic analyses are in Section S.2.13.

### 4.4 Basin-Hopping Global Optimization for TiO_2_

Since there are several ways to graft a WO_*x*_ cluster onto a support, we used a global optimization scheme, basin-hopping,^70^ to automate the grafting process for the TiO_2_ surfaces and checked for low energy configurations. For the TiO_2_-supported clusters, at every iteration, W atom(s) and each atom bonded to W is translated by a random distance, up to 5 Å in the *x* and *y* direction and 0.5 Å in the *z* direction. The surface atoms that are not bonded to W atom(s) are not randomly perturbed but are allowed to relax during the local geometry optimization occurring at each iteration in basin-hopping. We used this scheme with 40 iterations to allow the cluster to navigate across different configurations on the surface and increase the probability of finding the lowest energy configuration. We found going past 40 iterations, or using different initial guess structures, did not result in any new lower energy structures. Basin-hopping was used to find the lowest energy configuration for the WO_*x*_ monomer, dimer and trimer supported on both TiO_2_ surfaces. The silanol groups on the silica surface introduce additional complexity, constraining the cluster movement. Hence, we did not use basin-hopping optimization for the silica-supported WO_*x*_ clusters. We also analyzed the effect of periodic image interactions for different cell sizes (varying the density of WO_*x*_) and found negligible changes. More details are provided in Section S.2.14.

### 4.5 Bader Charge Analysis and DOS

Bader charge analysis was performed using the method developed by Henkelman et al.^71,72^ We assign formal oxidation states of +6 and +4 to the charge densities of WO_3_ and WO_2_, respectively. These states were used as a calibration to assign oxidation states to W in the supported clusters. The charge analysis provides a quantitative understanding of the electron distribution and oxidation state changes occurring during the interaction of tungsten with the supports. We used VASPKIT^73^ to analyze and visualize the DOS and projected W-DOS of different supported WO_*x*_ clusters. Additional details for the DOS calculations are provided in Section S.2.12.

## Supporting Information Available

Supporting Information provides additional experimental and computational data including high resolution HAADF-STEM images, DR UV–vis spectra (including reference W materials) and select SiO_2_ and P25–TiO_2_ supported samples, TPR profiles, XPS spectra and fitting parameters, XAS spectra and fitting parameters (including reference W materials), and XRD diffraction patterns of select SiO_2_ and P25–TiO_2_ supported samples; details for silica supported models, thermodynamic phase diagrams for combined domain sizes, projected W DOS, charge differences for O removal reaction, thermodynamic analysis using SCAN, details on thermodynamic relations and optimized structure files are also provided (PDF)
(ZIP)
