# Supplementary material for: Influence of Domain Size and Support Composition on the Reducibility of SiO2 and TiO2 Supported Tungsten Oxide Clusters
Source: J Phys Chem C Nanomater Interfaces. 2024 Aug 14;128(33):13864–78. doi: 10.1021/acs.jpcc.4c03652 (PMC11345822; doi:10.1021/acs.jpcc.4c03652)
Supplement: Supplementary file 1 — jp4c03652_si_001.pdf [file jp4c03652_si_001.pdf]

Supplemental Information for:

Influence of Domain Size and Support Composition on the Reducibility of SiO_2 and TiO_2 Supported Tungsten Oxide Clusters

Konstantin Mamedov[a], Anukriti Shrestha[a], Colby. A. Whitcomb, Christopher Paolucci*, and Robert. J. Davis*

Department of Chemical Engineering, University of Virginia, Charlottesville, VA 22903, United States

[a] These authors contributed equally to the manuscript, i.e., they are co-first authors

*Corresponding Authors, Emails: cp9wx@virginia.edu and rjd4f@virginia.edu

Table of Contents

S.1: Supplemental Information for Experiments S7

- S.1.1: XPS Peak Fitting Parameters S13
- S.1.2: XAS Edge Positions and Spectra S21
- S.1.3: Rietveld Refinement and XRD Patterns S25
- S.1.4: Composition Analysis of Samples S26

S.2: Supplemental Information for Computational Work S27

- S.2.1: Amorphous Silica Support S27
- S.2.2: Reduction of bulk WO_3 .... S28
- S.2.3: Generation of Tungsten Oxide Clusters on Silica S29
- S.2.4: Phase Diagrams for Combined Domain Sizes on SiO_2 .... S33
- S.2.5: WO_x Supported on Silica with HSE06 .... S34
- S.2.6: Phase Diagrams for Combined Domain Sizes on TiO_2 .... S37
- S.2.7: Vacancy Generation on Rutile TiO_2 .... S38
- S.2.8: WO_x Supported on Anatase Titania with HSE06 .... S39
- S.2.9: WO_x Supported on Rutile Titania with HSE06 .... S42
- S.2.10: Charge Differences for O Removal Reaction S45
- S.2.11: Phase Diagrams and Oxidation States Using SCAN S47
- S.2.12: Density of States S48
- S.2.13: Thermodynamic Relations S49
- S.2.14: Cell Size Effects on Rutile TiO_2 .... S50

S.3: References S50

Supplemental Figures

Figure S1: Additional high resolution HAADF-STEM images of 3W-SiO_2, 6W-SiO_2, and 1Pd-6W-SiO_2 with elemental mapping for Pd, W, and Si over the same region S7

Figure S2: DR UV-Vis spectrum of P25-TiO_2 support and 3W-P25-TiO_2 showing the high absorption of the P25-TiO_2 support relative to WO_x species S8

Figure S3: Example Tauc plots showing calculated direct bandgap of 2W-AT-SiO_2 sample S8

Figure S4: Normalized DR UV-Vis spectra of reference W materials and 3W-SiO_2 sample..... S9

Figure S5: Additional high resolution HAADF-STEM images of 3W-P25-TiO_2, 6W-P25-TiO_2, and 1Pd-6W-P25-TiO_2 samples.. S10

Figure S6: Temperature-programed reduction profiles of 1Pd-P25-TiO_2, P25-TiO_2, and R-TiO_2 samples.. S11

Figure S7: Temperature-programed reduction profiles of 1Pd-3W-SiO_2 and 3W-SiO_2 samples... S12

Figure S8: Temperature-programed reduction profiles of 1Pd-2W-AT-SiO_2 and 2W-AT-SiO_2 samples.. S12

Figure S9: Temperature-programed reduction profiles of reference bulk WO_2 and WO_3 as well as 1Pd-WO_3 samples.. S13

Figure S10: Photoemission spectra and peak fits of the Pd 3d region for 1Pd-6W-SiO_2 sample **a)** prior to (fresh) and following a treatment in 5%H_2/N_2 **b**) 400 K and **c)** 600 K.......................... S15

Figure S11: Photoemission spectra of normalized Ti 2p region for P25-TiO_2 support prior to and following a treatment in 5%H_2/N_2 at 400, 600, 800, and 1000 K... S16

Figure S12: Photoemission spectra of normalized Ti 3p region for P25-TiO_2 support prior to and following a treatment in 5%H_2/N_2 at 400, 600, 800, and 1000 K... S17

Figure S13: Photoemission spectra and peak fits of the W4f (and Ti 3p) region for 6W-P25-TiO_2 of the as-synthesized sample.. S17

Figure S14: Photoemission spectra and peak fits of the Pd 3d region for 1Pd-6W-P25-TiO_2 sample **a)** prior to (fresh) and following a treatment in 5%H_2/N_2at **b**) 400 K and **c)** 600 K S20

Figure S15: Ambient ex situ XANES spectra of the standard W samples at the W L_{III} edge... S21

Figure S16: In situ XANES spectra of the W L_{III} edge before and after a TPR at 773 K under a flow of 5% H_2/N_2 of 6W-SiO_2.. S22

Figure S17: In situ XANES spectra of the W L_{III} edge before and after a TPR at 773 K under a flow of 5% H_2/N_2 of 6W-P25-TiO_2 .... S22

Figure S18: Position of the L_{III} edge of W for each standard sample (W Foil, WO_2, and WO_3) at an edge jump of 1 μ(E) S24

Figure S19: X-ray diffraction patterns of 6W-P25-TiO_2, P25-TiO_2-(NP), and R-TiO_2 .... S25

Figure S20: Ab initio thermodynamic phase diagram for WO_x monomer supported on β-crystabolite-SiO_2 (001) compared to amorphous silica supported monomer. S27

Figure S21: Reaction energies for forming different bulk WO_x structures. Green shaded structures were generated from H-addition, and blue shaded structures from O-removal. S28

Figure S22: Ab initio thermodynamic phase diagram for bulk WO_3. .... S28

Figure S23: Different configurations of silica-supported WO_x monomer with +5 and +6 W formal oxidation states S29

Figure S24: Free energy diagram for the different configurations considered in **Figure S23** under synthesis conditions S30

Figure S25: Different configurations of silica-supported WO_x dimer with +5 and +6 W formal oxidation states S31

Figure S26: Free energy diagram for the different configurations considered in **Figure S25** under synthesis conditions S31

Figure S27: Different configurations of silica-supported WO_x trimer with +5 and +6 W formal oxidation states S32

Figure S28: Free energy diagram for the different configurations considered in **Figure S27** under synthesis conditions S32

Figure S29: Free energy diagram for all sizes of tungsten oxide cluster on silica support under a) synthesis conditions and b) reaction conditions S33

Figure S30: a) Projected W DOS for silica-supported WO_x monomers. b) Integrated projected W DOS. Computed using the HSE06 functional S34

Figure S31: a) Projected W DOS for silica-supported WO_x dimers. b) Integrated projected W DOS. Computed using the HSE06 functional S35

Figure S32: a) Projected W DOS for silica-supported WO_x trimers. b) Integrated projected W DOS. Computed using the HSE06 functional S36

Figure S33: Free energy for all sizes of tungsten oxide cluster on both anatase and rutile titania support under a) synthesis conditions and b) reaction conditions S37

Figure S34: Ab initio thermodynamic phase diagram for rutile TiO_2 supported WO_x monomer, and dimer with surface O vacancies. S38

Figure S35: a) Projected W DOS for anatase titania-supported WO_x monomers. b) Integrated projected W DOS. Computed using the HSE06 functional... S39

Figure S36: a) Projected W DOS for anatase titania-supported WO_x dimers. b) Integrated projected W DOS. Computed using the HSE06 functional... S40

Figure S37: a) Projected W DOS for anatase titania-supported WO_x trimers. b) Integrated projected W DOS. Computed using the HSE06 functional... S41

Figure S38: a) Projected W DOS for rutile titania-supported WO_x monomers. b) Integrated projected W DOS. Computed using the HSE06 functional. S42

Figure S39: a) Projected W DOS for rutile titania-supported WO_x dimers. b) Integrated projected W DOS. Computed using the HSE06 functional... S43

Figure S40: a) Projected W DOS for rutile titania-supported WO_x trimers. b) Integrated projected W DOS. Computed using the HSE06 functional... S44

Figure S41: Differences in charge density of surface atoms on silica support. Generated using the HSE06 functional.. S45

Figure S42: Differences in charge density of surface atoms on anatase and rutile titania support. Generated using the HSE06 functional... S46

Figure S43: Ab initio thermodynamic phase diagram for silica supported WO_x monomer, dimer, and trimer at P_{H2O} = 0.01 kPa. S47

Figure S44: **a)** Ab initio thermodynamic phase diagram for rutile TiO_2 supported WO_x monomer, dimer, and trimer. **b)** Ab initio thermodynamic phase diagram for anatase TiO_2 supported WO_x monomer, dimer, and trimer S47

Figure S45: Integrated projected W DOS for silica-supported WO_x monomers, generated using the SCAN functional.. S48

Supplemental Tables

Table S1: Direct Bandgaps and LMCT positions of reference W materials and SiO_2 supported W catalysts.. S8

Table S2: Hydrogen consumption per mol W for select W catalysts S11

Table S3: XPS peak fitting parameters for 6W-SiO_2 and 1Pd-6W-SiO_2 samples following a 600 K reducing treatment in 5% H_2/N_2.. S13

Table S4: XPS peak fitting parameters for 6W-SiO_2 and 1Pd-6W-SiO_2 samples following a 800 K reducing treatment in 5% H_2/N_2.. S14

Table S5: XPS peak fitting parameters for 6W-SiO_2 and 1Pd-6W-SiO_2 samples following a 1000 K reducing treatment in 5% H_2/N_2.. S14

Table S6: XPS peak fitting parameters for the Pd 3d region on the 1Pd-6W-SiO_2 sample prior to (fresh) and following a 400 K and 600 K reducing treatment in 5% H_2/N_2 .... S16

Table S7: XPS peak fitting parameters for 6W-P25-TiO_2 sample as-synthesized and without a reducing treatment S18

Table S8: XPS peak fitting parameters for 6W-P25-TiO_2 and 1Pd-6W-P25-TiO_2 samples following a 600 K reducing treatment in 5% H_2/N_2 .... S18

Table S9: XPS peak fitting parameters for 6W-P25-TiO_2 and 1Pd-6W-P25-TiO_2 samples following an 800 K reducing treatment in 5% H_2/N_2 .... S19

Table S10: XPS peak fitting parameters for 6W-P25-TiO_2 and 1Pd-6W-P25-TiO_2 samples following a 1000 K reducing treatment in 5% H_2/N_2 .... S19

Table S11: XPS peak fitting parameters for the Pd 3d region on the 1Pd-6W-P25-TiO_2 sample prior to (fresh) and following a 400 K and 600 K reducing treatment in 5% H_2/N_2 .... S20

Table S12: Position of the W L_{III} edge at an edge jump of 1 $\mu(E)$ for various WO_x samples and standards S23

Table S13: Phase composition by weight % of TiO_2 samples calculated from Rietveld Refinement via X-ray diffraction patterns.. S25

Table S14: X-ray Fluorescence composition of select samples by weight % of Pd and W S26

Table S15: Grafting energy for WO_x trimer on different rutile TiO_2 surface supercell size...... S50

S.1: Supplemental Information for Experiments

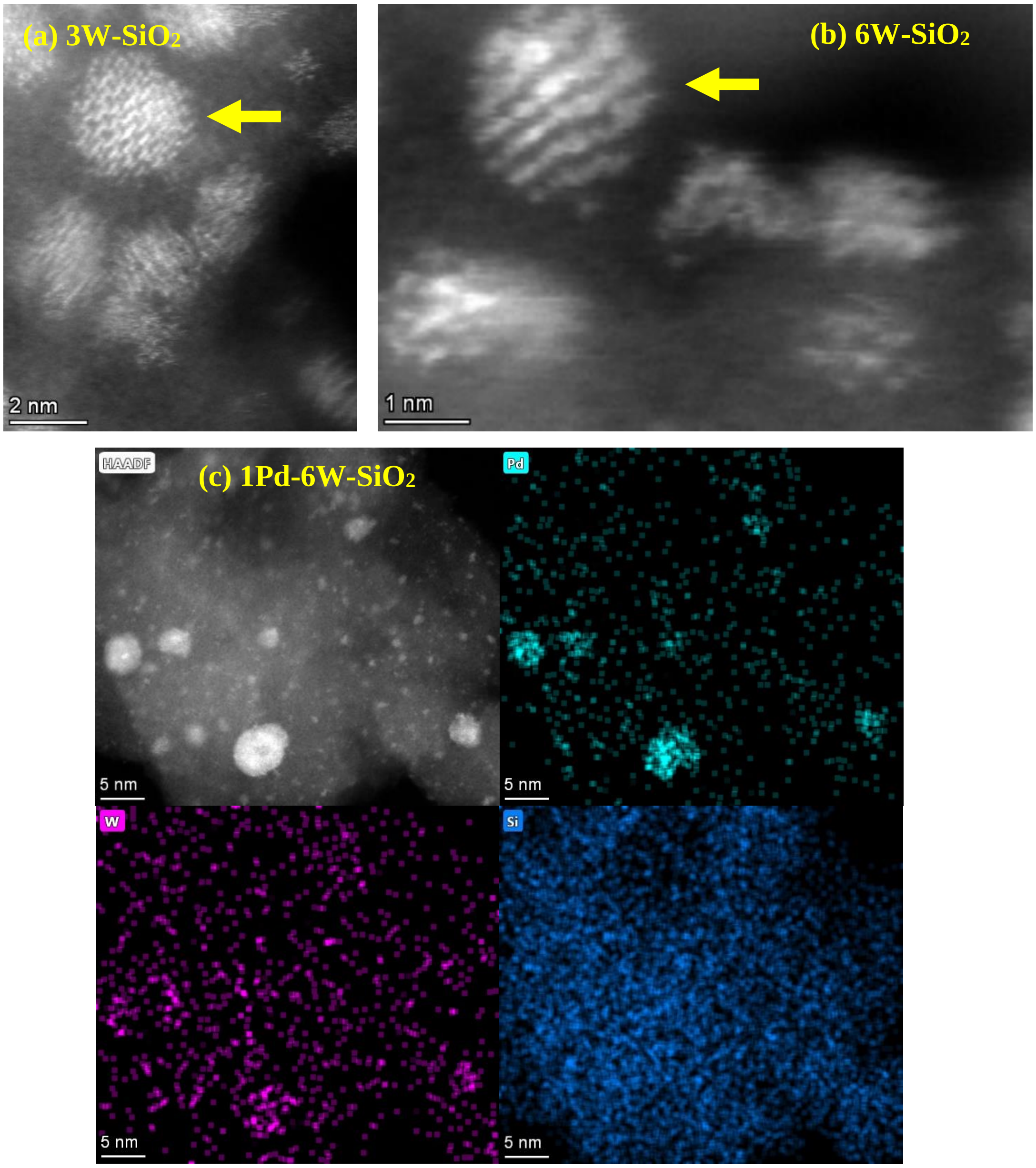

Figure S1: Additional high resolution HAADF-STEM images of a) 3W-SiO_2 and b) 6W-SiO_2 at a resolution of 2 and 1 nm, respectively and c) 1Pd-6W-SiO_2 with elemental mapping for Pd, W, and Si over the same region.

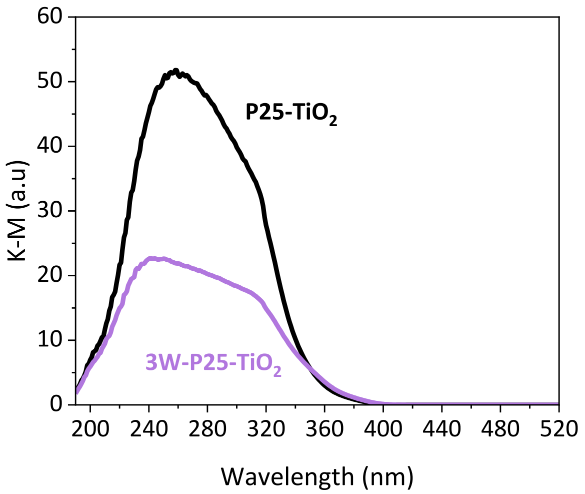

Figure S2: DR UV-Vis spectrum of P25-TiO_2 support and 3W-P25-TiO_2 showing the high absorption of the P25-TiO_2 support relative to WO_x species.

Table S1: Direct Bandgaps and LMCT positions of reference W materials and SiO_2 supported W catalysts

Sample	Band Gap (eV)	LMCT Band (nm)
Na_2WO_4	5.1	225
2W-AT-SiO_2	4.8	221
3W-SiO_2	4.1	261
6W-SiO_2	4.0	270
$(NH_4)_6H_2W_{12}O_{40}$	3.4	318
WO_3	2.8	378

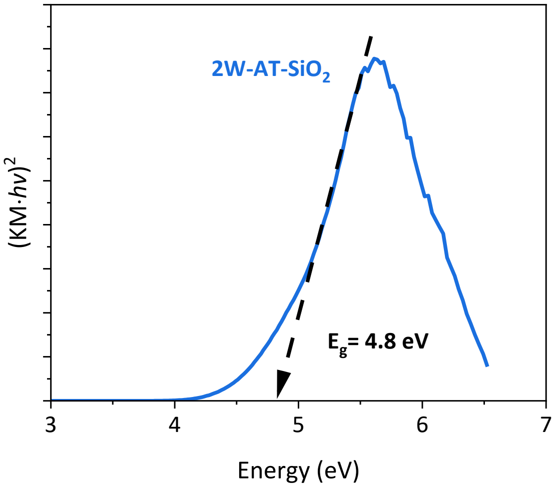

Figure S3: Example Tauc plot showing calculated direct bandgap of 2W-AT-SiO_2 sample.

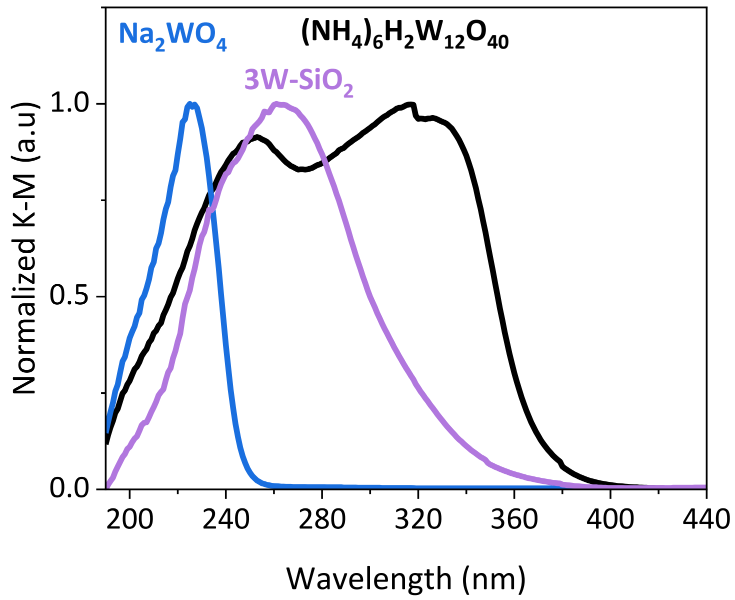

Figure S4: Normalized DR UV-Vis spectra of reference W materials and 3W-SiO_2 sample.

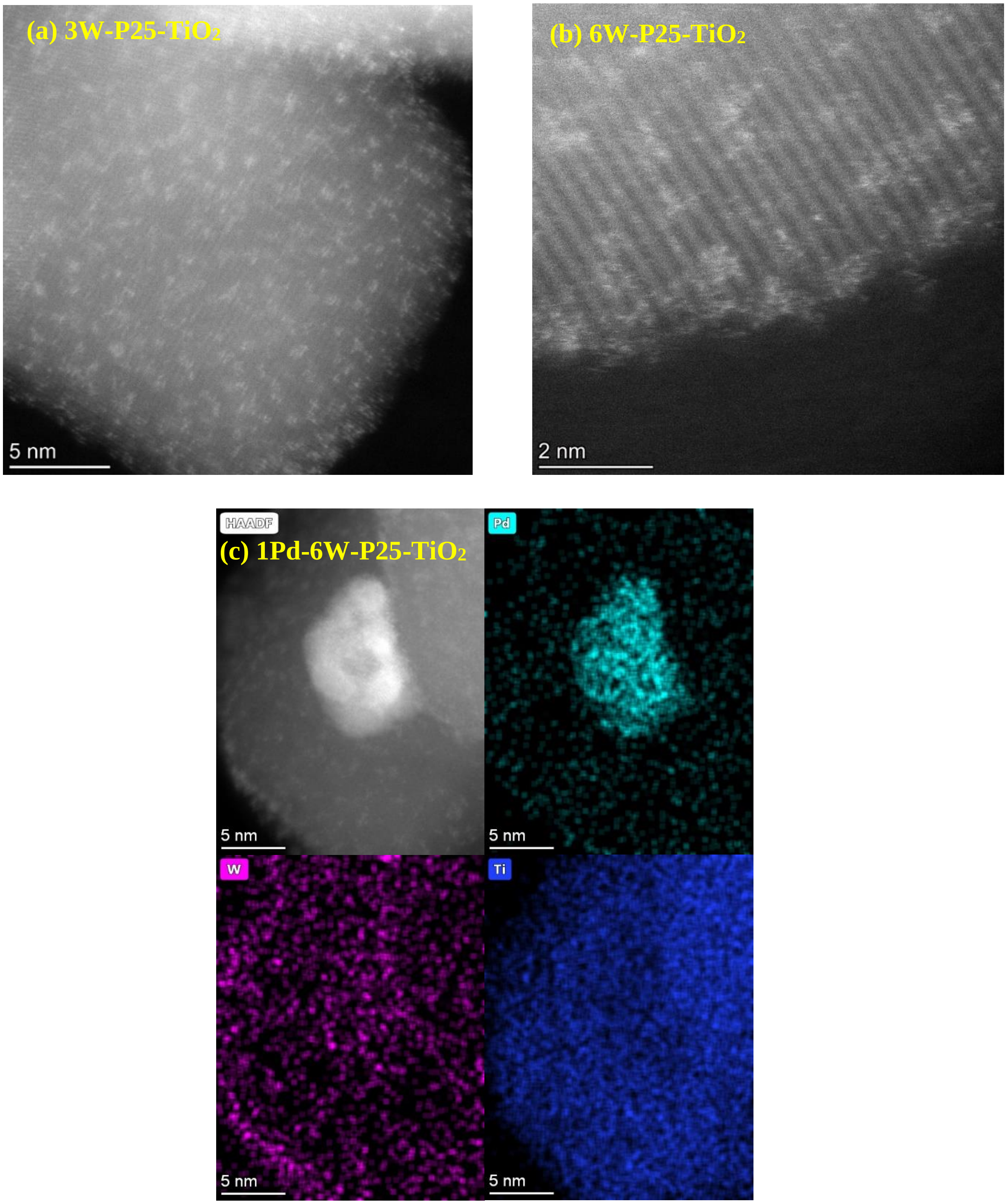

Figure S5: Additional high resolution HAADF-STEM images of a) 3W-P25-TiO_2 and b) 6W-P25-TiO_2 at a resolution of 5 and 2 nm, respectively, and c) 1Pd-6W-P25-TiO_2 with elemental mapping for Pd, W, and Ti over the same region.

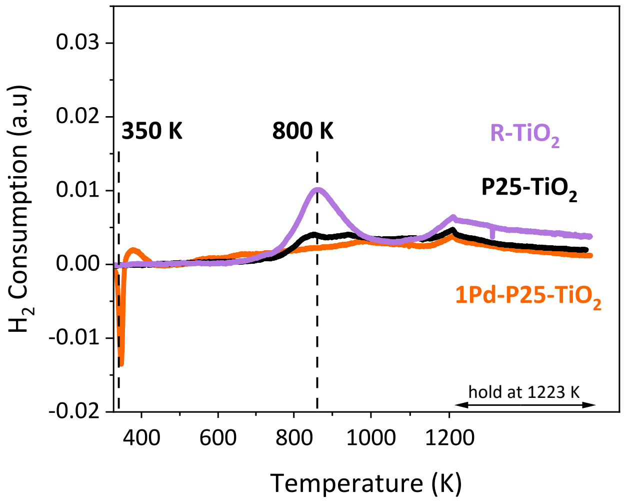

Figure S6: Temperature-programed reduction profiles with a ramp rate of 10 K·min^{-1} to 1223 K and hold for 20 min under a flow of 5% H_2/Ar at 30 cm^3 min^{-1} of 1Pd-P25-TiO_2, P25-TiO_2, and R-TiO_2 samples.

Table S2: Hydrogen consumption per mol W for select W catalysts

Sample	Mol H_2/mol W
WO_2	1.9
WO_3	2.8
1Pd-WO_3	2.7
1Pd-2W-AT-SiO_2	0.9
2W-AT-SiO_2	0.5
3W-SiO_2	0.9
1Pd-3W-SiO_2	1.0
6W-SiO_2	0.8
1Pd-6W-SiO_2	1.1
6W-P25-TiO_2	1.0*
1Pd-6W-P25-TiO_2	1.5*

Mol W calculated from XRF measurements listed in **Table S14.** *Background reduction of P25-TiO_2 support.

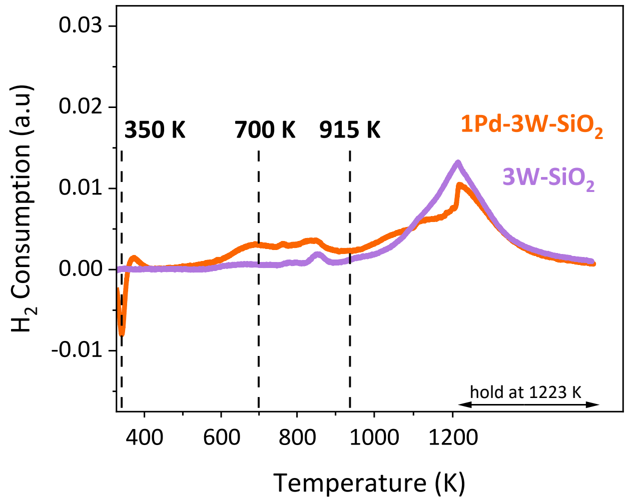

Figure S7: Temperature-programed reduction profiles with a ramp rate of 10 K min^{-1} to 1223 K and hold for 20 min under a flow of 5% H_2/Ar at 30 cm^3 min^{-1} of 1Pd-3W-SiO_2 and 3W-SiO_2 samples.

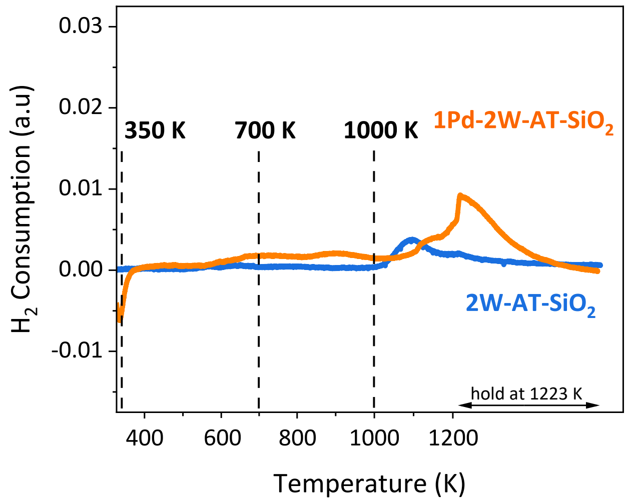

Figure S8: Temperature-programed reduction profiles with a ramp rate of 10 K min^{-1} to 1223 K and hold for 20 min under a flow of 5% H_2/Ar at 30 cm^3 min^{-1} of 1Pd-2W-AT-SiO_2 and 2W-AT-SiO_2 samples.

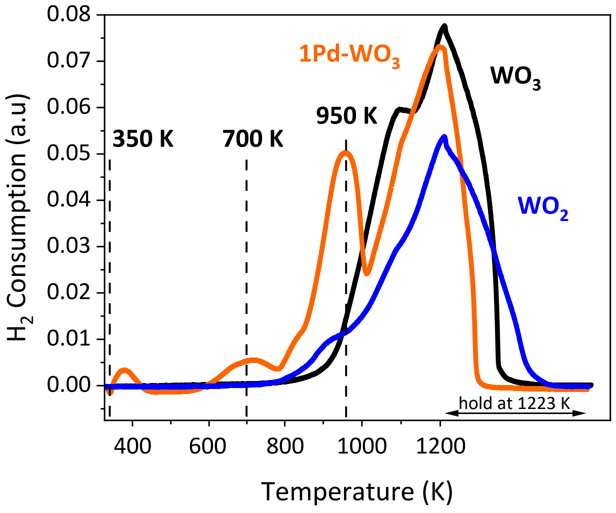

Figure S9: Temperature-programed reduction profiles with a ramp rate of 10 K min^{-1} to 1223 K and hold for 20 min under a flow of 5% H_2/Ar at 30 cm^3 min^{-1} of reference bulk WO_2 and WO_3 as well as 1Pd-WO_3 samples.

S.1.1: XPS Peak Fitting Parameters

Table S3: XPS peak fitting parameters for 6W-SiO_2 and 1Pd-6W-SiO_2 samples following 600 K reducing treatment in 5% H_2/N_2.

Figure #	Sample	Band	Position	FWHM	%Area	Chi Squared
6a	6W-SiO_2	W^{6+} $4f_{7/2}$	36.7	2.5	57.14	1.1
		$W^{6+}4f_{5/2}$	38.9	2.5	42.86	
	1Pd-6W-SiO_2	$W^{5+}4f_{7/2}$	35.4	2.2	25.62	1.8
		$W^{5+}4f_{5/2}$	37.6	2.2	31.52	
		$W^{6+}4f_{7/2}$	36.6	2.2	19.22	
		$W^{6+}4f_{5/2}$	38.7	2.2	23.64	

Spacing between 7/2 and 5/2 peaks of each species was held constant at 2.18 eV.
FWHM value was set as equal across all peaks in each sample.

Table S4: XPS peak fitting parameters for 6W-SiO_2 and 1Pd-6W-SiO_2 samples following 800 K reducing treatment in 5% H_2/N_2.

Figure #	Sample	Band	Position	FWHM	%Area	Chi Squared
6b	6W-SiO_2	$W^{5+}4f_{7/2}$	34.9	1.6	36.34	1.6
		W^{5+} $4f_{5/2}$	37.0	1.6	20.80	
		W^{6+} $4f_{7/2}$	36.5	1.6	27.26	
		W^{6+} $4f_{5/2}$	38.7	1.6	15.60	
	1Pd-6W-SiO_2	$W^{5+}4f_{7/2}$	35.2	1.6	43.96	3.6
		$W^{5+}4f_{5/2}$	37.4	1.6	13.18	
		W^{6+} $4f_{7/2}$	36.6	1.6	32.97	
		W^{6+} $4f_{5/2}$	38.8	1.6	9.88	

Spacing between 7/2 and 5/2 peaks of each species was held constant at 2.18 eV.
FWHM value was set as equal across all peaks in each sample.

Table S5: XPS peak fitting parameters for 6W-SiO_2 and 1Pd-6W-SiO_2 samples following 1000 K reducing treatment in 5% H_2/N_2.

Figure #	Sample	Band	Position	FWHM	%Area	Chi Squared
6c	6W-SiO_2	$W^{5+}4f_{7/2}$	35.3	1.5	44.19	1.4
		$W^{5+}4f_{5/2}$	37.4	1.5	12.95	
		W^{6+} $4f_{7/2}$	36.7	1.5	33.14	
		W^{6+} $4f_{5/2}$	38.9	1.5	9.71	
	1Pd-6W-SiO_2	$W^{5+}4f_{7/2}$	35.1	1.4	45.77	3.0
		$W^{5+}4f_{5/2}$	37.3	1.4	11.37	
		W^{6+} $4f_{7/2}$	36.3	1.4	34.33	
		W^{6+} $4f_{5/2}$	38.5	1.4	8.53	

Spacing between 7/2 and 5/2 peaks of each species was held constant at 2.18 eV.
FWHM value was set as equal across all peaks in each sample.

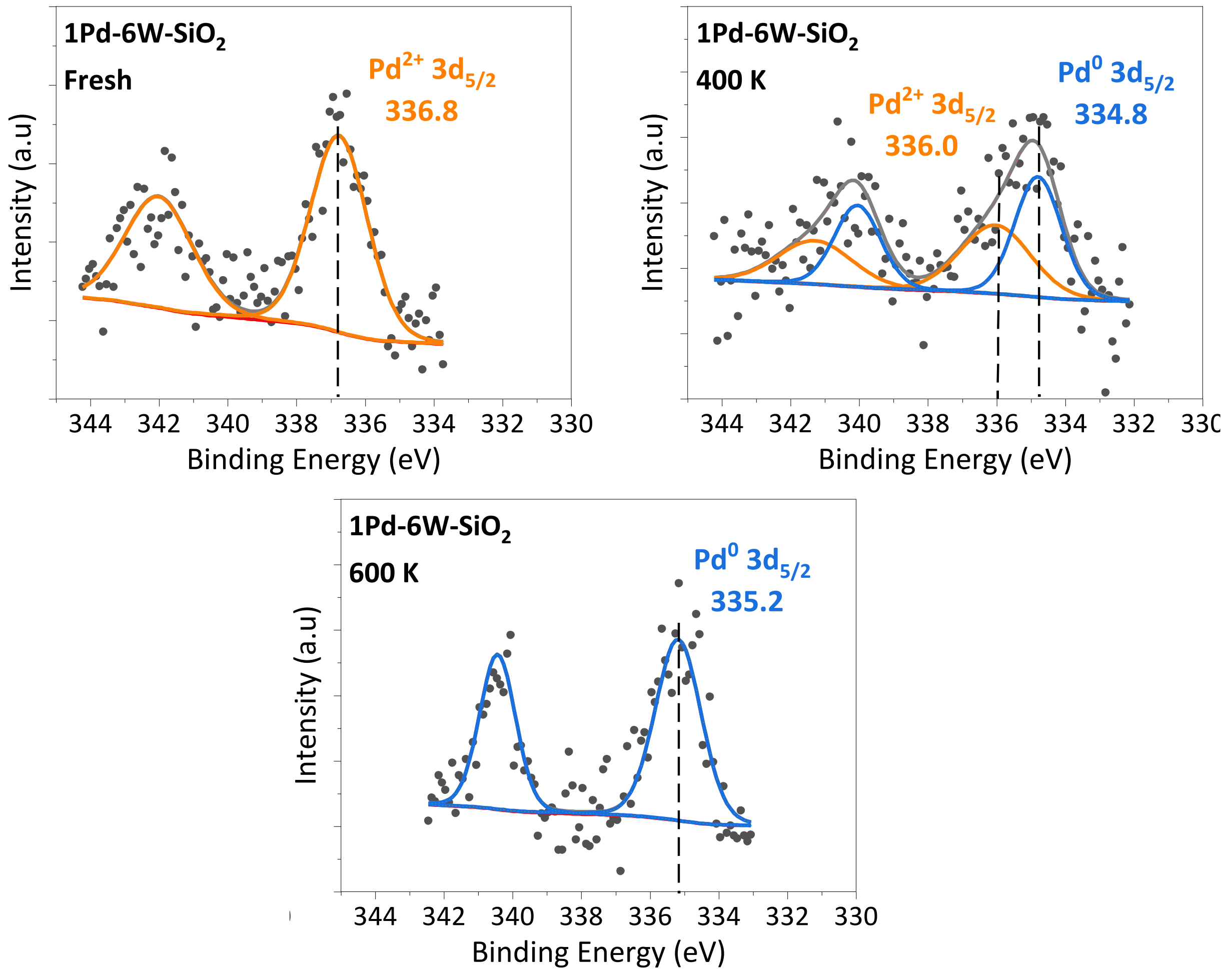

Figure S10: Photoemission spectra and peak fits of the Pd 3d region for 1Pd-6W-SiO_2 sample **a)** prior to (fresh) and following a treatment in 5% H_2/N_2 at 30cm^3 min^{-1} at **b**) 400 K and **c)** 600 K. Spectra were charge referenced to the Si 2p peak at 103.5 eV.

Table S6: XPS peak fitting parameters for the Pd 3d region on the 1Pd-6W-SiO_2 sample prior to (fresh) and following a 400 K and 600 K reducing treatment in 5% H_2/N_2.

Figure #	Sample	Band	Position	FWHM	%Area	Chi Squared
S10	Fresh	Pd^{2+} $3d_{5/2}$	336.8	2.0	59.88	0.85
		Pd^{2+} $3d_{3/2}$	342.0	2.3	40.12	
	400 K	Pd^{0} $3d_{5/2}$	334.8	1.6	32.30	1.19
		Pd^{0} $3d_{3/2}$	340.1	1.6	21.64	
		Pd^{2+} $3d_{5/2}$	336.0	2.4	27.58	
		Pd^{2+} $3d_{3/2}$	341.3	2.5	18.48	
	600 K	Pd^{0} $3d_{5/2}$	335.2	1.6	59.88	1.08
		Pd^{0} $3d_{3/2}$	340.4	1.2	40.12	

Spacing between 5/2 and 3/2 peaks of each species was held constant at 5.26 eV.

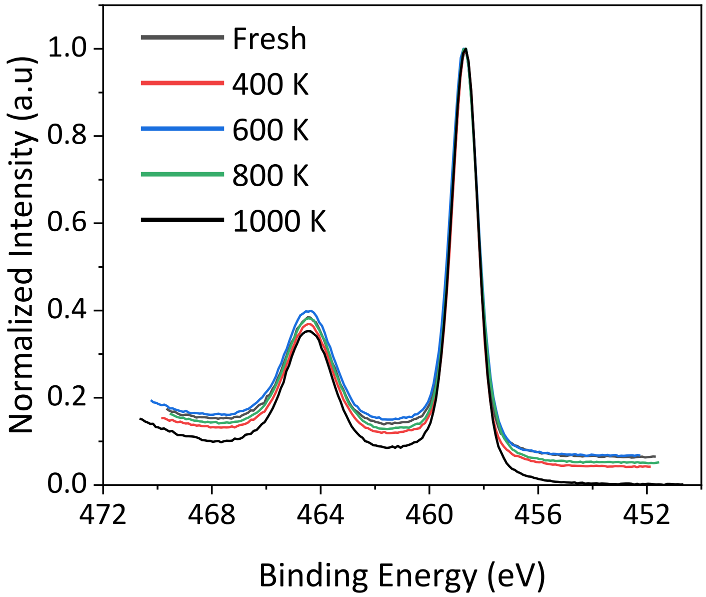

Figure S11: Photoemission spectra of normalized Ti 2p region for P25-TiO_2 support prior to and following a treatment in 5% H_2/N_2 at 30 cm^3 min^{-1} at 400, 600, 800, and 1000 K. Spectra were charge referenced to the Ti 2p peak at 458.7 eV.

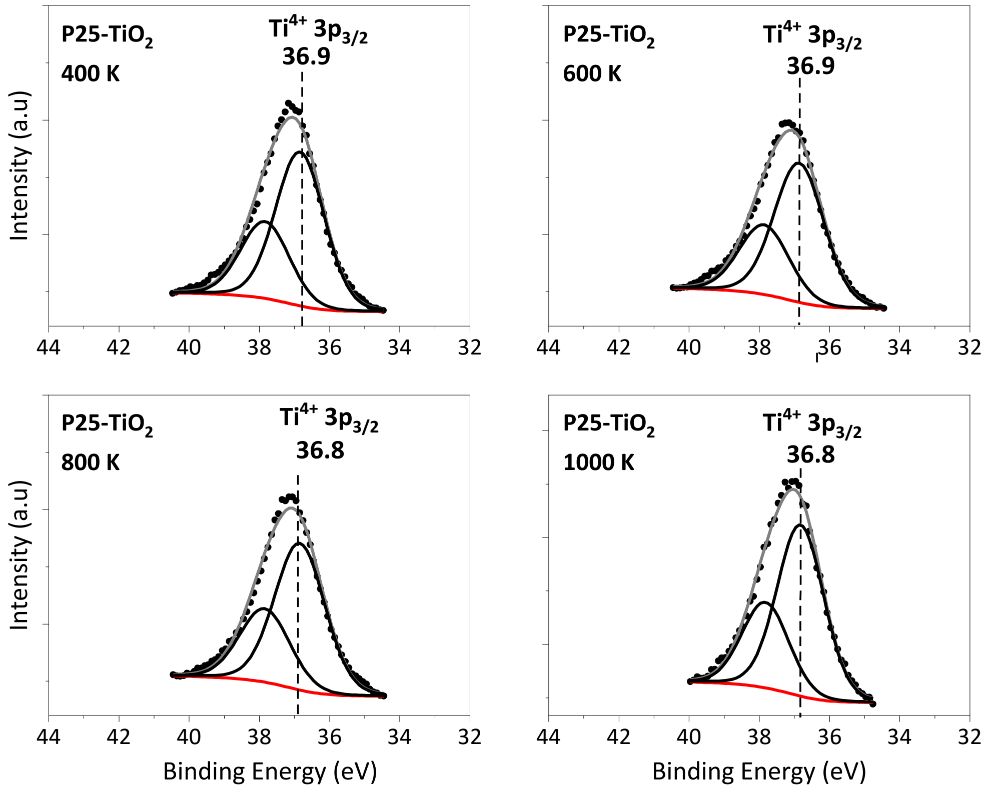

Figure S12: Photoemission spectra of normalized Ti 3p region for P25-TiO_2 support prior to and following a treatment in 5% H_2/N_2 at 30 $cm^3\ min^{-1}$ at 400, 600, 800, and 1000 K. Spectra were charge referenced to the Ti 2p peak at 458.7 eV.

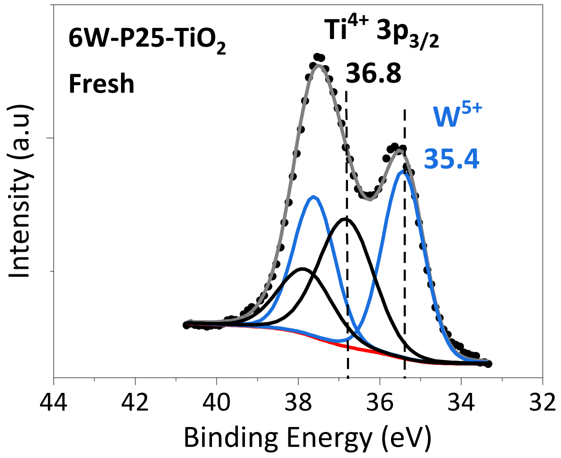

Figure S13: Photoemission spectra and peak fits of the W 4f (and Ti 3p) region for 6W-P25-TiO_2 of the as-synthesized sample. Spectra were charge referenced to the Ti $2p_{3/2}$ peak at 458.7 eV.

Table S7: XPS peak fitting parameters for 6W-P25-TiO_2 sample as-synthesized and without a reducing treatment.

Figure #	Sample	Band	Position	FWHM	%Area	Chi Squared
S13	6W-P25-TiO_2	$W^{5+}4f_{7/2}$	35.4	1.2	32.16	5.3
		W^{5+} $4f_{5/2}$	37.6	1.2	24.12	
		Ti^{4+} $3p_{3/2}$	36.9	1.6	29.15	
		Ti^{4+} $3p_{1/2}$	37.8	1.6	14.57	

Spacing between W4f 7/2 and 5/2 peaks of each species was held constant at 2.18 eV. FWHM value was set as equal across all peaks in each sample. Ti 3p peaks were held constant based on fitting of bare P25-TiO_2 support in **Figure S12**.

Table S8: XPS peak fitting parameters for 6W-P25-TiO_2 and 1Pd-6W-P25-TiO_2 samples following 600 K reducing treatment in 5% H_2/N_2.

Figure #	Sample	Band	Position	FWHM	%Area	Chi Squared
7a	6W-P25-TiO_2	$W^{5+}4f_{7/2}$	35.4	1.2	30.92	12.3
		W^{5+} $4f_{5/2}$	37.6	1.2	23.19	
		Ti^{4+} $3p_{3/2}$	36.9	1.8	26.23	
		Ti^{4+} $3p_{1/2}$	37.9	1.8	19.67	
	1Pd-6W-P25-TiO_2	$W^{5+}4f_{7/2}$	35.3	1.4	33.85	26.8
		W^{5+} $4f_{5/2}$	37.5	1.4	25.39	
		Ti^{4+} $3p_{3/2}$	36.9	1.6	27.18	
		Ti^{4+} $3p_{1/2}$	37.9	1.6	13.59	

Spacing between W4f 7/2 and 5/2 peaks of each species was held constant at 2.18 eV. FWHM value was set as equal across all peaks in each sample. Ti 3p peaks were held constant based on fitting of bare P25-TiO_2 support in **Figure S12**.

Table S9: XPS peak fitting parameters for 6W-P25-TiO_2 and 1Pd-6W-P25-TiO_2 samples following 800 K reducing treatment in 5% H_2/N_2.

Figure #	Sample	Band	Position	FWHM	%Area	Chi Squared
7b	6W-P25-TiO_2	$W^{5+}4f_{7/2}$	35.4	1.2	31.56	6.7
		W^{5+} $4f_{5/2}$	37.6	1.2	23.67	
		Ti^{4+} $3p_{3/2}$	36.8	1.8	29.85	
		Ti^{4+} $3p_{1/2}$	37.8	1.8	14.93	
	1Pd-6W-P25-TiO_2	$W^{5+}4f_{7/2}$	35.4	1.2	31.29	17.4
		W^{5+} $4f_{5/2}$	37.6	1.2	23.46	
		Ti^{4+} $3p_{3/2}$	36.8	1.6	30.17	
		Ti^{4+} $3p_{1/2}$	37.8	1.6	15.1	

Spacing between W4f 7/2 and 5/2 peaks of each species was held constant at 2.18 eV. FWHM value was set as equal across all peaks in each sample. Ti 3p peaks were held constant based on fitting of bare P25-TiO_2 support in **Figure S12**.

Table S10: XPS peak fitting parameters for 6W-P25-TiO_2 and 1Pd-6W-P25-TiO_2 samples following 1000 K reducing treatment in 5% H_2/N_2.

Figure #	Sample	Band	Position	FWHM	%Area	Chi Squared
7c	6W-P25-TiO_2	$W^{5+}4f_{7/2}$	35.4	1.3	33.55	5.3
		W^{5+} $4f_{5/2}$	37.6	1.3	25.17	
		Ti^{4+} $3p_{3/2}$	36.8	1.5	27.52	
		Ti^{4+} $3p_{1/2}$	37.8	1.6	13.76	
	1Pd-6W-P25-TiO_2	$W^{5+}4f_{7/2}$	35.5	1.1	30.09	4.8
		W^{5+} $4f_{5/2}$	37.7	1.1	22.57	
		Ti^{4+} $3p_{3/2}$	36.8	1.8	31.56	
		Ti^{4+} $3p_{1/2}$	37.8	1.7	15.78	

Spacing between W4f 7/2 and 5/2 peaks of each species was held constant at 2.18 eV. FWHM value was set as equal across all peaks in each sample. Ti 3p peaks were held constant based on fitting of bare P25-TiO_2 support in **Figure S12**.

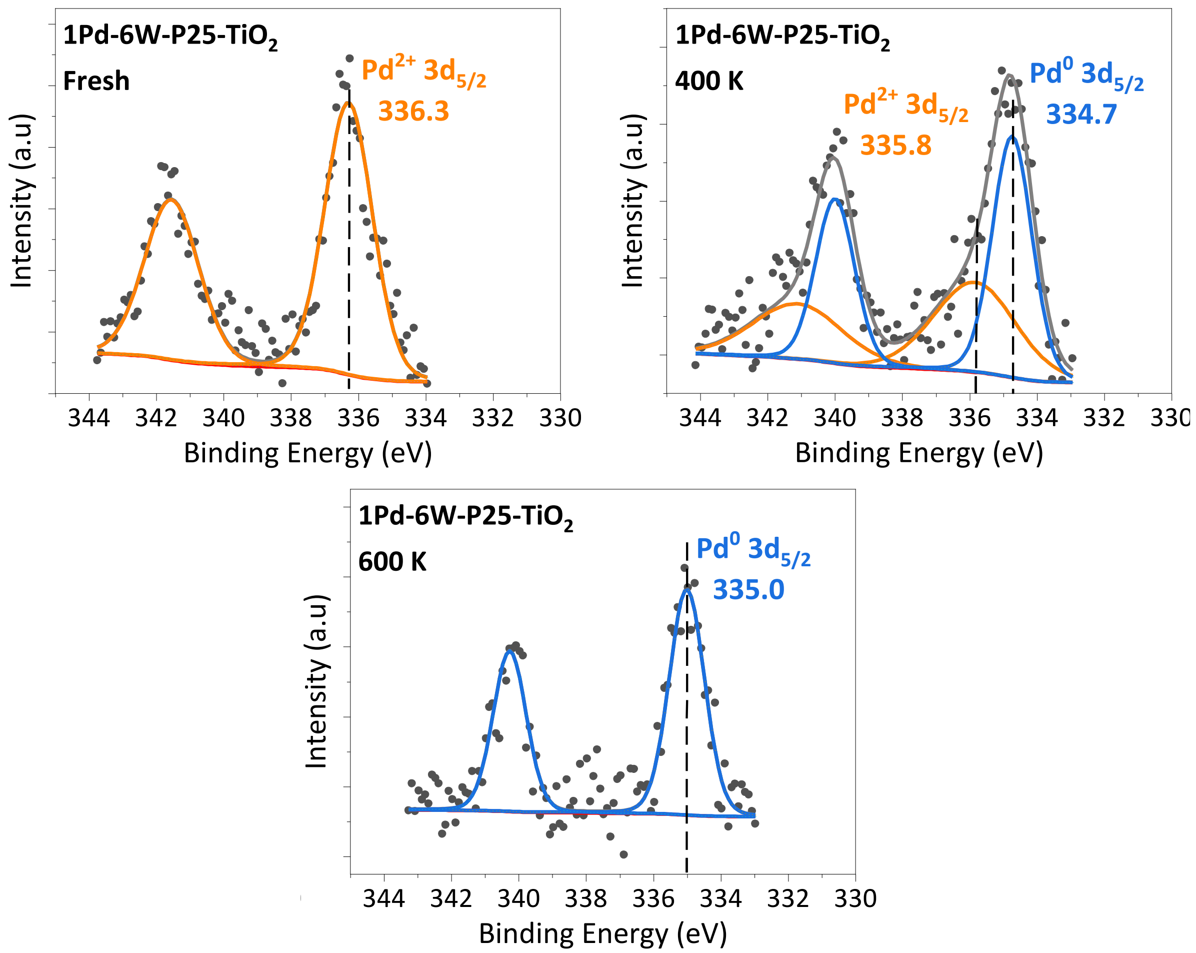

Figure S14: Photoemission spectra and peak fits of the Pd 3d region for 1Pd-6W-P25-TiO_2 sample **a)** prior to (fresh) and following a treatment in 5% H_2/N_2 at 30 cm^3min^{-1} at **b**) 400 K and **c)** 600 K. Spectra were charge referenced to the Ti 2p peak at 458.7 eV.

Table S11: XPS peak fitting parameters for the Pd 3d region on the 1Pd-6W-P25-TiO_2 sample prior to (fresh) and following a 400 K and 600 K reducing treatment in 5% H_2/N_2.

Figure #	Sample	Band	Position	FWHM	%Area	Chi Squared
S14	Fresh	Pd^{2+} $3d_{5/2}$	336.3	1.7	59.88	1.58
		Pd^{2+} $3d_{3/2}$	341.6	1.9	40.12	
	400 K	Pd^{0} $3d_{5/2}$	334.7	1.4	33.81	1.14
		Pd^{0} $3d_{3/2}$	340.0	1.4	22.65	
		Pd^{2+} $3d_{5/2}$	335.8	2.9	26.07	
		Pd^{2+} $3d_{3/2}$	341.1	3.1	17.47	
	600 K	Pd^{0} $3d_{5/2}$	335.0	1.2	59.88	1.11
		Pd^{0} $3d_{3/2}$	340.3	1.2	40.12	

Spacing between 5/2 and 3/2 peaks of each species was held constant at 5.26 eV.

S.1.2: XAS Edge Positions and Spectra

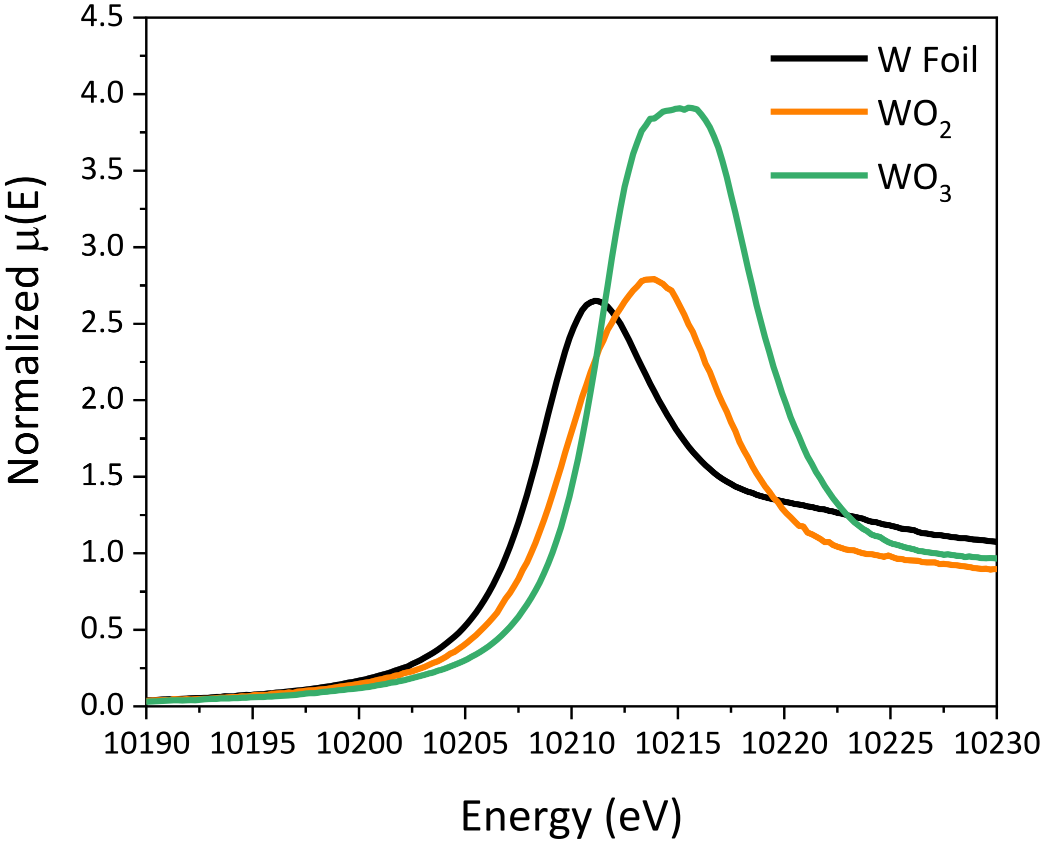

Figure S15: Ambient ex situ XANES spectra of the standard W samples at the W L_{III} edge.

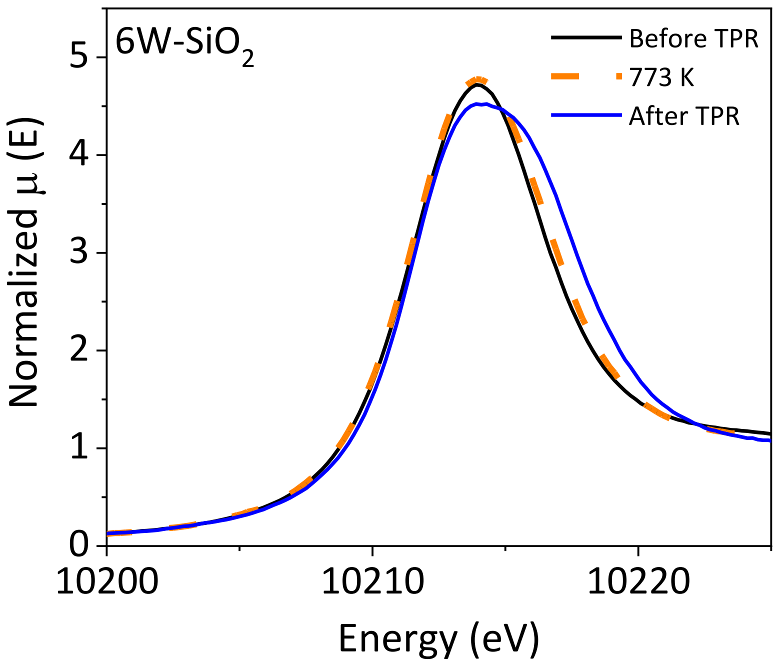

Figure S16: In situ XANES spectra of the W L_{III} edge before and after a TPR at 773 K under a flow of 5% H_2/N_2 at 20 $cm^3\ min^{-1}$ of 6W-SiO_2.

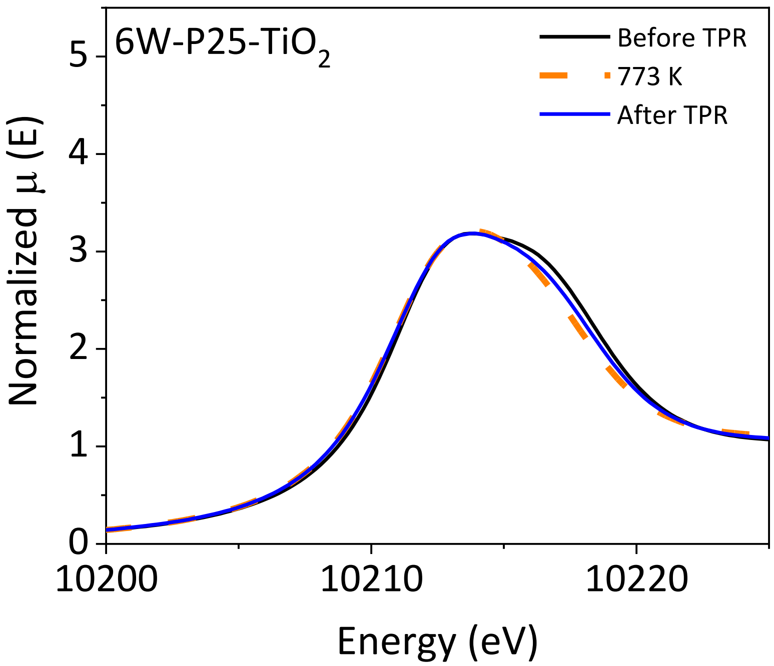

Figure S17: In situ XANES spectra of the W L_{III} edge before and after a TPR at 773 K under a flow of 5% H_2/N_2 at 20 $cm^3\ min^{-1}$ of 6W-P25-TiO_2.

Table S12: Position of the W L_{III} edge at $\mu(E) = 1$ for various samples and standards. The corresponding oxidation states are reported as well after using the calibration curve in **Figure S18**.

Sample	Normalized E (eV)	Oxidation State
W Metal	10207.0	0
WO_2	10208.1	4
WO_3	10209.0	6
1Pd-6W-SiO_2		
Before TPR	10208.8	5.7
At 773 K	10208.2	4.3
After TPR	10208.2	4.3
6W-SiO_2		
Before TPR	10209.1	6.2
At 773 K	10208.9	5.8
After TPR	10208.8	5.7
1Pd-6W-TiO_2		
Before TPR	10208.7	5.5
At 773 K	10208.5	5.0
After TPR	10208.6	5.3
6W-TiO_2		
Before TPR	10208.8	5.7
At 773 K	10208.6	5.3
After TPR	10208.6	5.3

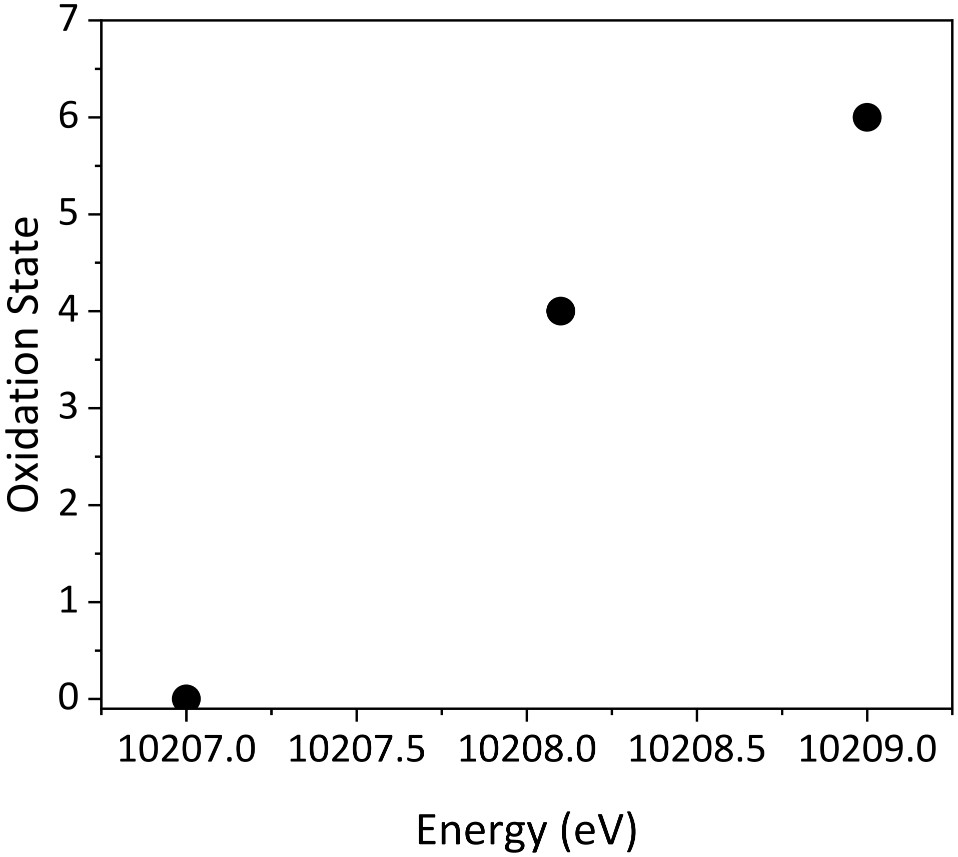

Figure S18: Position of the L_{III} edge of W for each standard sample (W Foil, WO_2, and WO_3) at $\mu(E) = 1$. The corresponding oxidation state for each sample is plotted on the y-axis. The calibration curve was used to interpolate the oxidation state of the prepared samples presented in **Table S12**.

S.1.3: Rietveld Refinement and XRD Patterns

Table S13: Phase composition by weight % of TiO_2 samples calculated from Rietveld Refinement via X-ray diffraction patterns from **Figure S19**

Sample	Anatase	Rutile
TiO_2-P25-NP	91	9
TiO_2-P25	76	24
6W-TiO_2	89	11

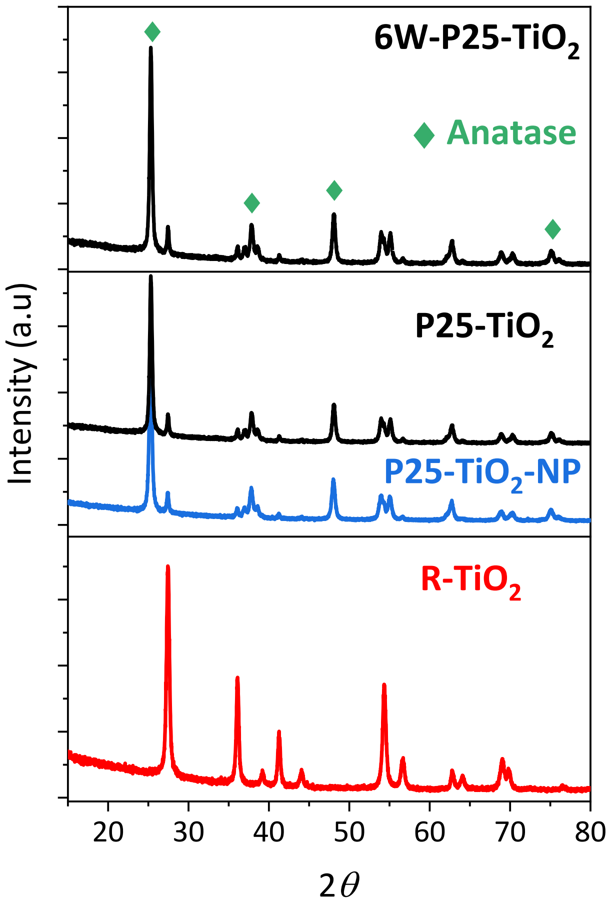

Figure S19: X-ray diffraction patterns of 6W-P25-TiO_2, P25-TiO_2-(NP), and R-TiO_2. Intensity offset for clarity. P25-TiO_2 was calcined at 923 K in 100 cm^3 min^{-1} flowing medical air (Praxair) for 4 h. P25-TiO_2-NP coincides with TiO_2 support that had no pretreatment prior to characterization. R-TiO_2 also had no pretreatment prior to characterization.

S.1.4: Composition Analysis of Samples

Table S14: X-ray Fluorescence composition of select samples by weight % of Pd and W

Sample	Pd	W
1Pd-WO_3	0.8	99.2
1Pd-2W-AT-SiO_2	0.3	2.6
2W-AT-SiO_2	-	2.0
3W-SiO_2	-	3.2
1Pd-3W-SiO_2	0.5	3.1
6W-SiO_2	-	6.6
1Pd-6W-SiO_2	0.7	6.6
1Pd-TiO_2	0.4	-
3W-TiO_2	-	3.3
1Pd-3W-TiO_2	0.5	3.0
6W-TiO_2	-	5.2
1Pd-6W-TiO_2	0.6	5.8

S.2: Supplemental Information for Computational Work

S.2.1: Amorphous Silica Support

Several theoretical studies have used different structures to model amorphous silica supports. Differences in protocols used to generate the structures can result in large energy differences. We used a previously reported slab model that was generated using simulated annealing AIMD simulations.[1] This silica model reproduced ring size distribution, and silanol density and distribution comparable to experiments. The structure generated in the original paper has been validated with experiments using features such as O-Si-O and Si-O-Si angles, Si-O bond lengths, and spectroscopic properties such as NMR parameters and OH vibrational frequencies.

Grafting sites are generated by removing two H atoms from surface silanols which form an H_2O molecule with an O atom from the WO_x cluster and desorb. The WO_x monomer is then grafted and the W center in the monomer form bonds with the dehydrogenated O atoms and is surrounded by four oxygen atoms in a tetrahedral symmetry. The four silanol groups on the surface gives rise to six possible grafting configurations. The reaction energy for grafting the WO_x monomer on the surface is calculated as:

$$\mathrm{SiO}_x\mathrm{H}_8 + \mathrm{WO}_3 \rightarrow \mathrm{SiO}_{x-1}\mathrm{H}_6\mathrm{WO}_3 + \mathrm{H}_2\mathrm{O} \quad (1)$$

$$\Delta E_{\mathrm{grafting}} = E_{\mathrm{SiO}_{x-1}\mathrm{H}_6\mathrm{WO}_3} + E_{\mathrm{H}_2\mathrm{O}} - E_{\mathrm{SiO}_x\mathrm{H}_8} - E_{\mathrm{WO}_3} \quad (2)$$

The grafting energy for all 6 configurations are within a range of 0.56 eV, after which the configuration with the lowest grafting energy is considered for H addition and O removal reactions. **Figure S20** reports the phase diagram for the WO_x monomers on the amorphous support. However, we were unable to graft larger WO_x clusters on the surface because of the placement of the silanol groups. In **Figure S20**, the phase diagram for the WO_x monomer on the amorphous support is qualitatively similar to the monomer supported on β-crystabolite-SiO_2 (001), indicating that this slab can serve as a computational model for the support.

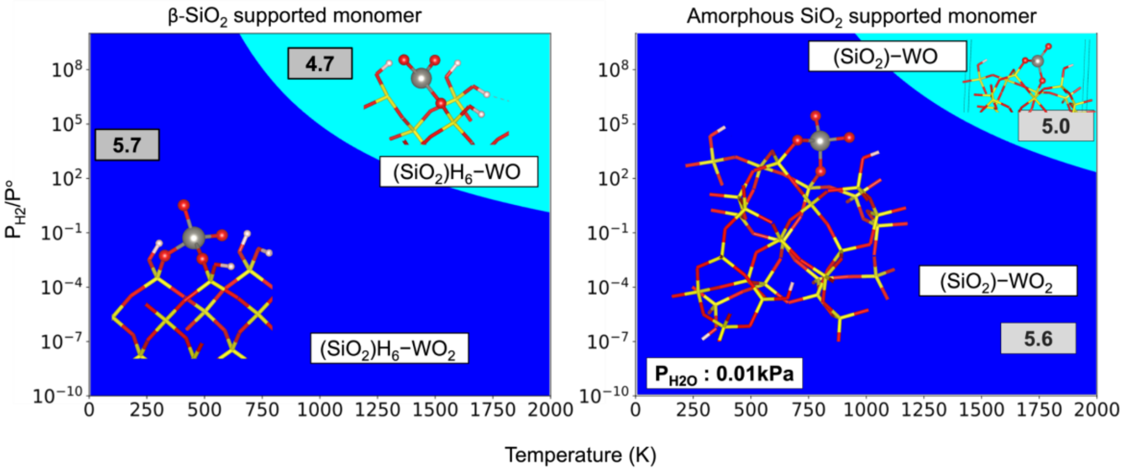

Figure S20: Ab initio thermodynamic phase diagram for WO_x monomer supported on β-crystabolite-SiO_2 (001) compared to amorphous silica-supported WO_x monomer.

S.2.2: Reduction of bulk WO_3

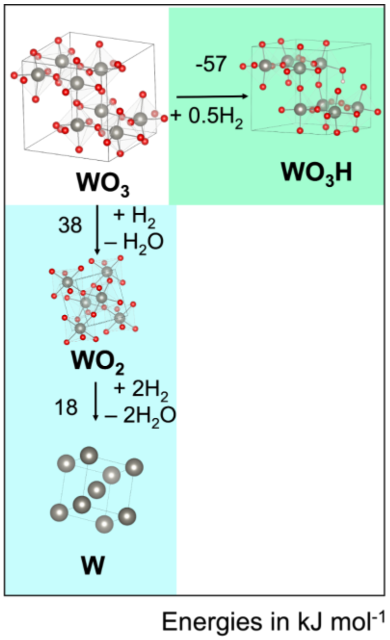

Figure S21: Reaction energies for forming different bulk WO_x structures. Green shaded structures were generated from H-addition, and blue shaded structures from O-removal.

For hydrogen addition in bulk WO_3, we evaluate three symmetrically distinct O atoms,[2] calculating the binding energy for three possible H binding angles on each. From these nine configurations, we select the one with the lowest binding energy for further evaluation.

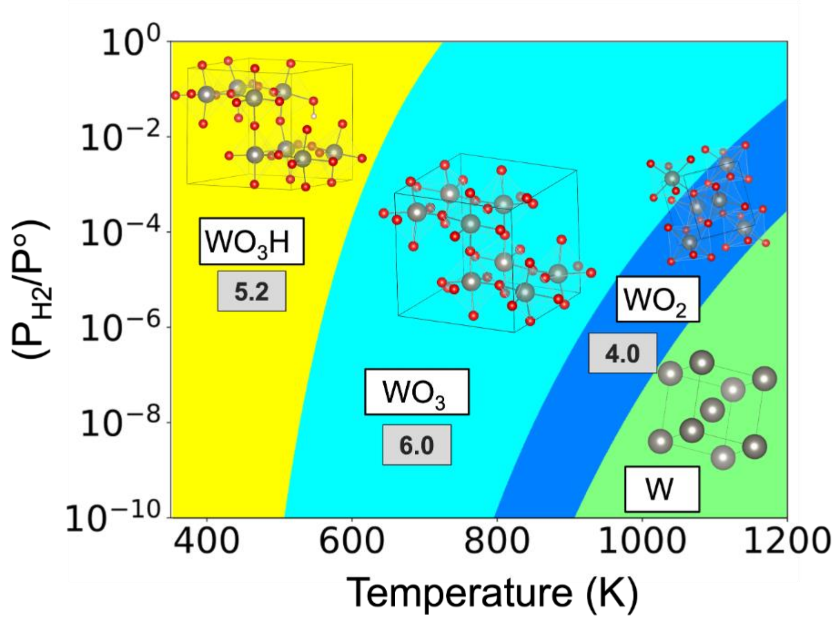

Figure S22: Ab initio thermodynamic phase diagram for bulk WO_3.

S.2.3: Generation of Tungsten Oxide Clusters on Silica

To represent different-sized tungsten oxide clusters, we used W monomers, dimers, and trimers. Grafting these clusters onto the SiO_2 surface requires the removal of at least one surface H atom, enabling W to bind with the surface O atoms. Various grafting configurations were explored by removing between 1 and 4 H atoms from the top of the SiO_2 slab and attaching W to the surface O atom(s). We generated initial W clusters where all W atoms exhibit formal oxidation states of +5 or +6, a choice made based on reported oxidation states for supported W-oxides following high temperature oxidative pretreatments. After grafting a W atom to surface O atom(s), additional O atom(s) and (or) OH group(s) were attached to W to achieve a formal oxidation state of +5 or +6. A maximum limit of 2 -OH groups is set because additional -OH groups would most likely lead to dehydration because of strong thermodynamic drive to form H_2O. Below, we show all generated configurations for monomers, dimers, and trimers, along with the formal oxidation state of all W in the cluster and the calculation of the relative free energy. Red boxes in **Figures S23, S25,** and **S27** indicate the starting species (those with a white background in **Figure 9** in the main text). The zero of free energy represents bulk WO_3 that is not grafted on the β-crystabolite-SiO_2 (001).

<u>Monomers:</u>

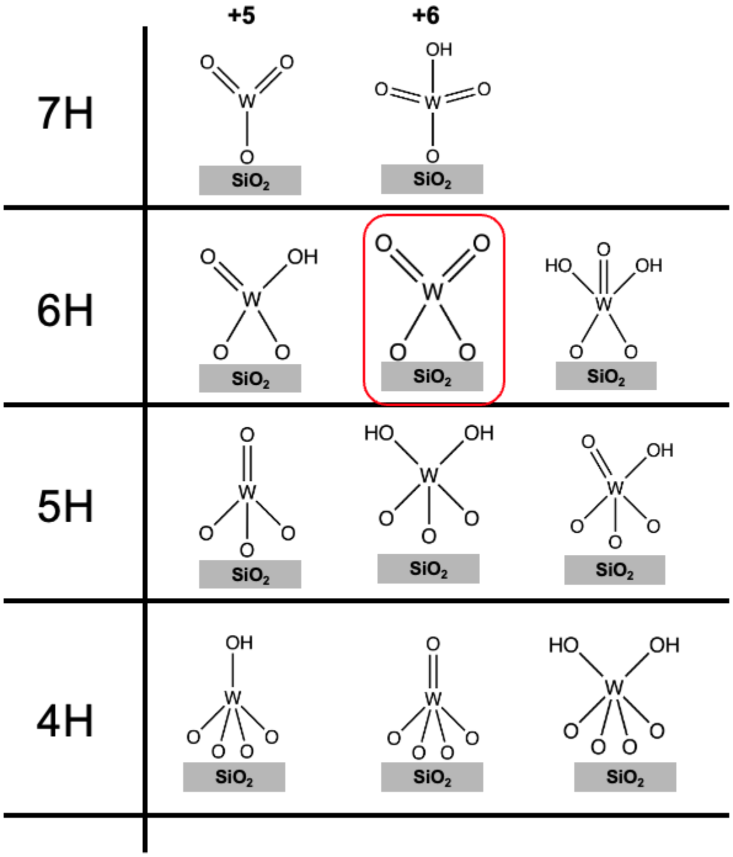

Figure S23: Different configurations of silica-supported WO_x monomer with +5 and +6 W formal oxidation states. Number on left indicates the number of H atoms on the surface.

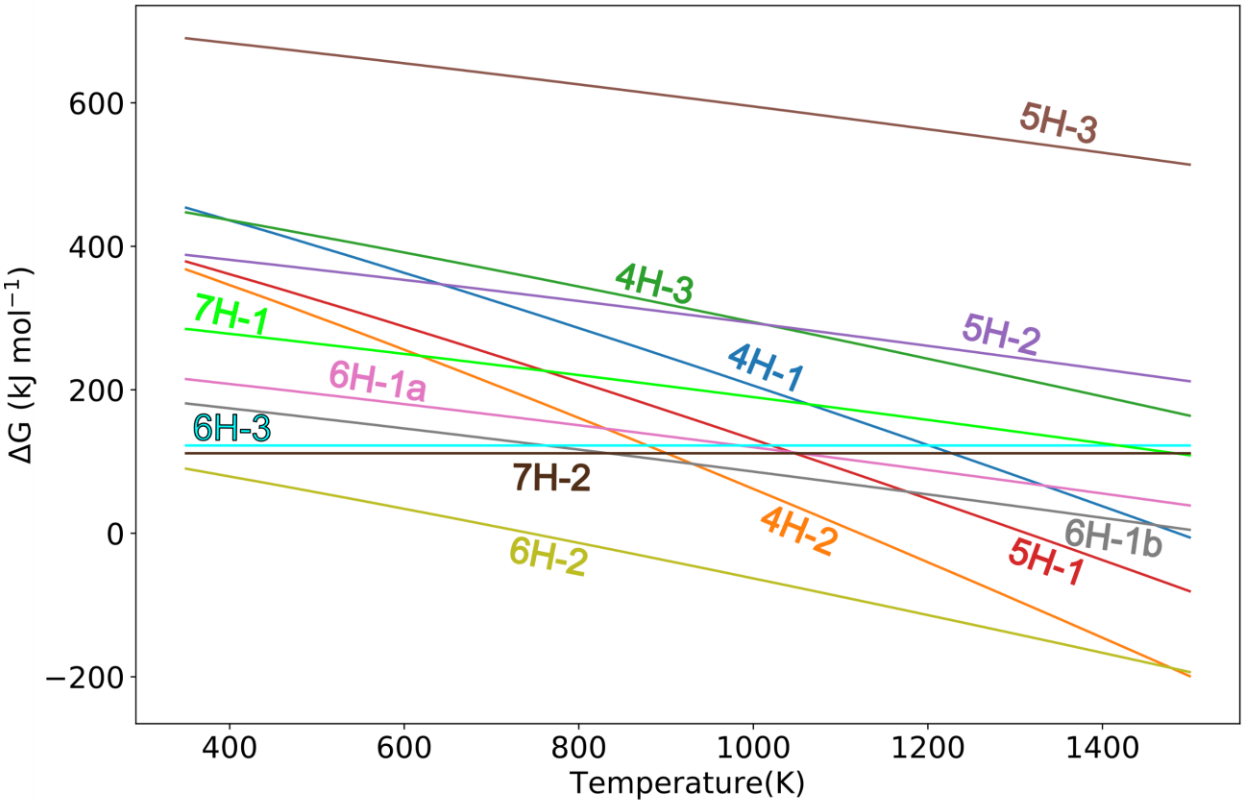

Figure S24: Free energy diagram for the different configurations considered in **Figure S23** under synthesis conditions (0.01 kPa H_2O and 20 kPa O_2). 6H-1a and 6H-1b indicate different configurations of the same stoichiometry. -1, -2, -3 indicate the first, second and third configuration in **Figure S23**. 4, 5, 6, 7 indicate the number of H atoms on the surface.

Dimers:

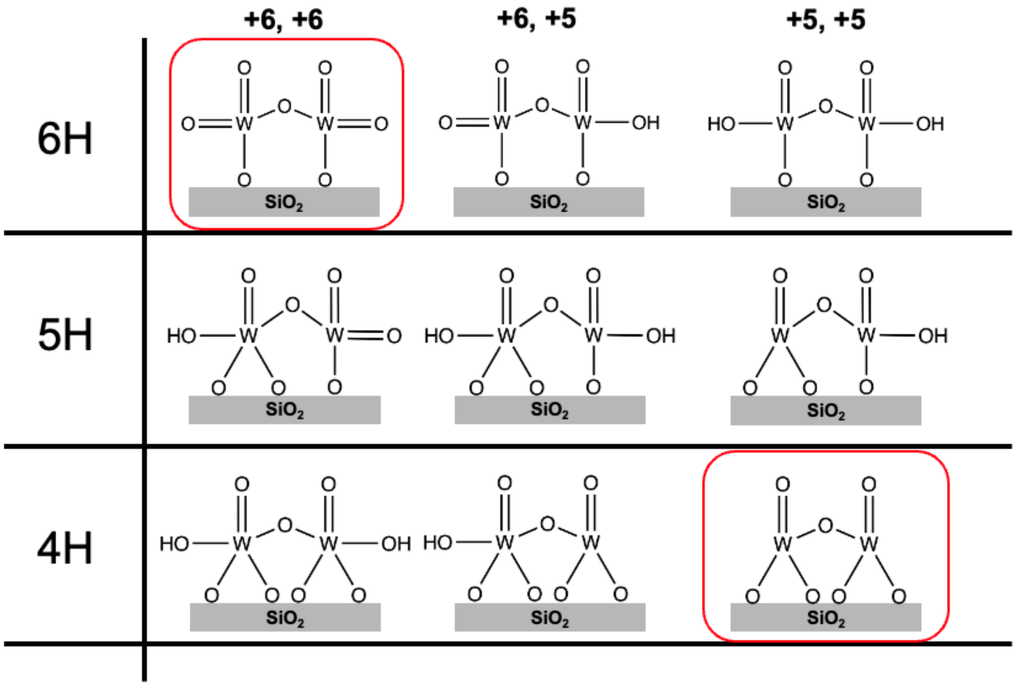

Figure S25: Different configurations of silica-supported WO_x dimer with +5 and +6 W formal oxidation states. Number on left indicates the number of H atoms on the surface.

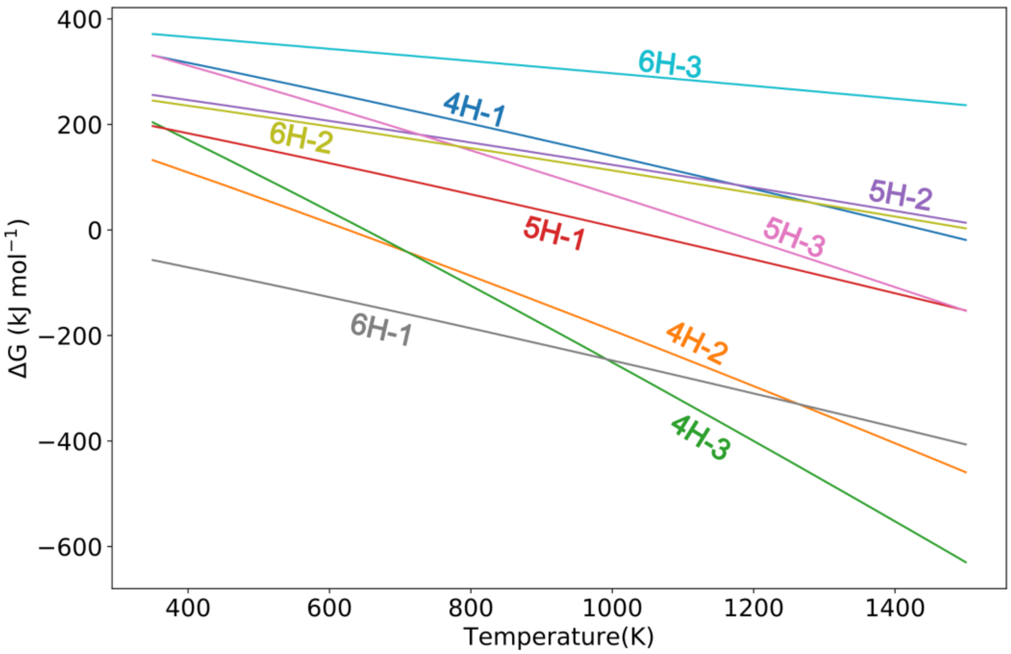

Figure S26: Free energy diagram for the different configurations considered in **Figure S25** under synthesis conditions (0.01 kPa H_2O and 20 kPa O_2). -1, -2, -3 indicate the first, second and third configuration in **Figure S25**. 4, 5, 6, 7 indicate the number of H atoms on the surface.

Trimers:

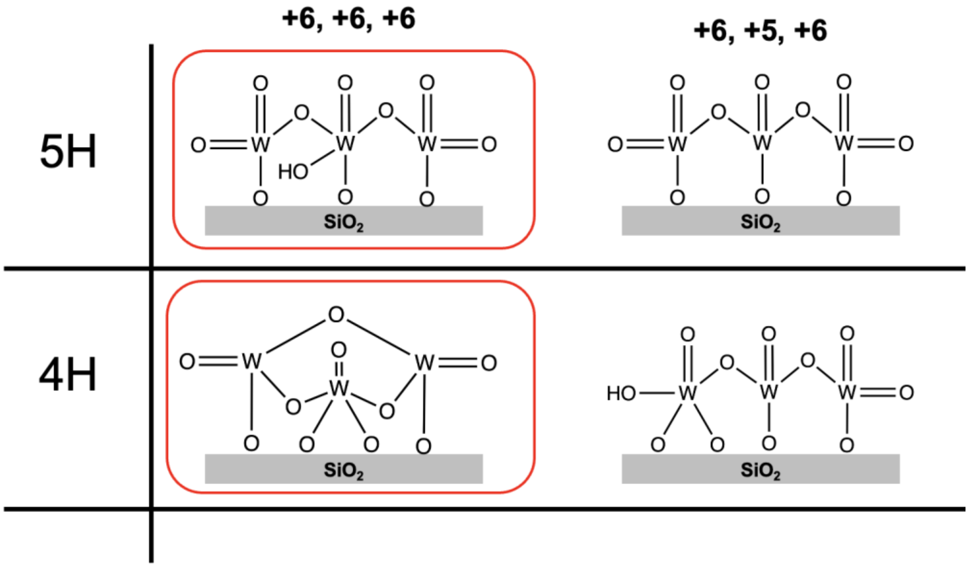

Figure S27: Different configurations of silica-supported WO_x trimer with +5 and +6 W formal oxidation states. Number on left indicates the number of H atoms on the surface.

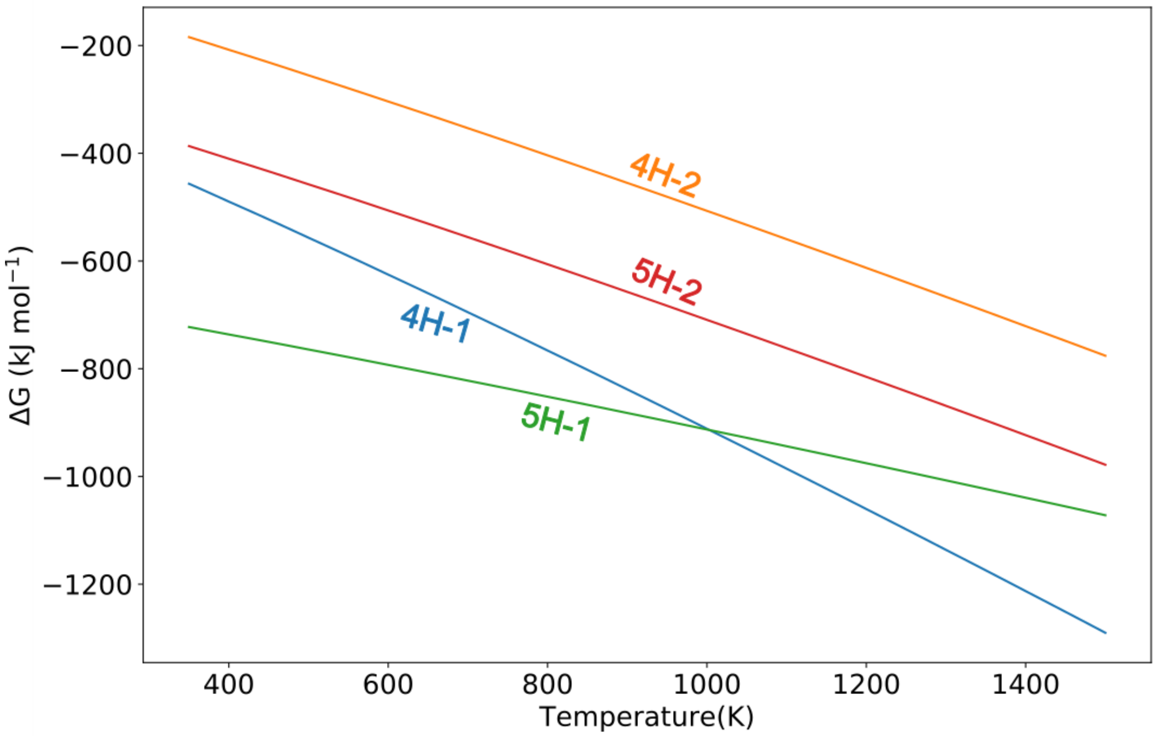

Figure S28: Free energy diagram for the different configurations considered in **Figure S27** under synthesis conditions (0.01 kPa H_2O and 20 kPa O_2). -1, -2 indicate the first, and second configuration in **Figure S27**. 4, 5, 6, 7 indicate the number of H atoms on the surface

S.2.4: Phase Diagrams for Combined Domain Sizes on SiO_2

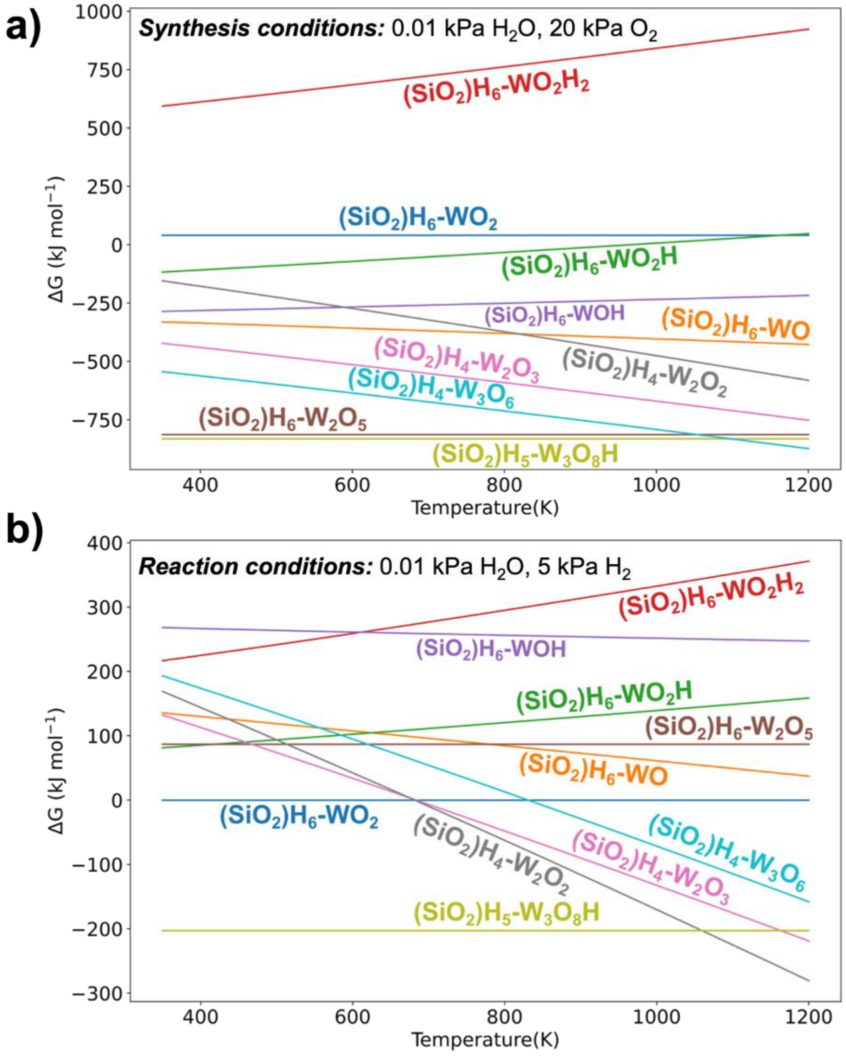

Figure S29: Free energy diagram for all sizes of tungsten oxide cluster on silica support under a) synthesis conditions and b) reaction conditions. These figures contain all structures initially generated and from cascade reactions for H addition and O removal shown in **Figure 9**.

S.2.5: WO_x Supported on Silica with HSE06

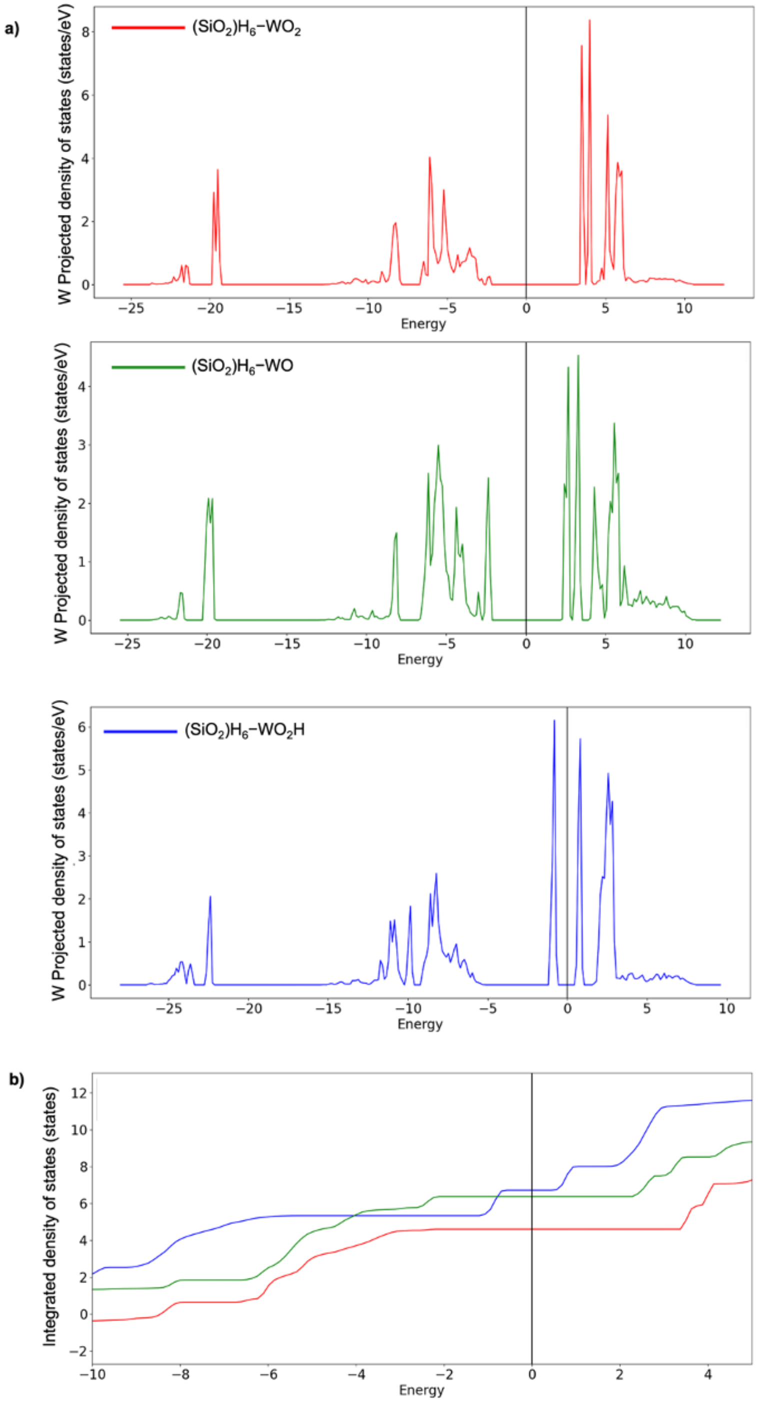

Figure S30: a) Projected W DOS for silica-supported WO_x monomers. b) Integrated projected W DOS. Computed using the HSE06 functional.

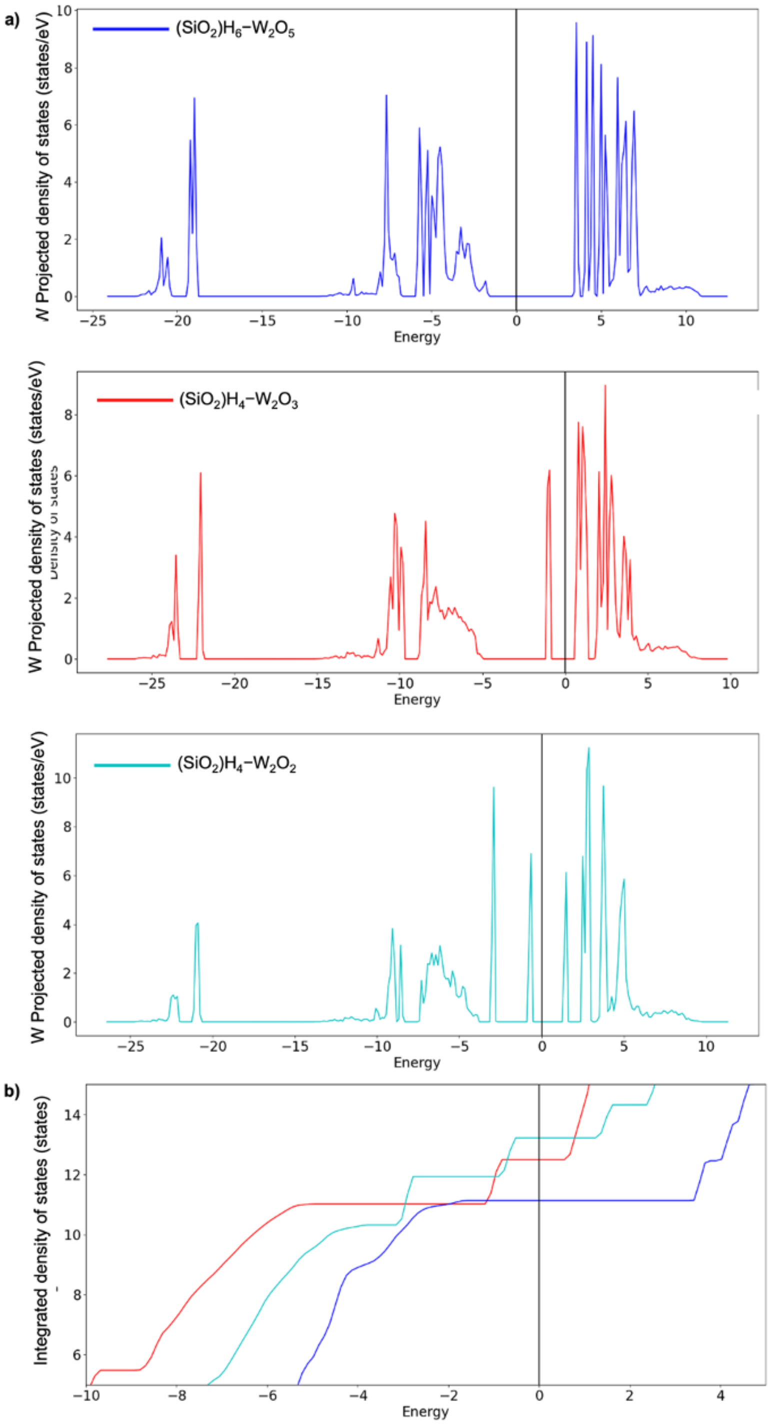

Figure S31: a) Projected W DOS for silica-supported WO_x dimers. b) Integrated projected W DOS. Computed using the HSE06 functional.

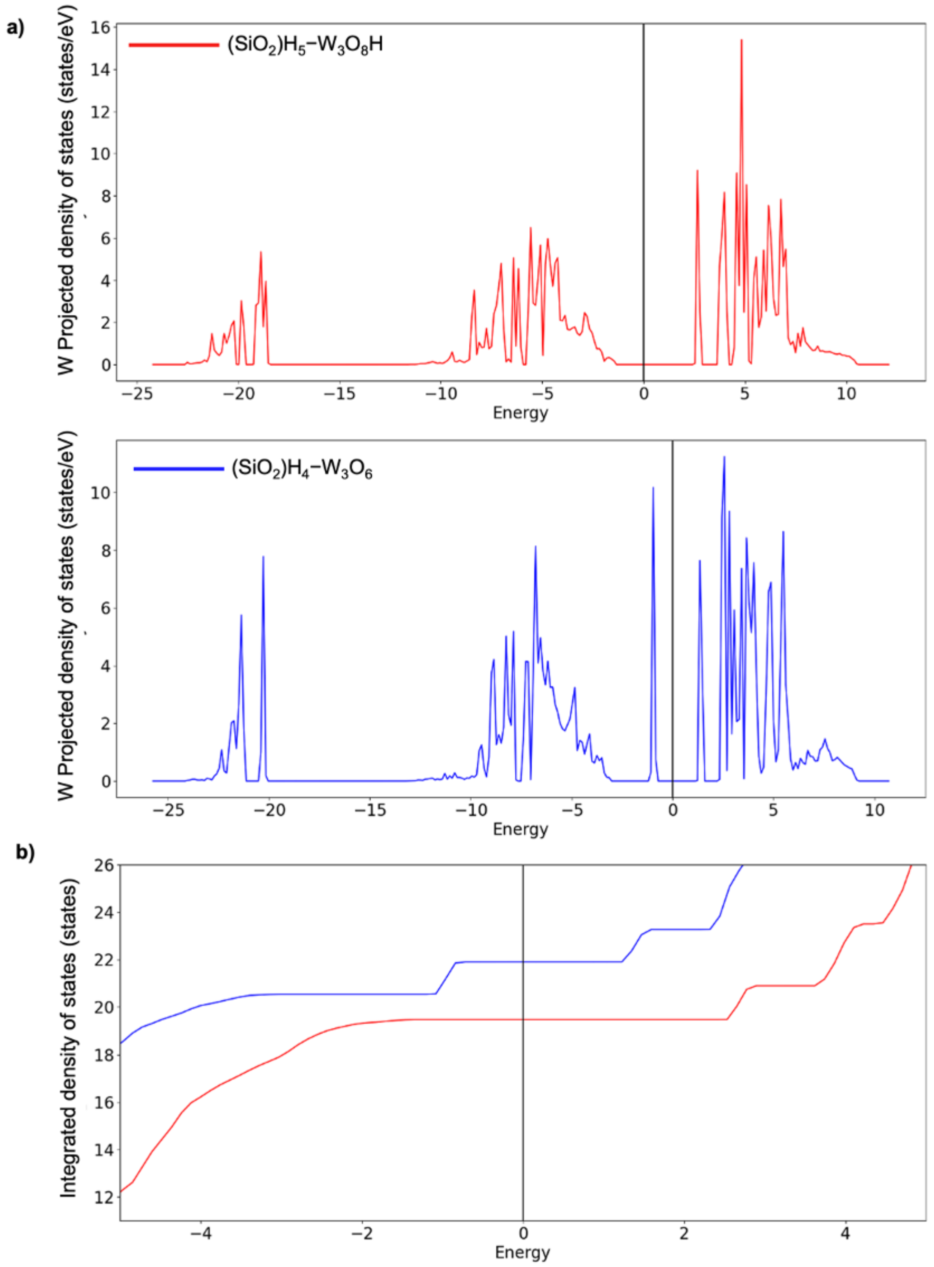

Figure S32: a) Projected W DOS for silica-supported WO_x trimers. b) Integrated projected W DOS. Computed using the HSE06 functional.

S.2.6: Phase Diagrams for Combined Domain Sizes on TiO_2

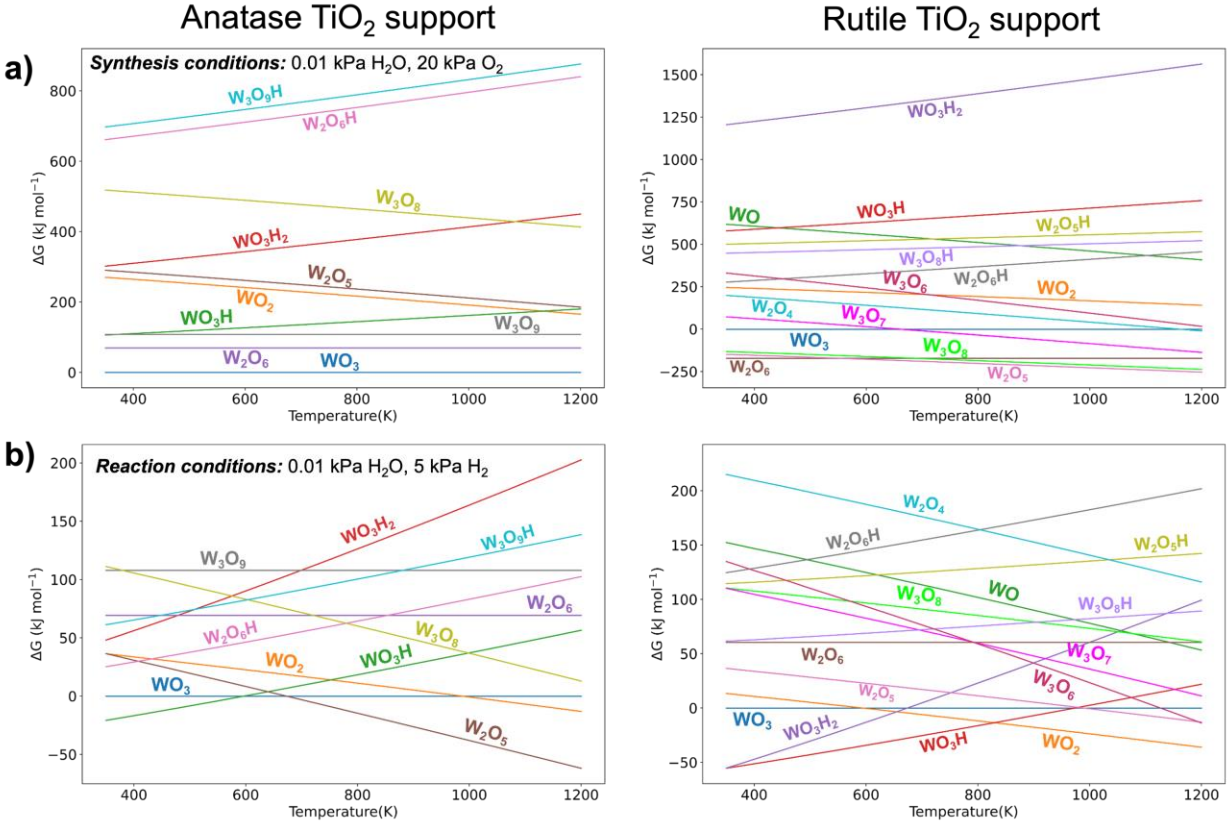

Figure S33: Free energy for all sizes of tungsten oxide cluster on both anatase and rutile titania support under a) synthesis conditions and b) reaction conditions. These figures contain all structures initially generated and from cascade reactions for H addition and O removal shown in **Figure 11**.

S.2.7: Vacancy Generation on Rutile TiO_2

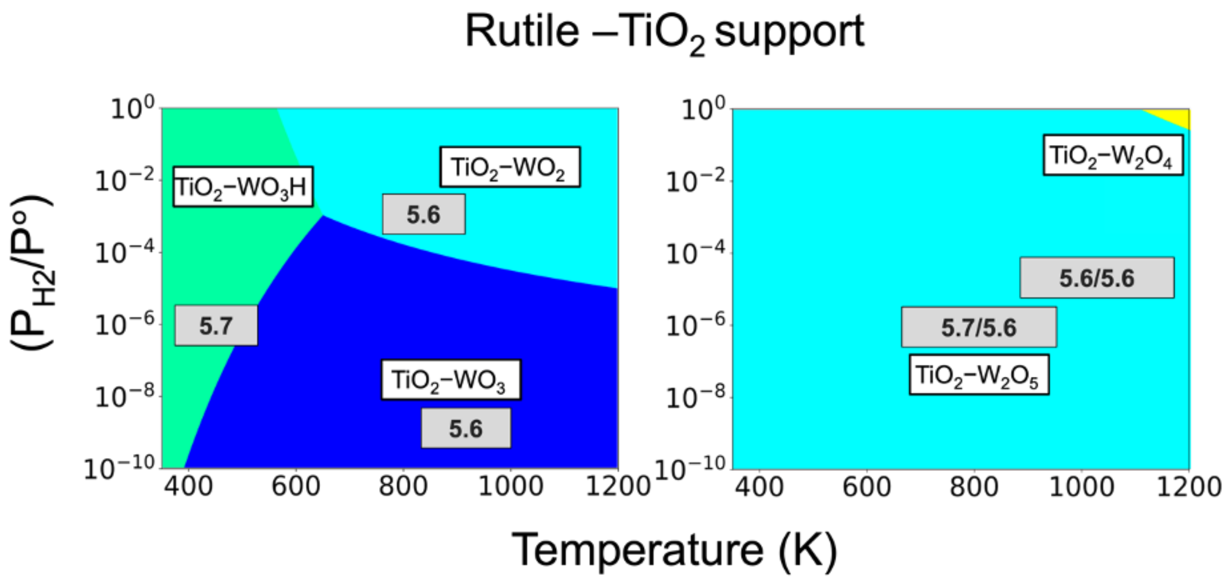

Figure S34: Ab initio thermodynamic phase diagram for rutile TiO_2 supported WO_x monomer, and dimer with surface O vacancies. Computed with SCAN functional.

To explore the effect of the surface oxygen vacancies on the relative stability of the WO_x clusters, we generated O vacancies on the TiO_2 surface that were in the proximity of the WO_x clusters and subsequently recomputed a subset of phase diagrams. To generate O vacancies on the surface, we started from the structure with no initial surface vacancies that was the lowest free energy. We then systematically removed surface O atoms that were within 2.3 Å any atom in the WO_x cluster but not directly bonded to W. Each of these structures is optimized using the same convergence criteria as previously reported in **Section 2.3**. We then took the structure with the lowest energy for each WO_x cluster and generated a new phase diagram (using the SCAN functional for computational efficiency) following the methods described in **Section 2.3**. Our results indicate that the presence of surface vacancies does not significantly affect the relative free energies of the WO_x monomer and dimer clusters supported on rutile TiO_2 in comparison to the diagrams computed with SCAN reported in **Figure S44a.** Notably, the oxidation state of W in the WO_x clusters did not vary significantly with the presence of an oxygen vacancy on the surface.

S.2.8: WO_x Supported on Anatase Titania with HSE06

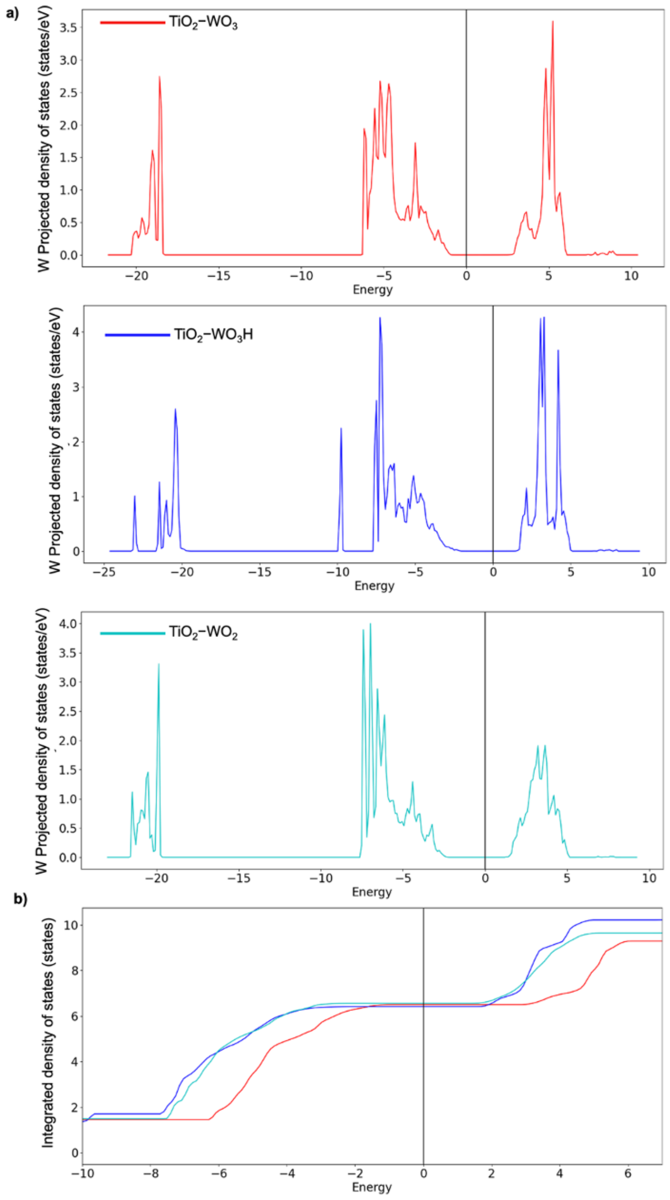

Figure S35: a) Projected W DOS for anatase titania-supported WO_x monomers. b) Integrated projected W DOS. Computed using the HSE06 functional.

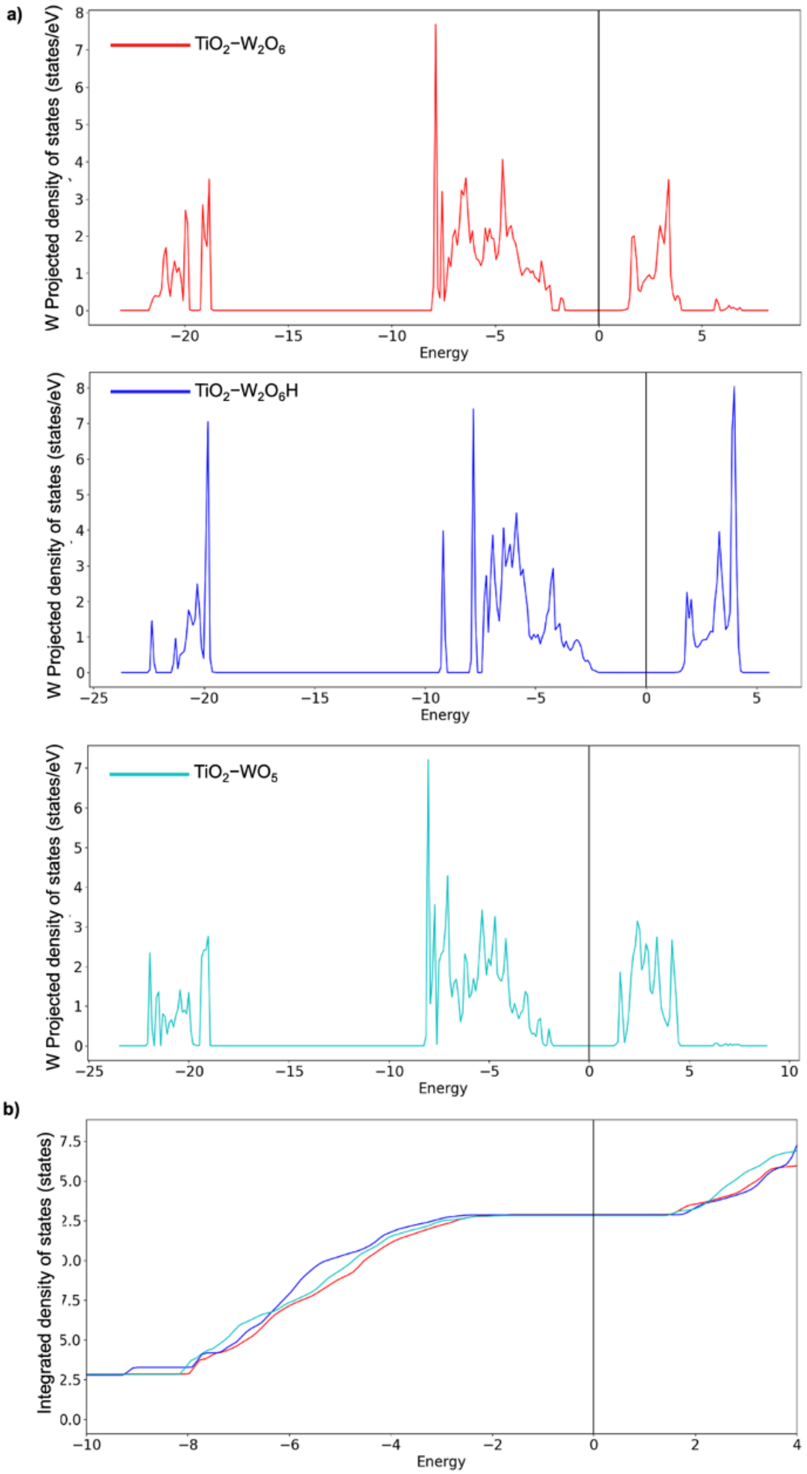

Figure S36: a) Projected W DOS for anatase titania-supported WO_x dimers. b) Integrated projected W DOS. Computed using the HSE06 functional.

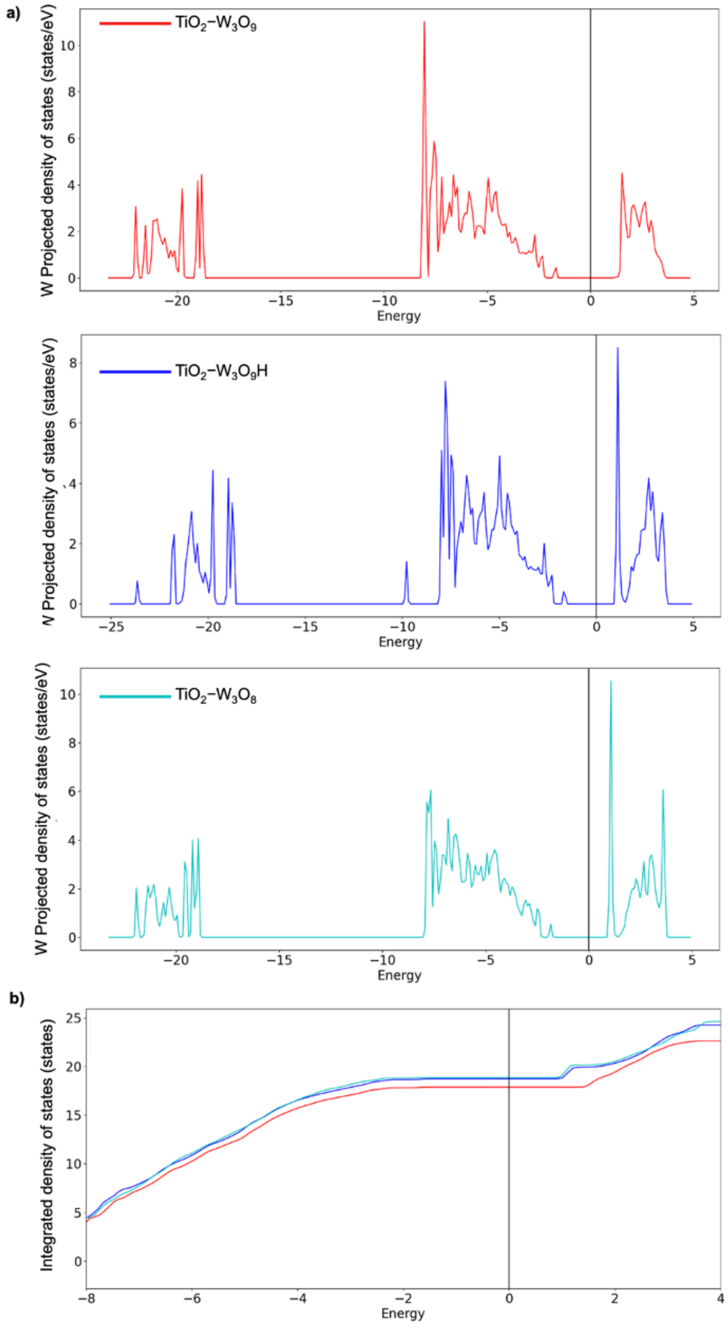

Figure S37: a) Projected W DOS for anatase titania-supported WO_x trimers. b) Integrated projected W DOS. Computed using the HSE06 functional.

S.2.9: WO_x Supported on Rutile Titania with HSE06

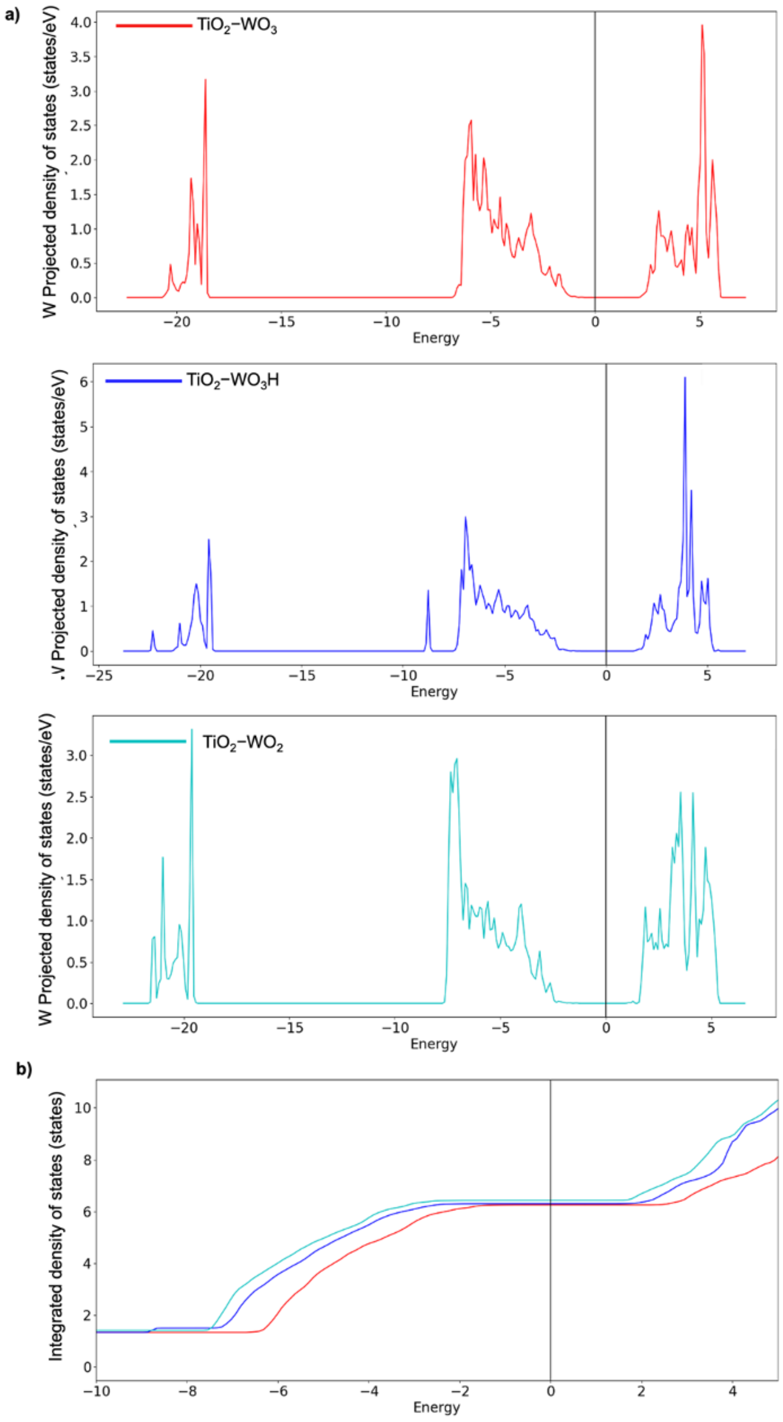

Figure S38: a) Projected W DOS for rutile titania-supported WO_x monomers. b) Integrated projected W DOS. Computed using the HSE06 functional.

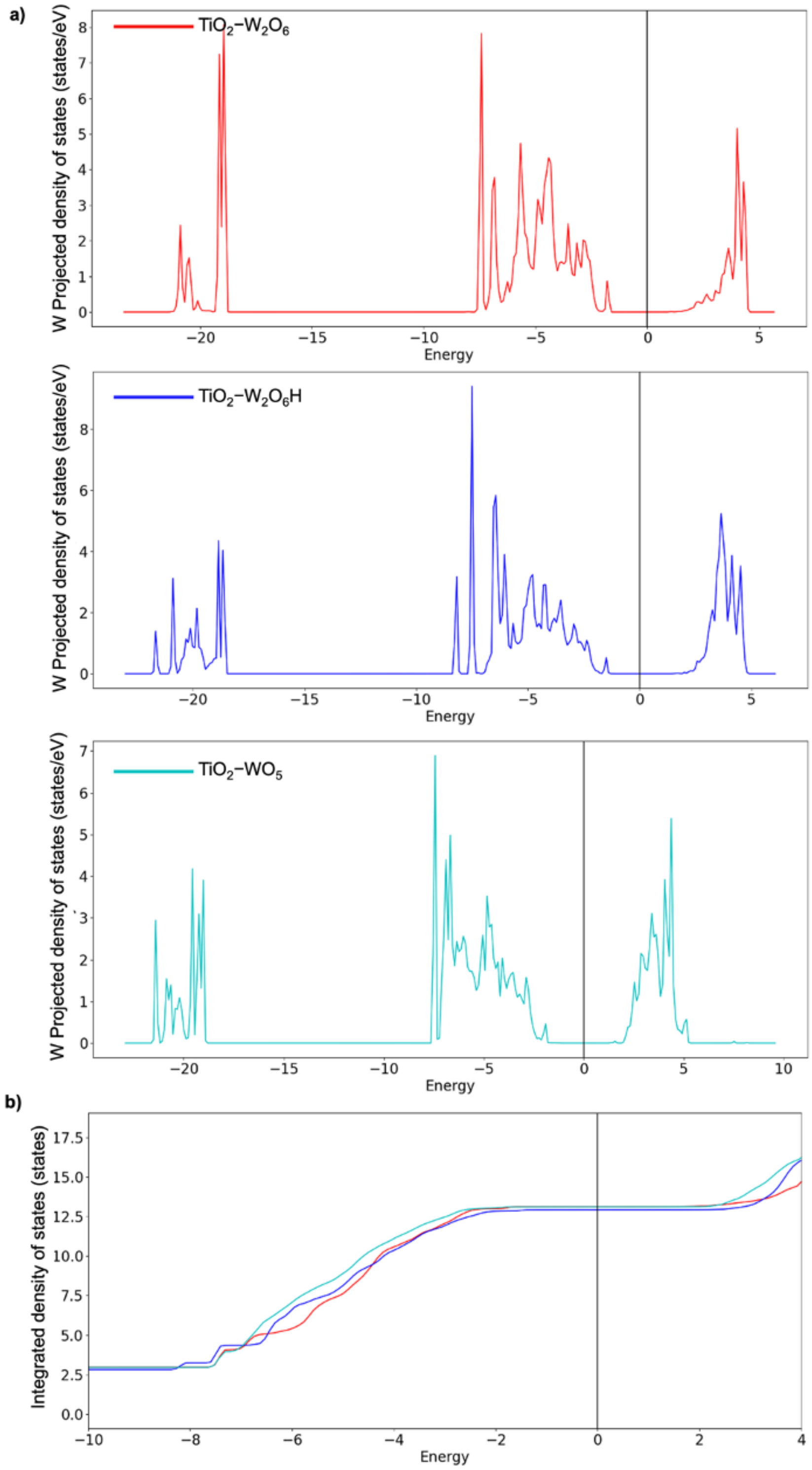

Figure S39: a) Projected W DOS for rutile titania-supported WO_x dimers. b) Integrated projected W DOS. Computed using the HSE06 functional.

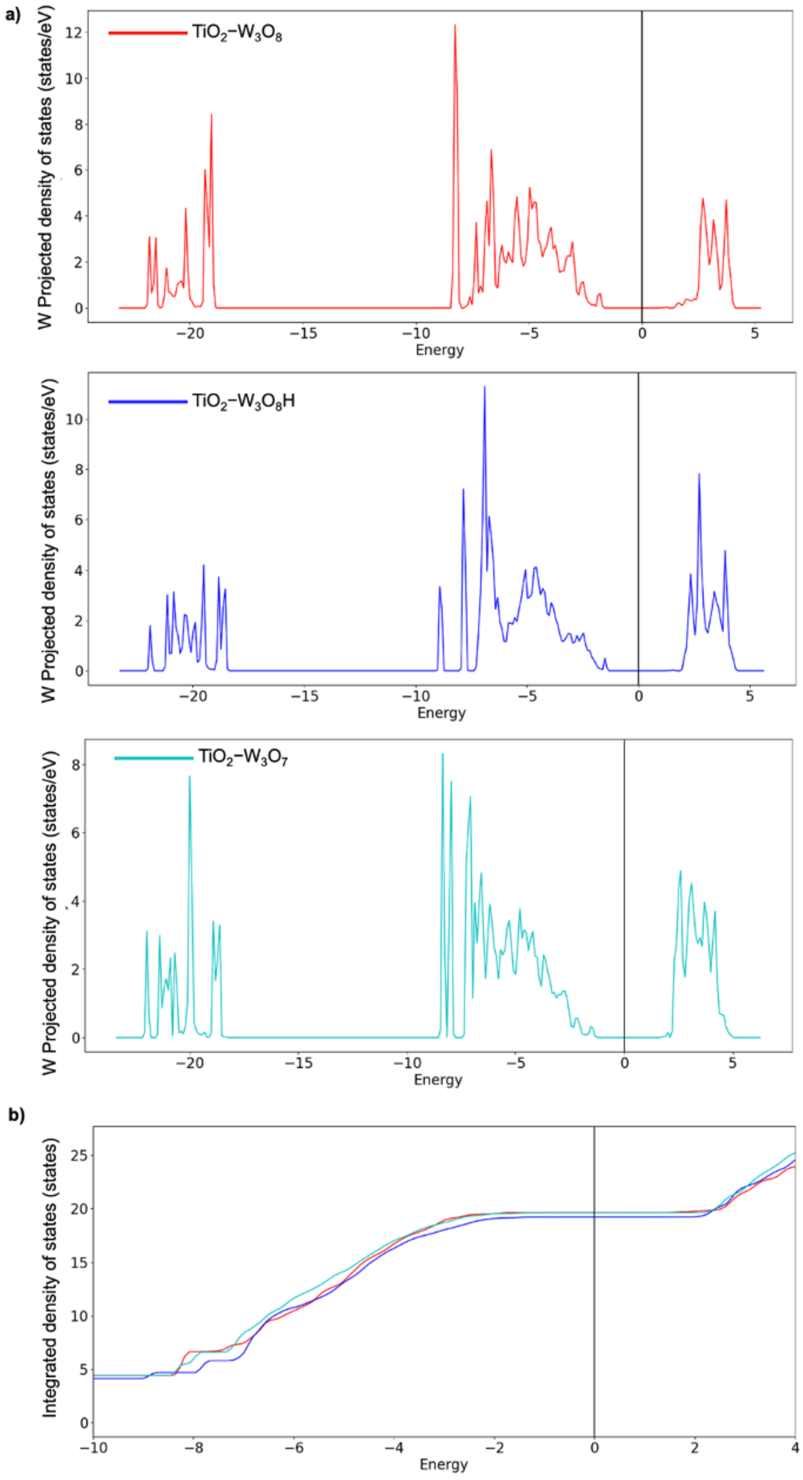

Figure S40: a) Projected W DOS for rutile titania-supported WO_x trimers. b) Integrated projected W DOS. Computed using the HSE06 functional.

S.2.10: Charge Differences for O Removal Reaction

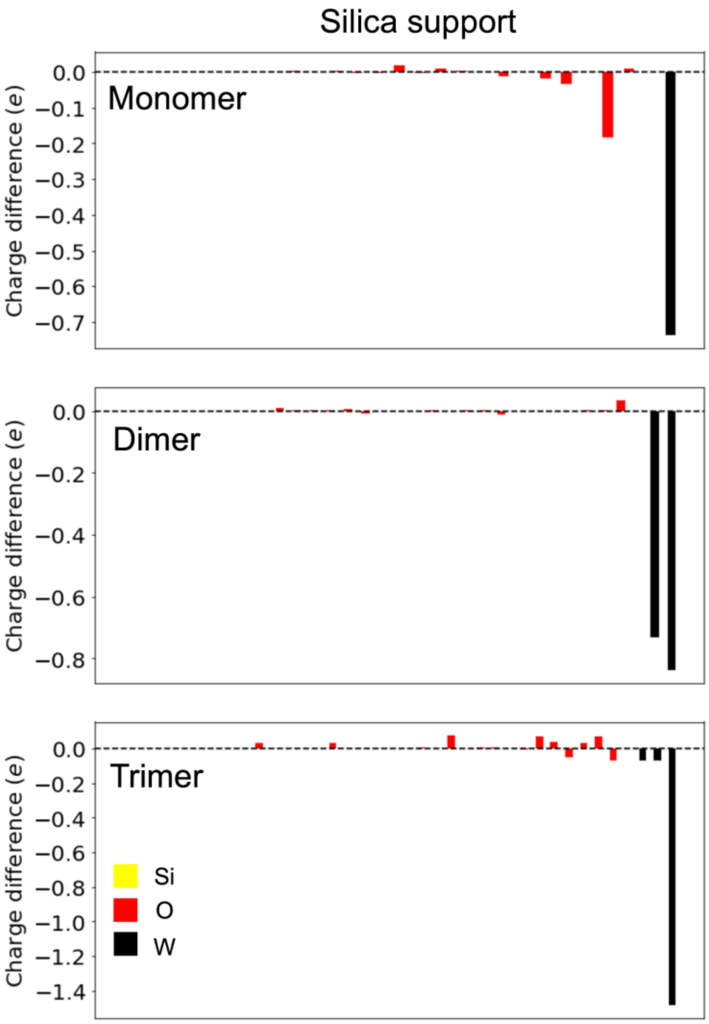

Figure S41: Differences in charge density of surface atoms on silica support following O-removal. There are no silica atoms with charge differences, hence the lack of yellow bars in the figure. Generated using the HSE06 functional.

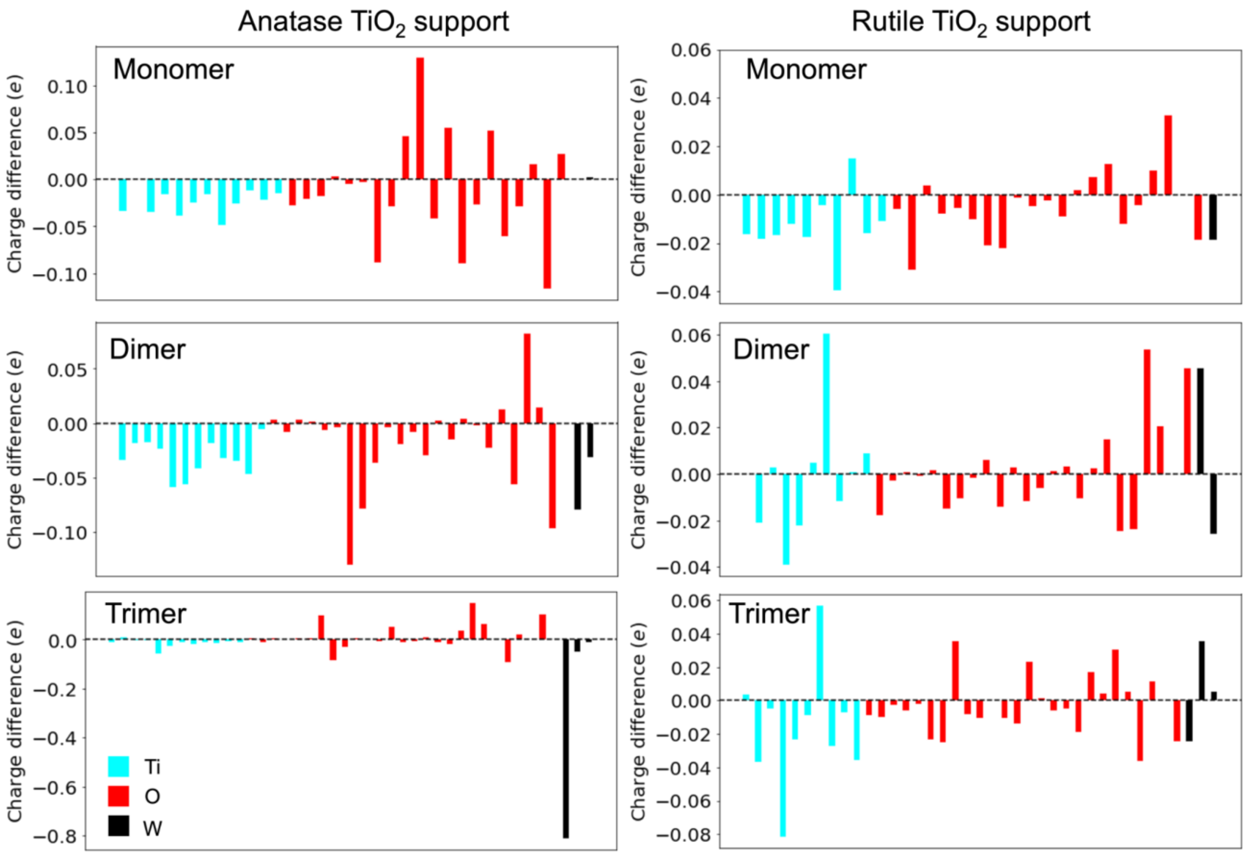

Figure S42: Differences in charge density of surface atoms on anatase and rutile titania support following O-removal. Note that the magnitude of the y-axis scales is smaller than **Figure S41**. Generated using the HSE06 functional.

S.2.11: Phase Diagrams and Oxidation States Using SCAN

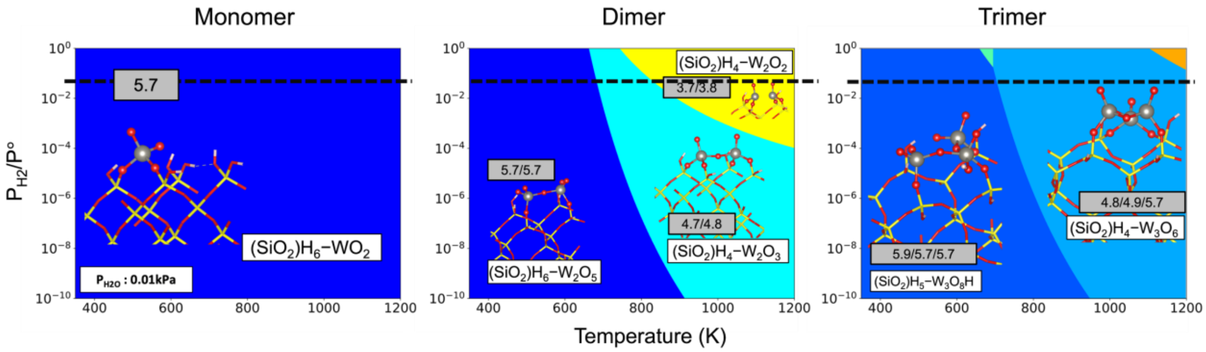

Figure S43: Ab initio thermodynamic phase diagram for silica supported WO_x monomer, dimer, and trimer at P_{H2O} = 0.01 kPa. Gray boxes report the oxidation state of W. Computed using the SCAN functional.

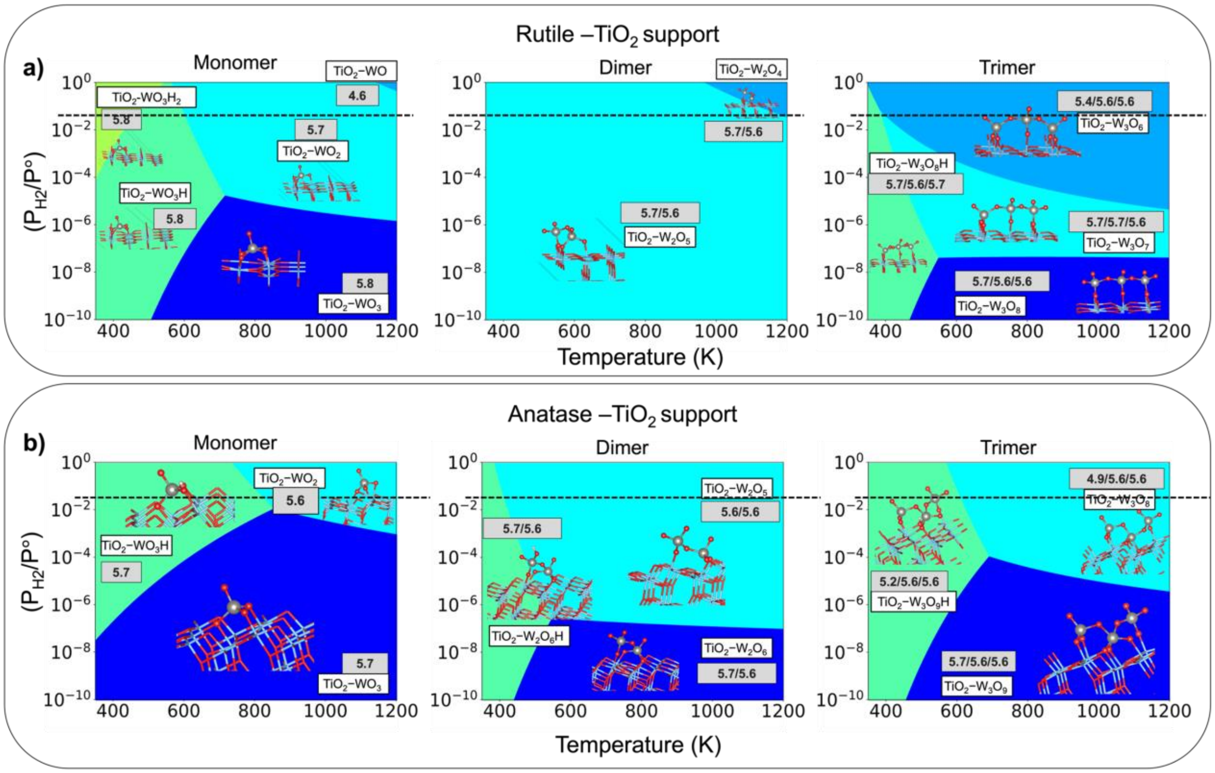

Figure S44: a) Ab initio thermodynamic phase diagram for rutile TiO_2 supported WO_x monomer, dimer, and trimer. **b)** Ab initio thermodynamic phase diagram for anatase TiO_2 supported WO_3 monomer, dimer, and trimer. Gray boxes report the oxidation state of W. Computed using the SCAN functional.

S.2.12: Density of States

The projected W DOS were generated using LORBIT = 11, and the Blöchl-Tetrahedron method (ISMEAR = -5). Figures in **Section S.2.5** were generated using the HSE06 functional and **Figure S45** was generated using the SCAN functional.

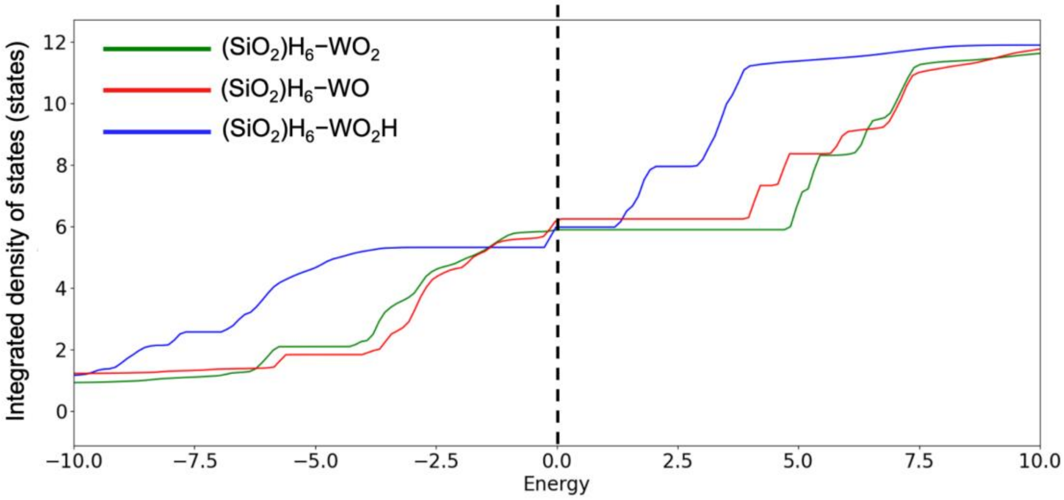

Figure S45: Integrated projected W DOS for silica-supported WO_x monomers, generated using the SCAN functional. Compared to the HSE analysis shown in **Figure S30b**, the integrated DOS at the Fermi Level (set to 0) does not show a significant difference between monomer species.

S.2.13: Thermodynamic Relations

To determine the most thermodynamically stable W speciation on the support at different synthesis ($\mu_{O_2,}\mu_{H_2O}$) and reaction conditions ($\mu_{H_2,}\mu_{H_2O}$), we evaluated the free energy of all the structures that we considered (**Section 2.3.1 and Section 2.3.2**). For the W clusters supported on TiO_2, we calculated the free energy under synthesis conditions as:

$$\mathrm{aWO_3} + \mathrm{TiO_2} + \left(\frac{y}{2}\right)\mathrm{H_2O} \rightarrow \mathrm{TiO_2W}_a\mathrm{O}_{3a-x}\mathrm{H}_y + \left(\frac{x}{2}+\frac{y}{4}\right)\mathrm{O_2} \quad (3)$$

$$\Delta G(\mu_{O_2,}\mu_{H_2O}) = E_{\mathrm{TiO_2W}_a\mathrm{O}_{3a-x}\mathrm{H}_y} - aE_{\mathrm{WO_3}} - E_{\mathrm{surface}} + x\left(\frac{\mu_{O_2}}{2}\right) - y\left(\frac{\mu_{H_2O}}{2} - \frac{\mu_{O_2}}{4}\right) \quad (4)$$

Similarly, the free energy under reaction conditions is:

$$\mathrm{aWO_3} + \mathrm{TiO_2} + \left(x+\frac{y}{2}\right)\mathrm{H_2} \rightarrow \mathrm{TiO_2W}_a\mathrm{O}_{3a-x}\mathrm{H}_y + x\mathrm{H_2O} \quad (5)$$

$$\Delta G(\mu_{H_2,}\mu_{H_2O}) = E_{\mathrm{TiO_2W}_a\mathrm{O}_{3a-x}\mathrm{H}_y} - aE_{\mathrm{WO_3}} - E_{\mathrm{surface}} - y\left(\frac{\mu_{H_2}}{2}\right) + x(\mu_{H_2O} - \mu_{H_2}) \quad (6)$$

$E_{\mathrm{TiO_2W}_a\mathrm{O}_{3a-x}\mathrm{H}_y}$is the DFT-computed energy of the given supported WO_x cluster, E_{WO_3} is the energy of the bulk WO_3 and E_{surface} is the energy of the TiO_2 surface. Here, *a* is the number of W atoms, *x* is the number of O atoms removed and *y* is the number of H atoms added. We define $\mu_{H_2}^{\mathrm{ref}} = E_{H_2}^{\mathrm{ref}}, \mu_{H_2O}^{\mathrm{ref}} = \mathrm{E}_{H_2O}^{\mathrm{ref}}$ and $\mu_{O_2}^{\mathrm{ref}} = E_{O_2}^{\mathrm{ref}}$.

Similarly, Equations 8 and 10 give the free energy under synthesis conditions and reaction conditions, respectively, for the SiO_2 support:

$$\mathrm{aWO_3} + \mathrm{SiO_2H_8} + \left(\frac{y}{2}\right)\mathrm{H_2O} + \left(\frac{c}{4}\right)\mathrm{O_2} \rightarrow \mathrm{SiO_2W}_a\mathrm{O}_{3a-x}\mathrm{H}_{8-c+y} + \left(\frac{x}{2}+\frac{y}{4}\right)\mathrm{O_2} + \left(\frac{c}{2}\right)\mathrm{H_2O} \quad (7)$$

$$\Delta G(\mu_{O_2,}\mu_{H_2O}) = E_{\mathrm{SiO_2W}_a\mathrm{O}_{3a-x}\mathrm{H}_{8-c+y}} - aE_{\mathrm{WO_3}} - E_{\mathrm{surface}} + \left(x+\frac{y}{2}-c\right)\left(\frac{\mu_{O_2}}{2}\right) - (y-c)\left(\frac{\mu_{H_2O}}{2}\right) \quad (8)$$

$$\mathrm{aWO_3} + \mathrm{SiO_2H_8} + \left(x+\frac{y}{2}\right)\mathrm{H_2} \rightarrow \mathrm{SiO_2W}_a\mathrm{O}_{3a-x}\mathrm{H}_{8-c+y} + x\mathrm{H_2O} + \left(\frac{c}{2}\right)\mathrm{H_2} \quad (9)$$

$$\Delta G(\mu_{H_2,}\mu_{H_2O}) = E_{\mathrm{SiO_2W}_a\mathrm{O}_{3a-x}\mathrm{H}_{8-c+y}} - aE_{\mathrm{WO_3}} - E_{\mathrm{surface}} - (y-c)\left(\frac{\mu_{H_2}}{2}\right) + x(\mu_{H_2O} - \mu_{H_2}) \quad (10)$$

$E_{\mathrm{SiO_2W}_a\mathrm{O}_{3a-x}\mathrm{H}_{8-y}}$is the DFT-computed energy of the given supported WO_x cluster, E_{WO_3} is the energy of the bulk WO_3 and E_{surface} is the energy of the SiO_2 surface. Here, *a* is the number of W atoms, *x* is the number of O atoms removed, *y* is the number of H atoms added and c is the number of H atoms removed from the SiO_2 surface.

S.2.14: Cell Size Effects on Rutile TiO_2

To test if interactions from periodic images affect computed energies for WO_x clusters, we used the W_3O_8 trimer supported on rutile TiO_2 as a test case because we anticipate that periodic interactions would be more substantial for the larger clusters. We compared the grafting energy (Equation XYZ) for the trimer on 3x2, 3x3 and 4x2 rutile TiO_2 supercells (**Table S15**). We found that there was minimal change in the grafting energy of the WO_x trimer with the increased cell size. The grafting energy is calculated using:

$$3\mathrm{WO_3} + \mathrm{TiO_2} \rightarrow \mathrm{TiO_2W_3O_8} + \frac{\mathrm{O_2}}{2} \quad (11)$$

$$\Delta G = E_{\mathrm{TiO_2W_3O_8}} - 3E_{\mathrm{WO_3}} - E_{\mathrm{TiO_2}} + \left(\frac{\mu_{\mathrm{O_2}}}{2}\right) \quad (12)$$

$E_{\mathrm{TiO_2W_3O_8}}$ is the DFT-computed energy of the given supported WO_x trimer, $E_{\mathrm{WO_3}}$ is the energy of the bulk WO_3 and $E_{\mathrm{TiO_2}}$ is the energy of the TiO_2 surface.

Table S15: Grafting energy for WO_x trimer on different rutile TiO_2 surface supercell size

Surface	Grafting Energy (kJ mol^{-1})
3x2	-206
3x3	-204
4x2	-203

S.3: References

(1) Sandupatla, A. S.; Alexopoulos, K.; Reyniers, M.-F.; Marin, G. B. DFT Investigation into Alumina ALD Growth Inhibition on Hydroxylated Amorphous Silica Surface. *The Journal of Physical Chemistry C* **2015**, *119* (32), 18380-18388. DOI: 10.1021/acs.jpcc.5b05261.

(2) Miu, E. V.; Mpourmpakis, G.; McKone, J. R. Predicting the Energetics of Hydrogen Intercalation in Metal Oxides Using Acid–Base Properties. *ACS Applied Materials & Interfaces* **2020**, *12* (40), 44658-44670. DOI: 10.1021/acsami.0c11300.
